# Synthesis of Epoxyoxirenes: Phytotoxic Activity and Enzymatic Target Identification

**DOI:** 10.3390/plants14131933

**Published:** 2025-06-24

**Authors:** Kamylla C. F. de Faria, Elson S. Alvarenga, Denilson F. Oliveira, Vitor C. Baia, Armin F. Isenmann

**Affiliations:** 1 Department of Chemistry, Universidade Federal de Viçosa, Viçosa 36570-900, MG, Brazil; kamyllacalzolariferreira@gmail.com (K.C.F.d.F.); vitor@ufv.br (V.C.B.); 2 Department of Chemistry, Universidade Federal de Lavras, Lavras 37203-202, MG, Brazil; denilson@ufla.br; 3 Department of Metallurgy and Chemistry, Centro Federal de Educação Tecnológica de Minas Gerais, Timoteo 35180-008, MG, Brazil; armincefetmg@gmail.com

**Keywords:** epoxyoxirenes, herbicides, phytotoxicity, Diels–Alder reaction, *in silico*, protein target, tubulin

## Abstract

Chemical control is key to minimizing agricultural losses, driving the search for more efficient and selective herbicides. This study reports the synthesis of epoxyoxirenes, their phytotoxic evaluation, and an *in silico* analysis to identify the protein target of the most active compound in plants. Compounds 2–19 were tested on *Lactuca sativa* spp., *Allium cepa* spp., *Cucumis sativus* spp., *Triticum aestivum*, and *Bidens pilosa*. The synthetic route began with anhydride **1**, obtained via a Diels–Alder reaction between maleic anhydride and furan (91% yield). Anhydride **1** was converted into amides **2**–**7** through reactions with aromatic amines (>92% yields), followed by cyclization to imides **8**–**13** (60–83% yields), and subsequent epoxidation to afford epoxides **14**–**19** (62–98% yields). All the compounds interfered with seedling development, with compounds **2**–**7** showing the greatest phytotoxicity to *B. pilosa* at concentrations of 500 μM and 1000 μM. An *in silico* analysis suggested plant tubulin as a potential protein target for the most active compounds. These findings highlight epoxyoxirenes as promising scaffolds for novel herbicide development and support further investigation into their mechanism of action.

## 1. Introduction

Weeds are a critical factor in reducing agricultural productivity, either by interfering with crop development during early growth stages or by competing for essential resources such as nutrients, water, light, and space [1,2]. Additionally, weeds tend to be more resistant and tolerant to various biotic and abiotic stressors. Global estimates suggest that potential yield losses due to weeds range from 60% in wheat to 80% in cotton [3,4]. The most effective strategy for minimizing economic losses in agriculture is chemical control, particularly using herbicides [5,6].

However, the repeated or indiscriminate use of the same chemical controls has led to the development of herbicide resistance in certain weed species [7,8]. Currently, around 260 weed species have been reported as herbicide-resistant, with over 500 unique cases documented across 70 countries [9,10]. To address this growing problem, it is essential to rotate herbicides and integrate them with established weed management practices.

The discovery of new chemical controls that are more efficient, less toxic, and less persistent is of the utmost need to combat the increasing rates of resistance already known. Molecules derived from natural products, such as lactones and amides, stand out in the literature for exhibiting broad biological activities [11,12,13,14,15,16,17,18]. In addition to their easy synthetic access, the presence of functional groups with different reactivities allows for structural modifications to produce derivatives with biological potential for use in various sectors, especially in the agrochemical industry.

**Figure 1:**
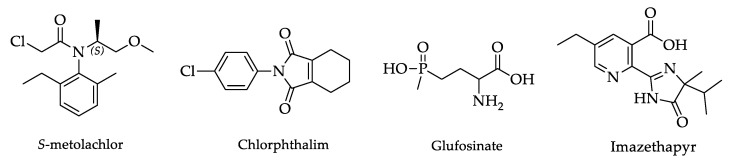
Commercial herbicides.

Epoxy groups combined with organic functions such as amides and imides, for example, are known in the literature for exhibiting significant biological activities, making them the focus of various scientific research efforts. There are several commercially available herbicides that contain amide, imide, amine, or carboxylic acid in their chemical structures, such as *S*-metolachlor, chlorphthalim, glufosinate, and imazethapyr [19,20,21] (Figure 1).

*S*-metolachlor is a pre-emergent herbicide from the acetanilide class, effective mainly in controlling grasses and some broadleaves, acting to inhibit the biosynthesis of essential fatty acids. Chlorphthalim belongs to the phthalimide group and has been used as a contact herbicide with variable selectivity. It is currently out of use, but may be available in some countries. Glufosinate is a non-selective contact herbicide that inhibits the enzyme glutamine synthetase and consequently photosynthesis; it is used both to control weeds and genetically modified crops that are resistant to it. Imazethapyr, belonging to the imidazolinone class, is a systemic herbicide that inhibits the enzyme acetohydroxyacid synthase (AHAS), which is essential for the biosynthesis of branched-chain amino acids and is effective in controlling weeds in pre- and post-emergence [22].

**Scheme 1:**
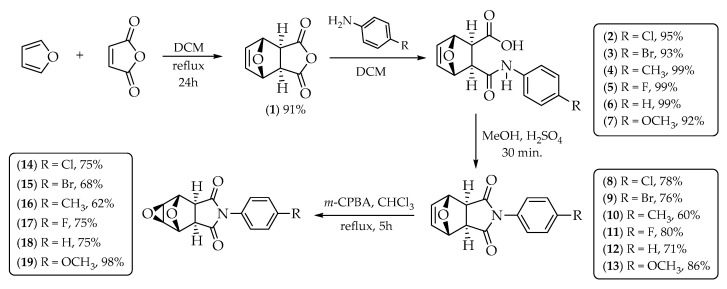
Syntheses of heterocyclic nitrogenated compounds **2**–**19** via Diels–Alder reaction and their yields.

Given the need to obtain new, more selective pesticides and motivated by the biological activity described in the literature for amides, cyclic imides, and epoxides, the main objective of this work was to select chemical structures among these compounds that are potentially useful for the development of new herbicides. To achieve this goal, this work presents the synthesis of eighteen heterocyclic nitrogenated compounds **2**–**19** derived from (3a*R*,4*S*,7*R*,7a*S*)-3a,4,7,7a-tetrahydro-4,7-epoxyisobenzofuran-1,3-dione **1** (Scheme 1). The compounds were evaluated for their phytotoxic activities through germination tests with seeds of *Lactuca sativa* spp., *Allium cepa* spp., *Cucumis sativus* spp., *Triticum aestivum*, and the weed species *Bidens pilosa*. These plants were chosen for their easy reproducibility and quick response time upon contact with the active ingredient at low concentrations. Finally, the enzymatic target of the substances showing the best phytotoxic activity was identified in silico.

## 2. Results and Discussion

### 2.1. Synthesis

The reaction between furan and maleic anhydride is a pericyclic Diels–Alder reaction that results in the reorganization of electrons in the substrate, forming the starting compound **1**. During the reorganization of electrons in the Diels–Alder reaction, the diene and the dienophile can interact in two distinct directions, leading to the formation of the *endo* and *exo* adducts, kinetic product, and thermodynamic product, respectively [23,24]. In this work, there was only the formation of the *exo* adduct, which was favored because of the heating carried out during the chemical reaction. The reaction can then be called *exo*-selective. The synthesis of *exo* adduct (**1**, 91% yield) was proven by IR, ^1^H NMR, and ^13^C NMR analyses.

The reaction that occurs between anhydride **1** and the amine can be classified as a nucleophilic addition to carbonyl carbon. The electron pair of the nucleophile, the nitrogen of the amine, attacks one of the electrophilic carbon atoms of compound 1, forming the amides/acids **2**–**7** with yields ranging from 92% to 99%. As anhydride **1** is symmetrical, the addition of the amine can be in any of the two carbonyls. The spectrometric data of these synthesized amides/acids are similar, as they differ only in one ligand in the aromatic ring.

The synthesized amides/acids **2**–**7** were subjected to a cyclization reaction, during which water was eliminated. Through this process, imides **8**–**13** were synthesized in 60–86% yields. The spectrometric data of these synthesized imides are similar, as they differ only in one ligand in the aromatic ring.

The imides **8**–**13** were subjected to the epoxidation reaction in the presence of *m*-chloroperbenzoic acid (*m*-CPBA 77%), thus forming the epoxides **14**–**19** with yields ranging from 62% to 98%. The spectrometric data of these synthesized epoxides are similar, as they differ only in one ligand in the aromatic ring (Scheme 1).

The most commonly used reagent in epoxidation reactions is *m*-chloroperbenzoic acid (*m*-CPBA, 77%). *m*-CPBA features an extra oxygen atom between the carbonyl group and its acidic hydrogen. The O-O bond weakens, promoting an electrophilic attack. The π-bond of the alkene, acting as a nucleophile, interacts with the electrophilic oxygen of the peroxide. The peroxide attacks the alkene on the side with less steric hindrance, forming two new C-O bonds. Therefore, the reaction is stereospecific. As a result of this strict stereospecificity, no isomer formation occurs during the reaction. The orientation and arrangement of atoms in the reactants allow only one specific stereoisomeric configuration to be achieved. Therefore, this level of control and predictability in epoxidation reactions is invaluable in the synthesis of various compounds, making significant contributions to the field of organic chemistry.

Compound **19** is novel. Therefore, its characterization is discussed below. The spectra for all compounds are shown in the Supplementary Materials (SM, Figures S1–S76).

Compounds **8**–**11** and **14**–**17** have been previously described in the scientific literature [25]. To confirm their identity, we compared the spectroscopic data (such as NMR and IR spectra) from our synthesized compounds with those reported in the literature. The comparison showed a strong correlation between the two sets of data, indicating that the structures of the compounds obtained in this study are consistent with those previously reported. This agreement validates the successful synthesis and structural integrity of these compounds.

*(1aR,2R,2aR,5aS,6S,6aS)-4-(4-methoxyphenyl)hexahydro-3H-2,6-epoxyoxireno[2,3-f]isoindole-3,5(4H)-dione (**19**).*

When comparing the infrared spectrum of imide **13** (SM, Figure S50) with the infrared spectrum of epoxide **19** (SM, Figure S74), it is observed that they differ only in the intensity of the band at 1181 cm^−1^, corresponding to the C-O stretching vibration. In compound **19**, there are two C-O bonds present in its structure, which intensifies the signal in the spectrum. The presence of two bands corresponding to the asymmetric and symmetric stretching vibrations of the C=O carbonyl groups of the imide is observed: one band at 1776 cm^−1^ and another at 1706 cm^−1^, with the latter band being more intense. In addition to these bands, there is also a band at 1512 cm^−1^ and another at 1393 cm^−1^ corresponding to the stretching vibration of the C=C bonds in aromatics.

**Figure 2:**
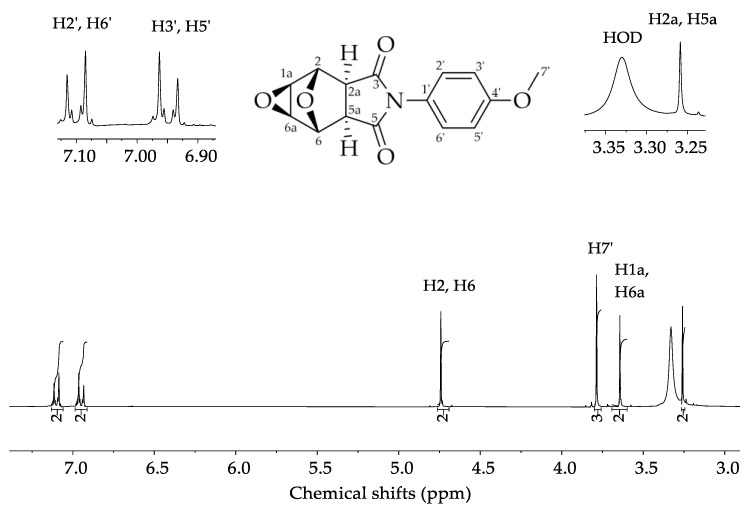
^1^H NMR (300 MHz, DMSO-d_6_, and CDCl_3_) *spectrum* of compound **19**.

In the ^1^H NMR spectrum (Figure 2) we observe the more deshielded signals assigned to the hydrogens bound to the carbon of the aromatic ring, with one signal at *δ* = 7.05–7.19 ppm integrated for two hydrogen atoms (H2’ and H6’), and another signal at *δ* = 6.85–7.00 ppm integrated for the hydrogens H3’ and H5’. Four signals are observed in simple multiplets: *δ* = 4.74 ppm assigned to the hydrogens H2 and H6; *δ* = 3.79 ppm assigned to hydrogen H7’; a characteristic signal of the methyl group hydrogens; *δ* = 3.61 ppm assigned to the hydrogens H1a and H6a; and *δ* = 3.25 ppm assigned to the hydrogens H2a and H5a, the most shielded signal.

**Figure 3:**
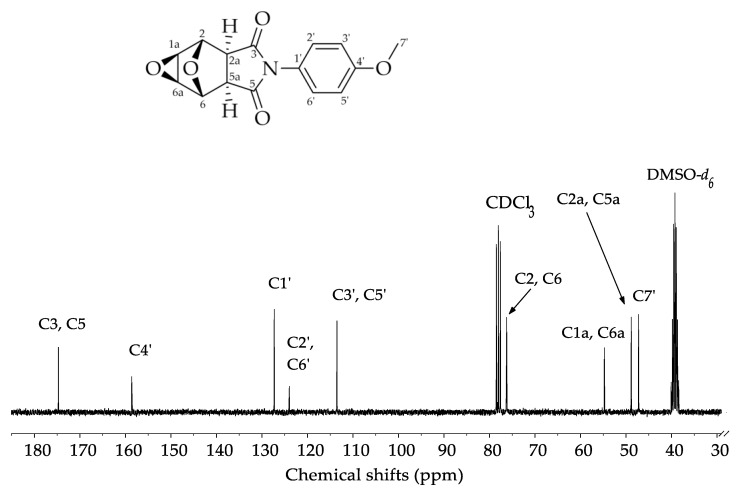
^13^C NMR (75 MHz, DMSO-d_6_, and CDCl_3_) *spectrum* of compound **19**.

In the ^13^C NMR spectrum (Figure 3) there is a signal at *δ* = 175.40 ppm attributed to the carbonyl carbons, C3 and C5. The signal at *δ* = 159.60 ppm was assigned to the olefinic carbon of the aromatic ring, C4’. The signals at *δ* = 128.30 ppm, *δ* = 124.60 ppm, and *δ* = 114.00 ppm are attributed to the other aromatic ring carbons C1’, C2’/C6’, and C3’/C5’, respectively. The signal observed at *δ* = 76.61 ppm was assigned to the carbons C2 and C6, bound to the bridge oxygen atom. The signal at *δ* = 55.80 ppm was assigned to the carbons C1a and C6a. Another signal at *δ* = 49.90 ppm was assigned to the carbons C2a and C5a. Finally, the most shielded signal, at *δ* = 47.90 ppm, was assigned to the methyl group carbon C7’.

### 2.2. Biological Assays

#### 2.2.1. *Bidens pilosa*

**Figure 4:**
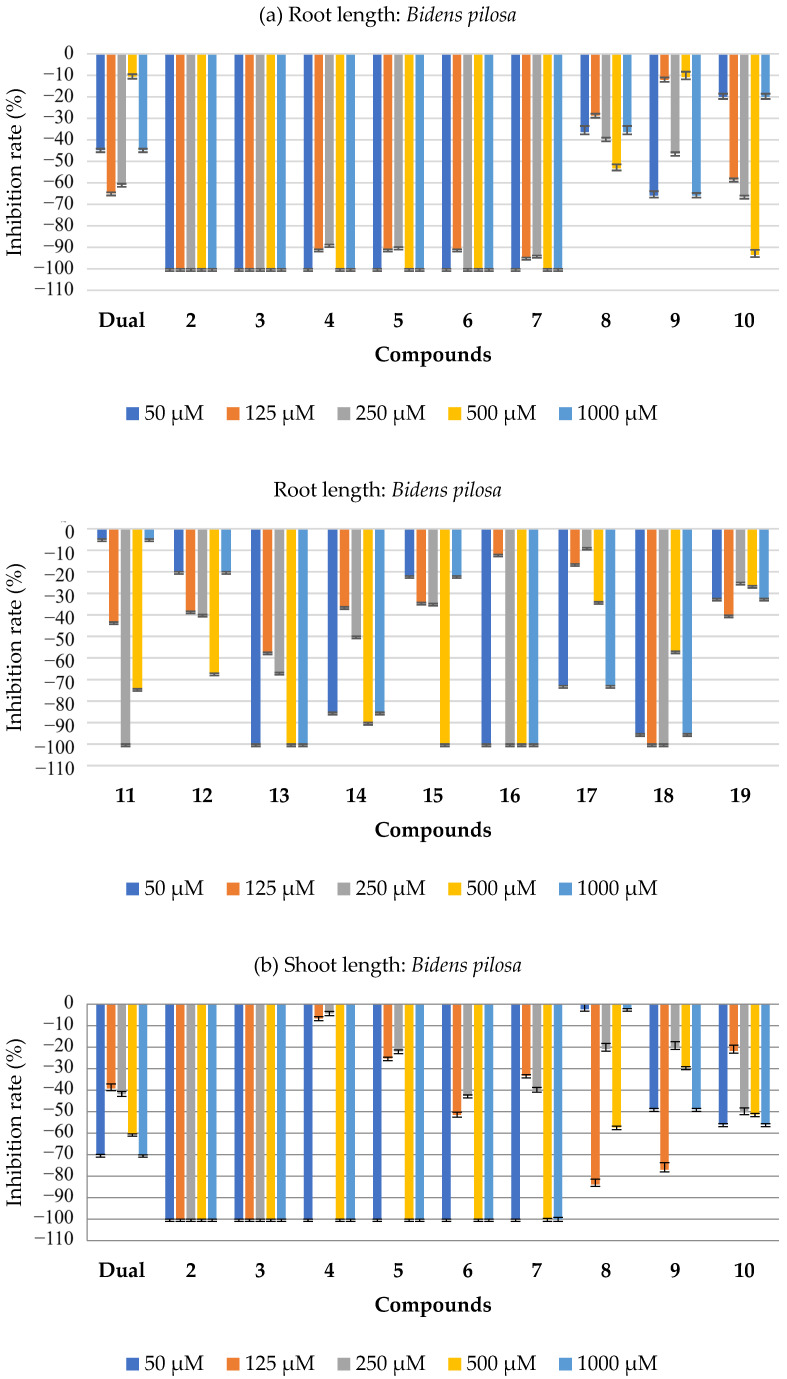

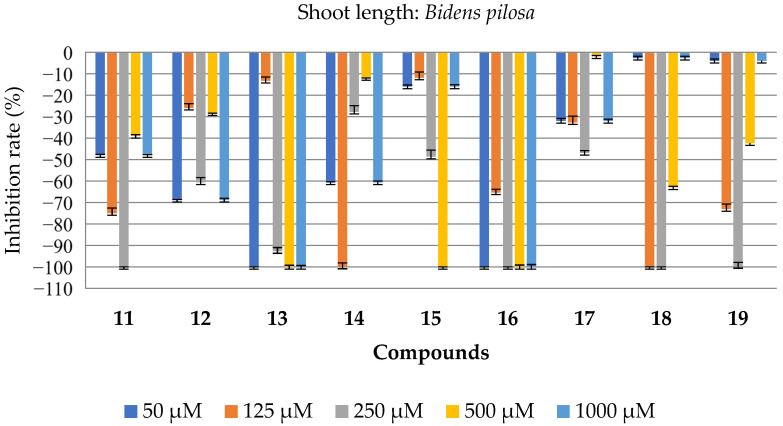
(**a**) Root length and (**b**) shoot length of *Bidens pilosa* in relation to the control solution. Values are expressed as the percentage difference from the control. The error bars represent the standard deviation.

The substances **2**–**19** inhibited the growth of both the aerial and root parts of *B. Pilosa* (Figure 4). Substances **2**–**7**, **13**, and **16** achieved 100% inhibition of both the aerial and root growth at concentrations of 500 and 1000 μM. Compound **11** stood out, at a concentration of 250 μM, by showing 100% inhibition of both the aerial and root parts. Compound **18** showed 100% inhibition of the aerial and root parts at concentrations of 125 and 250 μM. The results are superior to those presented by the commercial herbicide (Dual) at the same concentrations. The tables with all the bioassay results for this species are available in the Supplementary Materials (SM, Tables S1 and S2).

The compounds containing a free carboxylic acid group (specifically compounds 2 to 7) demonstrated the strongest herbicidal effects against the weed *Bidens pilosa* in this study. These molecules were the most effective at inhibiting seed germination and seedling development, including reducing biomass and limiting root and shoot growth. One possible reason for their high activity is that the carboxylic acid group might lower the local pH, which could disrupt seed development processes and contribute to the observed herbicidal effect. 

#### 2.2.2. *Lactuca sativa*

Compounds **2**–**19** demonstrated inhibitory effects on the growth of both the aerial (shoot) and root parts of lettuce seedlings during the bioassays. Notably, compounds **2**–**7**, **16**, and **19** exhibited a significantly higher inhibitory effect on root development compared to the commercial herbicide used as a positive control, when tested at a concentration of 1000 μM. These findings are supported by the data presented in the Supplementary Materials (Figure S77, Tables S3 and S4), which highlight the enhanced phytotoxic activity of these specific compounds, particularly in suppressing root elongation. 

#### 2.2.3. *Allium cepa*

At a concentration of 1000 μM, compounds **3**, **4**, **7**, and **19** stood out by exhibiting higher percentages of inhibition of the aerial parts of lettuce seedlings compared to the commercial herbicide used as a reference. These results suggest that these compounds possess a strong phytotoxic effect on shoot development. In parallel, compounds **2** through **7** were particularly noteworthy for their pronounced inhibitory effects on root growth, all presenting inhibition percentages exceeding 90%, thereby surpassing the efficacy of the commercial herbicide at the same concentration. Among these, compounds **4**, **6**, and **7** were especially effective, achieving complete (100%) inhibition of root development. These observations are detailed in the Supplementary Materials (Figure S78, Tables S5 and S6), reinforcing the potential of these compounds as candidates for herbicidal applications.

#### 2.2.4. *Cucumis sativus*

When evaluating the development of seedlings, it was noted that substances **2**–**19** interfered with growth, both from the aerial and root parts of the cucumbers. Substances **2**–**7** were highlighted for having a rate of inhibition of the root part and the aerial part higher than the rates presented by the commercial herbicide, at all the concentrations tested. Substance **7** stood out with 100% inhibition of the aerial and root parts at all concentrations. Substance **8** stood out with 100% inhibition of the aerial part, at all the tested concentrations. Substance **19** also showed an inhibition rate of the superior root part of the commercial herbicide at concentrations of 500 and 1000 μM (SM, Figure S79, Tables S7 and S8).

#### 2.2.5. *Triticum aestivum*

The synthesized compounds **2**–**19** demonstrated the ability to interfere with the normal development of wheat seedlings, exhibiting varying degrees of phytotoxic activity. Among these, compounds **3**, **5**, **6**, and **7** were particularly notable for their pronounced inhibitory effects on root growth, consistently surpassing the inhibition levels achieved by the commercial herbicide used as a positive control across all concentrations tested. Furthermore, at the concentration of 1000 μM, compounds **5**, **6**, and **7** also showed superior inhibitory activity on the aerial parts (shoots) of the seedlings when compared to the commercial herbicide. These results, detailed in the Supplementary Materials (Figure S80, Tables S9 and S10), highlight the strong potential of these compounds as effective phytotoxic agents with activity comparable to or exceeding that of a standard commercial product.

### 2.3. Molecular Docking

**Figure 5:**
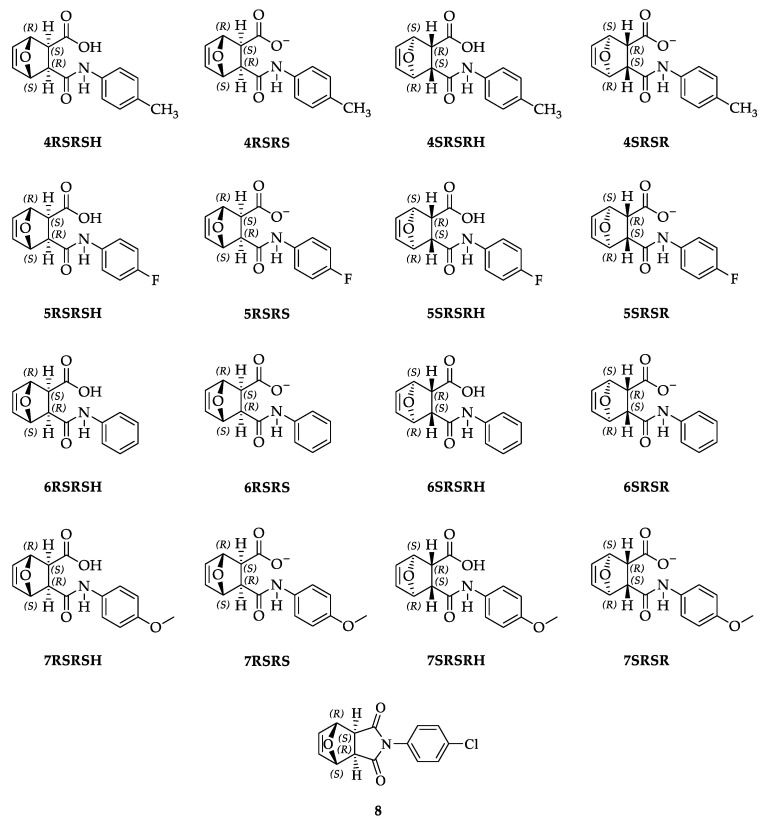
Chemical structures of the most active substances in biological tests.

**Figure 6:**
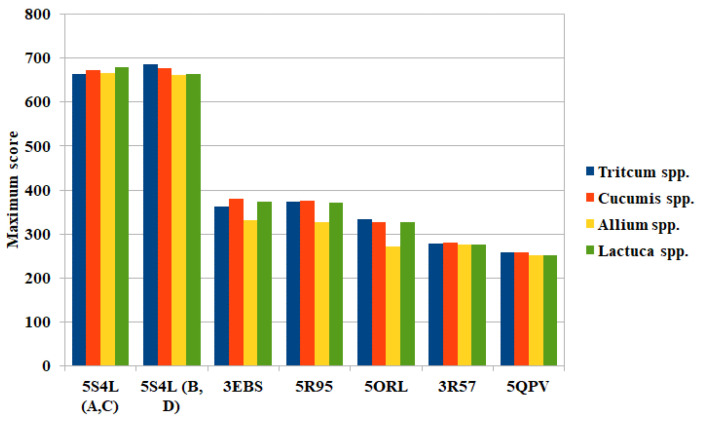
Proteins found in the pharmacophoric search, whose amino acid sequences are most similar to those existing in the genomes of plants of the genera *Allium*, *Cucumis*, *Lactuca*, and *Triticum*.

**Figure 7:**
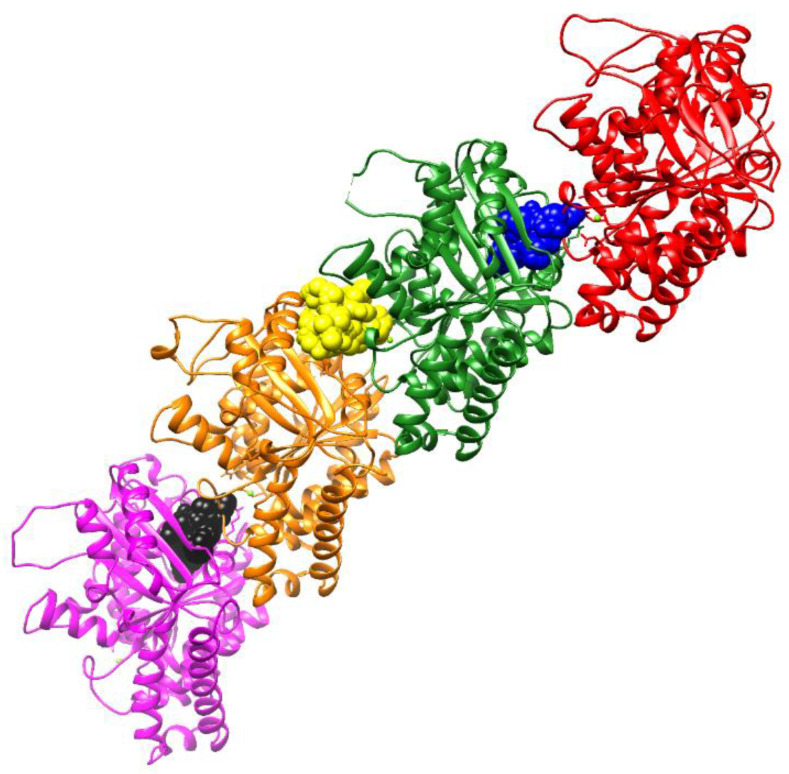
Structure of tubulin 5S4L, with chains A, B, C, and D in red, green, orange, and pink, respectively. Sites B, C, and D correspond to the blue, yellow, and black color regions, respectively.

The pharmacophoric search resulted in the initial selection of the following proteins: 1xnn [26], 2DPZ [27], 2IHQ [28], 2NW4 [29], 2OCU [27], 3DJI [30], 3EBS [31], 3G30 [32], 3R57 [33], 4YJI [34], 5N53 [35], 5ORL [36], 5QGI [27], 5QHU [37], 5QI7 [38], 5QPV [38], 5R95 [39], 5R96 [39], 5REG [40], 5RXQ [41], 5RYN [42], 5RZV [27], 5S3I [43]. 5S4L [44], 5S4V [44], 5S52 [44], 5S5A [44], 5S5R [44], 5SBN [27], 5SMI [42], 6F8B [45], 6FU1 [46], 6FZU [47], 6SOT [39], 6YOX [48], 7B9D [49], 7BCI [49], 7FTD [42], 7NGG [42], 7NLK [42], 7U4J [50], 7VKH [50], 8AOA [51], and 8BTC [52]. Since the ligands of these proteins are similar to compounds **4RSRSH**, **4RSRS**, **4SRSRH**, **4SRSR**, **5RSRSH**, **5RSRS**, **5SRSRH**, **5SRSR**, **6RSRSH**, **6RSRS**, **6SRSRH**, **6SRSR**, **7RSRSH**, **7RSRS**, **7SRSRH**, **7SRSR**, and **8** (Figure 5), it is likely that these compounds can also form complexes with such proteins. However, according to the search made in plant genomes with the amino acid sequences of these proteins, only six proteins were similar to those present in the genome of *Allium* spp., *Cucumis* spp., *Triticum* spp., and *Lactuca* spp. (Figure 6). Among these proteins, the best results were obtained for the chains of tubulin 5S4L [44] (Figure 7), which reached scores above 600.

The tubulin proteins polymerize into long chains or filaments that form microtubules, which are imperative to normal cell function. There are six known tubulins in eukaryotes. In addition, many prokaryotic proteins have been identified as being related to tubulin. *β*-tubulins have been of particular interest due to the potential novel therapeutic approach for cancer treatment [53,54]. In plants, the cortical microtubule array, formed by tubulins, has a primary function in cell growth and elongation, guiding the deposition of new cellulose microfibrils [55]. Consequently, tubulins have been the target of herbicides such as oryzalin (4-(dipropylamino)-3,5-dinitrobenzenesulfonamide [56,57]) and trifluralin (2,6-dinitro-N,N-dipropyl-4-(trifluoromethyl)aniline [58]. There are also flamprop-M-methyl (methyl (2*R*)-2-(*N*-benzoyl-3-chloro-4-fluoroanilino)propanoate [59];) and synthetic cyanoacrylates, such as ethyl (2*Z*)-3-amino-2-cyano-4-ethylhex-2-enoate (CA1; [60,61]).

**Figure 8:**
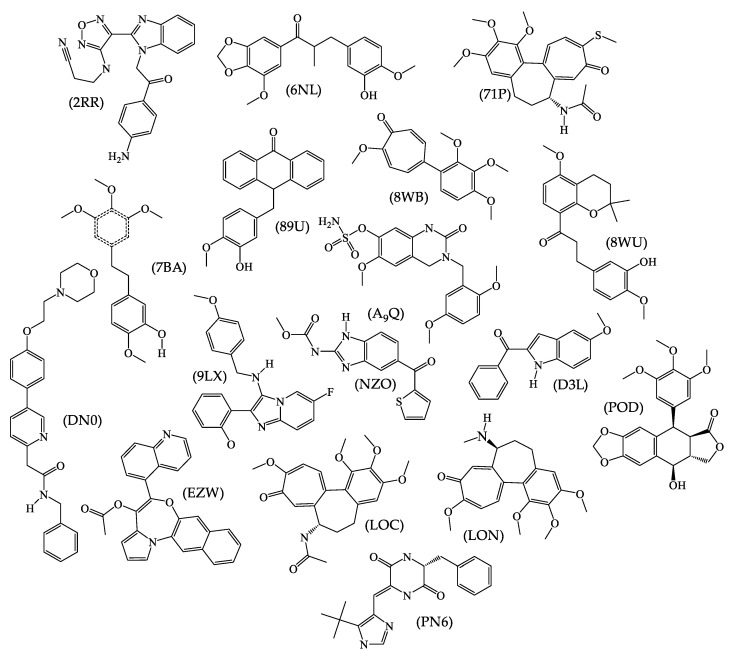
Chemical structures of compounds that inhibit tubulin through complexation to sites B and D of the protein: avanbulin (2RR; [62]), (−)-podophyllotoxin (POD; [63]), colchicine (LOC; [64]), plinabulin (PN6; [65]), nocodazole (NZO; [65]), (E)-3-(3-hydroxy-4-methoxyphenyl)-1-(7-methoxy-1,3-benzodioxol-5-yl)-2-methylprop-2-en-1-one (6NL; [66]), N-[(7R)-1,2,3-trimethoxy-10-methylsulfanyl-9-oxo-6,7-dihydro-5H-benzo[a]heptalen-7-yl]acetamide (71P; [67]), combretastatin A4 (7BA; [68]), 2-methoxy-5-(2,3,4-trimethoxyphenyl)cyclohepta-2,4,6-trien-1-one (8WB; [69]), [3-[(2,5-dimethoxyphenyl)methyl]-6-methoxy-2-oxo-1,4-dihydroquinazolin-7-yl] sulfamate (AQ9; [70]), demecolcine (LON; [71]), 10-[(3-hydroxy-4-methoxyphenyl)methylidene]anthracen-9-one (89U; [72]), (E)-3-(3-hydroxy-4-methoxyphenyl)-1-(5-methoxy-2,2-dimethylchromen-8-yl)prop-2-en-1-one (8WU), 2-[6-fluoro-3-[(4-methoxyphenyl)methylamino]imidazo[1,2-a]pyridin-2-yl]phenol (9LX; [73]), (5-(quinolin-5-yl)naphtho[2,3-b]pyrrolo[1,2-d][1,4]oxazepin-4-yl acetate 8-(EZW; [74]), (5-methoxy-1H-indol-2-yl)-phenylmethanone (D3L; [75]), and tirbanibulin (DN0; [76]).

**Figure 9:**
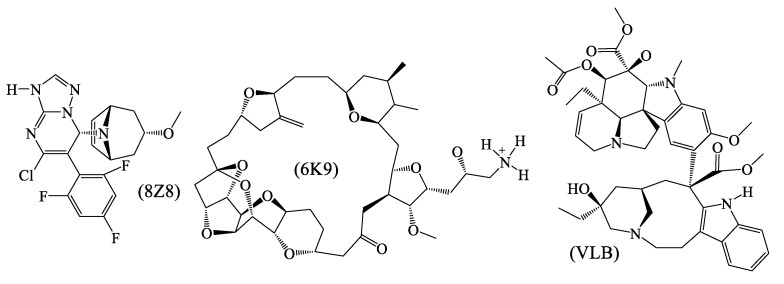
Chemical structures of compounds that inhibit tubulin through complexation to site C of the protein: vinblastine (VLB; [64]), (1R,3R,5S)-8-[(7S)-5-chloro-6-(2,4,6-trifluorophenyl)-3H,7H-[1,2,4]triazolo[1,5-a]pyrimidin-7-yl]-3-methoxy-8-azabicyclo[3.2.1]oct-6-ene (8Z8; [77]), and eribulin (6K9; [78]).

As the compounds **4RSRSH**, **4RSRS**, **4SRSRH**, **4SRSR**, **5RSRSH**, **5RSRS**, **5SRSRH**, **5SRSR**, **6RSRSH**, **6RSRS**, **6SRSRH**, **6SRSR**, **7RSRSH**, **7RSRS**, **7SRSRH**, **7SRSR**, and **8** are structurally similar to the tubulin inhibitors (Figure 8 and Figure 9), it is reasonable to expect that such compounds can also inhibit tubulins. Furthermore, it was observed that the tubulins selected in the pharmacophoric search present amino acid sequences very similar to the tubulin sequences produced by plants of the genera *Allium*, *Cucumis*, *Lactuca*, and *Triticum*, which suggests that inhibitors of the selected tubulins can also inhibit the tubulins produced by plant species of such genera.

**Figure 10:**
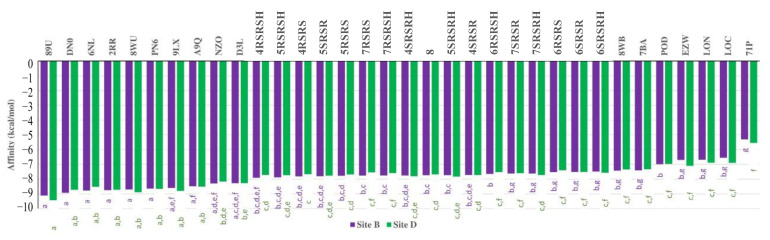
Affinities of the compounds studied in the present work (Scheme 1) and of the tubulin inhibitors (Figure 8) for tubulin sites B and D, according to calculations carried out with the QuickVina 2.1 [79]. Columns with at least one identical letter, for the same protein site, are statistically equal according to the Conover test (*p* < 0.05).

Therefore, it is likely that the above-mentioned compounds can also inhibit tubulins produced by *Allium* spp., *Cucumis* spp., *Lactuca* spp., and *Triticum* spp. Consequently, the tubulins selected in the pharmacophoric search, as well as others structurally similar to them, were used as models to study the interactions of the substances mentioned above with the tubulins produced by species of the mentioned plant genera. It was observed, for sites B and D of tubulins, that the aforementioned compounds present affinity values for the protein that is intermediate in relation to the values calculated for tubulin inhibitors (Figure 10). Therefore, it seems reasonable to suggest that such compounds may in fact be inhibitors of tubulin through the formation of complexes with the B site of such proteins.

**Figure 11:**
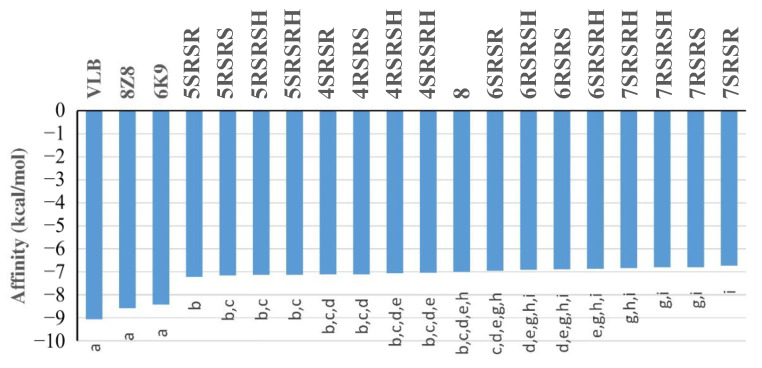
Affinities of the compounds studied in the present work (Scheme 1) and of the tubulin inhibitors (Figure 9) for tubulin site C, according to calculations carried out with the QuickVina 2.1 [79]. Columns with at least one identical letter are statistically equal according to the Conover test (*p* < 0.05).

As for site C, it was observed that the compounds studied always presented values that were statistically higher than those calculated for the inhibitors of tubulins that bind to that site (Figure 11). This result suggests that the compounds studied do not inhibit tubulins through complexation with this site, which seems reasonable, as the inhibitors used present much larger structures than those observed for the compounds studied.

## 3. Materials and Methods

### 3.1. General Experimental Procedures

The reactions were monitored using a thin layer chromatography (TLC) analysis, using silica-gel-coated aluminum plates impregnated with fluorescent indicator F254, which were observed in a chamber under ultraviolet light at 254 nm. The dichloromethane (DCM) used in the reactions was stored in an amber glass vial containing a 4 Å molecular sieve and sealed with film. Nuclear magnetic resonance spectra of hydrogen (^1^H NMR, 300 MHz) and carbon (^13^C NMR, 75 MHz) were obtained on a VARIAN MERCURY 300 MHz spectrometer (Palo Alto, CA, USA), using deuterated dimethyl sulfoxide (DMSO-*d6*) and deuterated chloroform (CDCl_3_) as solvents. The infrared (IR) spectra were obtained on a FT-IR VARIAN 660 spectrometer (Palo Alto, CA, USA) equipped with a GladiATR scan from 4000 to 500 cm^−1^. The melting points are not corrected and have been determined in a MQAPF-302 device (Campinas, São Paulo, Brazil).

### 3.2. Synthetic Procedures

#### 3.2.1. Procedure for the Preparation of **1**

Maleic anhydride (Sigma-Aldrich, St. Louis, MO, USA; 0.5 g, 50 mmol), furan (15 mL, 200 mmol), and anhydrous dichloromethane (20.0 mL) were poured into a 500 mL round-bottom flask. The solution was heated to reflux and kept there for 24 h. After verifying the end of the reaction using a TLC analysis, the resulting mixture was concentrated in a rotary evaporator under vacuum. The solid formed was collected using simple filtration and washed with a solution of hexane and diethyl ether 3:1 (*v*/*v*).

White solid. TLC: R_f_ = 0.48 (hexane/ethyl acetate 2:1 *v*/*v*); mp: 112.4–112.8 °C; 91% yield. IR (ATR, *ῡ*/cm^−1^): 1858, 1784, 1211, 1085, 1018, 951, 732, 632, and 572. ^1^H NMR (300 MHz, DMSO-*d*_6_, *δ* = 3.3 ppm); *δ*: 3.22 (2H, s, H3a, and H7a); 5.26 (2H, t, *J* = 1.0 Hz, H4, and H7); 6.50 (2H, t, *J* = 0.9 Hz, H5, and H6). ^13^C NMR (75 MHz, DMSO-*d*_6_, *δ* = 40.0 ppm); *δ*: 49.20 (C3a and C7a); 81.70 (C4 and C7); 136.90 (C5 and C6); 171.60 (C1 and C3).

#### 3.2.2. Synthesis of Amides **2**–**7**

Compound **1** (500 mg, 3 mmol) and anhydrous DCM (5 mL) were added to a 50 mL round-bottom flask. While stirring, the corresponding amine (3 mmol) was added to the solution. After the reaction was complete, vacuum filtration was used to collect the precipitate, which was then purified by recrystallization from hexane/DCM (3:1, *v*/*v*) to obtain compounds **2**–**7** (Scheme 1).

*rac(1R,2S,3R,4S)-3-((4-chlorophenyl)carbamoyl)-7-oxabicyclo[2.2.1]hept-5-ene-2-carboxylic acid (***2***).* White solid. TLC: R_f_ = 0.3 (hexane/ethyl acetate 1:2 *v*/*v*); mp: 172.8–173.9 °C; 95% yield. IR (ATR, *ῡ*/cm^−1^): 3303, 3195, 3132, 1724, 1668, 1542, 1490, 896, 821, and 498. ^1^H NMR (300 MHz, DMSO-*d*_6_, *δ* = 3.3 ppm); *δ*: 2.65 (1H, d, *J* = 6.0 Hz, H2); 2.76 (1H, d, *J* = 6.0 Hz, H3); 4.99–5.01 (1H, m, and H1); 5.08–5.10 (1H, m, and H4); 6.32–6.52 (2H, m, H5, and H6); 7.21–7.38 (2H, m, H2’, and H6’); 7.47–7.59 (2H, m, H3’, and H5’); 9.86 (1H, s, and OH). ^13^C NMR (75 MHz, DMSO-*d*_6_, *δ* = 40.0 ppm); *δ*: 47.56 (C2); 48.06 (C3); 79.15 (C1); 80.43 (C4); 120.72 (C2’and C6’); 126.55 (C4’); 128.54 (C3’ and C5’); 137.24 (C6); 137.53 (C5); 138.84 (C1’); 169.97 (C3a); 172.65 (C2a).

*rac-(1R,2S,3R,4S)-3-((4-bromophenyl)carbamoyl)-7-oxabicyclo[2.2.1]hept-5-ene-2-carboxylic acid (***3***).* White solid. TLC: R_f_ = 0.3 (hexane/ethyl acetate 1:2 *v*/*v*); mp: 175.2–176.0 °C; 93% yield. IR (ATR, *ῡ*/cm^−1^): 3296, 3188, 3121, 2359, 1720, 1542, 1486, 1074, 918, 892, 818, and 491. ^1^H NMR (300 MHz, DMSO-*d*_6_, *δ* = 3.3 ppm); *δ*: 2.66 (1H, d, *J* = 6.0 Hz, H2); 2.77 (1H, d, *J* = 6.0 Hz, H3); 5.00–5.03 (1H, m, and H1); 5.09–5.12 (1H, m, H4); 6.41–6.52 (2H, m, H5, and H6); 7.40–7.54 (4H, m, H2’, H3’, H5’, and H6’); 9.88 (1H, s, and OH). ^13^C NMR (75 MHz, DMSO-*d*_6_, *δ* = 40.0 ppm); *δ*: 46.84 (C2); 47.47 (C3); 79.14 (C1); 80.39 (C4); 114.51 (C4’); 121.09 (C2’ and C6’); 131,42 (C3’ and C5’); 136.61 (C5); 137.01 (C6); 138.64 (C1’); 169.77 (C3a); 172.61 (C2a).

rac-(1R,2S,3R,4S)-4-(p-tolylcarbamoyl)-7-oxabyciclo[2.2.1]hept-1-ene-5-caboxilic acid (**4**). White solid. TLC: R_f_ = 0.27 (hexane/ethyl acetate 1:2 *v*/*v*). mp: 174.5–175.9 °C; 99% yield. IR (ATR, ῡ/cm^−1^): 3036, 1784, 1211, 1018, 888, and 818. ^1^H NMR (300 MHz, DMSO-d_6_, *δ* = 3.3 ppm); *δ*: 2.22 (3H, s, and H7’); 2.64 (1H, d, *J* = 6.0 Hz, H2); 2.77 (1H, d, *J* = 6.0 Hz, H3); 4.98–5.01 (1H, m, and H1); 5.09–5.12 (1H, m, and H4); 6.36–6.49 (2H, m, H5, and H6); 6.98–7.13 (2H, m, H3’, and H5’); 7.31–7.45 (2H, m, H2’, and H6’); 9.58 (1H, s, and OH). ^13^C NMR (75 MHz, DMSO-d_6_, *δ* = 40.0 ppm); *δ*: 20.18 (C7’); 46.46 (C2); 47.18 (C3); 78.82 (C1); 80.25 (C4); 118.98 (C2’ and C6’); 128.70 (C3’ and C5’); 131.66 (C4’); 136.35 (C6); 136.48 (C5); 13136.68 (C1’), 169.02 (C3a); 172.46 (C2a).

*rac-(1R,2S,3R,4S)-3-((4-fluorophenyl)carbamoyl)-7-oxabyciclo[2.2.1]hept-5-ene-2-caboxilic acid (***5***).* White solid. TLC: R_f_ = 0.23 (hexane/ethyl acetate 1:2 *v*/*v*). mp: 175.8–176.9 °C; 99% yield. IR (ATR, *ῡ*/cm^−1^): 3303, 3221, 3158, 3099, 1720, 1679, 1561, 1505, 1215, 896, 836, 516, and 476. ^1^H NMR (300 MHz, DMSO-*d*_6_, *δ* = 3.3 ppm); *δ*: 2.54–2.65 (1H, m, and H2); 2.74 (1H, d, *J* = 9.1 Hz, H3); 4.95–5.01 (1H, m, and H1); 5.07–5.10 (1H, m, and H4); 6.39–6.46 (2H, m, H5, and H6); 6.99–7.15 (2H, m, H3’, and H5’); 7.39–7.56 (2H, m, H2’, and H6’); 9.73 (1H, s, OH). ^13^C NMR (75 MHz, DMSO-*d*_6_, *δ* = 40.0 ppm); *δ*: 46.80 (C2); 47.38 (C3); 79.12 (C4); 80.43 (C1); 115.01 (C3’); 115.30 (C5’); 121.02 (C2’and C6’); 135.67 (C1’); 136.63 (C5); 136.99 (C6); 159.50 (C4’); 169.50 (C3a); 172.69 (C2a).

*rac-(1R,2S,3R,4S)-3-(phenylcarbamoyl)-7-oxabyciclo[2.2.1]hept-5-ene-2-caboxilic acid (***6***).* White solid. TLC: R_f_ = 0.20 (hexane/ethyl acetate 1:2 *v*/*v*). mp: 176.8–177.9 °C; 99% yield. IR (ATR, *ῡ*/cm^−1^): 3303, 3203, 2946, 1720, 1679, 1594, 1546, 1442, 910, 888, 751, and 487. ^1^H NMR (300 MHz, DMSO-*d*_6_, *δ* = 3.3 ppm); *δ*: 2.61 (1H, d, *J* = 6.0 Hz, H2); 2.73 (1H, d, *J* = 6.0 Hz, H3); 4.94–4.98 (1H, m, and H1); 5.04–5.07 (1H, m, and H4); 6.36–6.41 (2H, m, H5, and H6); 6.85–7.07 (1H, m, and H4’); 7.13–7.29 (2H, m, H3’, and H5’); 7.35–7.56 (2H, m, H2’, and H6’); 9.63 (1H, s, and OH). ^13^C NMR (75 MHz, DMSO-*d*_6_, *δ* = 40.0 ppm); *δ*: 47.21 (C2); 47.91 (C3); 79.54 (C4); 80.92 (C1); 119.61 (C2’ and C6’); 123.46 (C4’); 129.04 (C3’ and C5’); 136.70 (C6); 136.98 (C5); 139.67 (C1’); 169.97 (C3a); 173.15 (C2a).

*rac-(1R,2S,3R,4S)-3-((4-methoxyphenyl)carbamoyl)-7-oxabyciclo[2.2.1]hept-5-ene-2-caboxilic acid (***7***).* White solid. TLC: R_f_ = 0.23 (hexane/ethyl acetate 1:2 *v*/*v*). mp: 142.6–143.8 °C; 92% yield. IR (ATR, *ῡ*/cm^−1^): 3310, 2824, 1720, 1653, 1553, 1508, 1368, 1029, 910, 832, and 491. ^1^H NMR (300 MHz, DMSO-*d*_6_); *δ*: 2.60 (1H, d, *J* = 6.0 Hz and H2); 2.71 (1H, d, *J* = 6.0 Hz, H3); 3.65 (3H, s, and H7’); 4.95–4.97 (1H, m, and H1); 5.06–5.08 (1H, m, and H4); 6.39–6.44 (2H, m, H5, and H6); 6.75–6.94 (2H, m, H3’, and H5’); 7.27–7.44 (2H, m, H2’, and H6’); 9.50 (1H, s, and OH). ^13^C NMR (75 MHz, DMSO-*d*_6_, *δ* = 40.0 ppm); *δ*: 46.72 (C2); 47.37 (C3); 55.22 (C7’); 79.09 (C4); 80.53 (C1); 113.78 (C3’ and C5’); 120.85 (C2’ and C6’); 132.45 (C5 and C6); 136.67 (C1’); 155.16 (C4’); 169.09 (C3a); 172.80 (C2a).

#### 3.2.3. Synthesis of Imides **8**–**13**

In a 50 mL round-bottom flask, compounds **2**–**7** (1 mmol) were dissolved in methanol (20 mL), and concentrated sulfuric acid (1 mL) was then added to the resulting solution. After stirring for 30 min, the end of the reaction was observed using a TLC analysis. The excess methanol was removed on a rotary evaporator to afford a precipitate that was dissolved in anihydrous dichloromethane. The resulting solution was extracted with sodium bicarbonate solution. The aqueous phase was discarded while the organic phase was concentrated on a rotary evaporator. The residue formed was washed with hexane and dichloromethane 3:1 (*v*/*v*) to obtain the corresponding imide **8**–**13** (Scheme 1).

*(3aR,4S,7R,7aS)-2-(4-chlorophenyl)-3a,4,7,7a-tetrahydro-1H-4,7-epoxyisoindole-1,3(2H)-dione (***8***).* White solid. TLC: R_f_ = 0.37 (hexane/ethyl acetate 2:1 *v*/*v*). mp: 173.0–173.9 °C; 78% yield. IR (ATR, *ῡ*/cm^−1^): 1776, 1702, 1486, 1375, 1181, 1089, 1011, 873, 721, 646, and 513. ^1^H NMR (300 MHz, CDCl_3_, *δ* = 7.26 ppm); *δ*: 3.00 (2H, s, H3a, and H7a); 5.38 (2H, t, *J* = 1 Hz, H4, and H7); 6.56 (2H, t, *J* = 1.0 Hz, H5, and H6); 7.17–7.31 (2H, m, H2’, and H6’); 7.34–7.50 (2H, m, H3’, and H5’). ^13^C NMR (75 MHz, CDCl_3_, *δ*_CDCl3_ = 77.0 ppm): *δ*: 47.68 (C3a and C7a); 81.57 (C4 and C7); 127.93 (C2’ and C6’); 129.48 (C3’ and C5’); 130.28 (C1’); 134.71 (C4); 136.85 (C5 and C6); 175.20 (C1 and C3).

*(3aR,4S,7R,7aS)-2-(4-bromophenyl)-3a,4,7,7a-tetrahydro-1H-4,7-epoxyisoindole-1,3(2H)-dione (***9***).* White solid. TLC: R_f_ = 0.34 (hexane/ethyl acetate 2:1 *v*/*v*). mp: 142.2–143.1 °C; 76% yield. IR (ATR, *ῡ*/cm^−1^): 1776, 1702, 1483, 1375, 1181, 1011, 873, 803, 721, and 647. ^1^H NMR (300 MHz, CDCl_3_, *δ* = 7.26 ppm); *δ*: 3.00 (2H, d, *J* = 0.7, H3a and H7a); 5.38 (2H, t, *J* = 1.0 Hz, H4 e, and H7); 6.57 (2H, t, *J* = 1 Hz, H5, and H6); 7.05–7.24 (2H, m, H3’, and H5’); 7.49–7.68 (2H, m, H2’, and H6’). ^13^C NMR (75 MHz, CDCl_3_, *δ_CDCl3_* = 77.0 ppm); *δ*: 48.13 (C3a and C7a); 82.01 (C4 and C7); 123.22 (C4’); 128.64 (C2’ and C6’); 131.20 (C1’); 132.90 (C3’ and C5’); 137.29 (C5 and C6); 175.57 (C1 and C3).

*(3aR,4S,7R,7aS)-2-(p-tolyl)-3a,4,7,7a-tetrahydro-1H-4,7-epoxyisoindole-1,3(2H)-dione (***10***).* White solid. TLC: R_f_ = 0.35 (hexane/ethyl acetate 2:1 *v*/*v*). mp: 132.1–133.3 °C; 60% yield. IR (ATR, *ῡ*/cm^−1^): 1776, 1706, 1512, 1382, 1185, 1014, 873, 803, 706, 639, and 513. ^1^H NMR (300 MHz, CDCl_3_, *δ* = 7.26 ppm); *δ*: 2.37 (3H, s, and H7’); 2.99 (2H, s, H3a, and H7a); 5.38 (2H, t, *J* = 1.0 Hz, H4, and H7); 6.55 (2H, t, *J* = 1.0 Hz, H5, and H6); 7.10–7.19 (2H, m, H3’, and H5’); 7.21–7.31 (2H, m, H2’, and H6’). ^13^C NMR (75 MHz, CDCl_3_, *δ* = 77.0 ppm); *δ*: 21.21 (C7’), 47.52 (C3a and C7a); 81.38 (C4 and C7); 126.34 (C2’ and C6’); 129.03 (C3’ and C5’); 129.82 (C1’); 136.68 (C4’); 138.90 (C5 and C6); 175.53 (C1 and C3).

*(3aR,4S,7R,7aS)-2-(4-fluorophenyl)-3a,4,7,7a-tetrahydro-1H-4,7-epoxyisoindole-1,3(2H)-dione (***11***).* White solid. TLC: R_f_ = 0.37 (hexane/ethyl acetate 2:1 *v*/*v*). mp: 153.9–154.4 °C; 80% yield. IR (ATR, *ῡ*/cm^−1^): 1776, 1706, 1508, 1386, 1226, 1185, 1011, 873, 717, 643, and 479. ^1^H NMR (300 MHz, CDCl_3_, *δ* = 7.26 ppm); *δ*: 3.00 (2H, s, H3a, and H7a); 5.38 (2H, t, *J* = 1.0 Hz, H4, and H7); 6.56 (2H, t, *J* = 1.0 Hz, H5, and H6); 7.08–7.20 (2H, m, H3’, and H5’); 7.22–7.34 (2H, m, H2’, and H6’). ^13^C NMR (75 MHz, CDCl_3_, *δ* = 77.0 ppm); *δ*: 47.50 (C3a and C7a); 81.42 (C4 and C7); 116.02 (C3’); 116.33 (C5’); 128.37 (C2’ and C6’); 128.49 (C1’); 136.70 (C5 and C6); 163.94 (C4’); 175.29 (C1 and C3).

*(3aR,4S,7R,7aS)-2-phenyl-3a,4,7,7a-tetrahydro-1H-4,7-epoxyisoindole-1,3(2H)-dione (***12***).* White solid. TLC: R_f_ = 0.53 (hexane/ethyl acetate 1:1 *v*/*v*). mp: 171.8–172.9 °C; 71% yield. IR (ATR, *ῡ*/cm^−1^): 1776, 1706, 1490, 1375, 1185, 1141, 1014, 873, 773, 710, 650, and 487. ^1^H NMR (300 MHz, CDCl_3_, *δ* = 7.26 ppm); *δ*: 3.00 (2H, s, H3a, and H7a); 5.39 (2H, t, *J* = 1.0 Hz, H4, and H7); 6.55 (2H, t, *J* = 1.0 Hz, H5, and H6); 7.19–7.31 (2H, m, H2’, and H6’); 7.31–7.57 (3H, m, H3’, H4’, and H5’). ^13^C NMR (75 MHz, CDCl_3_, *δ* = 77.0 ppm); *δ*: 47.66 (C3a and C7a); 81.53 (C4 and C7); 126.69 (C2’ and C6’); 128.91 (C3’ and C5’); 129.28 (C4’); 131.80 (C1’); 136.82 (C5 and C6); 175.50 (C1 and C3).

*(3aR,4S,7R,7aS)-2-(4-methoxyphenyl)-3a,4,7,7a-tetrahydro-1H-4,7-epoxyisoindole-1,3(2H)-dione (***13***).* White solid. TLC: R_f_ = 0.53 (hexane/ethyl acetate 1:1 *v*/*v*). mp: 155.9–156.5 °C; 86% yield. IR (ATR, *ῡ*/cm^−1^): 2954, 2835, 1776, 1706, 1512, 1390, 1248, 1185, 1014, 873, 806, 702, 643, and 591. ^1^H NMR (300 MHz, CDCl_3_, *δ* = 7.26 ppm); *δ*: 2.98 (2H, s, H3a, and H7a); 3.81 (3H, s, and H7’); 5.37 (2H, t, *J* = 1.0 Hz, H4, and H7); 6.55 (2H, t, *J* = 1.0 Hz, H5, and H6); 6.92–7.00 (2H, m, H3’, and H5’); 7.14–7.22 (2H, m, H2’, and H6’). ^13^C NMR (75 MHz, CDCl_3_, *δ* = 77.0 ppm); *δ*: 48.00 (C3a and C7a); 56.02 (C7’); 81.83 (C4 and C7); 115.03 (C3’ and C5’); 124.87 (C2’ and C6’); 128.34 (C1’); 137.22 (C5 and C6); 160.19 (C4’); 176.21 (C1 and C3).

#### 3.2.4. Synthesis of Epoxides **14**–**17**, and **19**

In a 50 mL round-bottom flask, imides **7**–**10**, and **12** (1 mmol) were dissolved in 20 mL of chloroform. The resulting solution was heated to reflux and 77% metachloroperbenzoic acid (*m*-CPBA) (4 mmol) was added in small portions. The reaction was kept under reflux and stirring for 5h. The end of the reaction was observed using a TLC analysis. The solvent was removed on a rotary evaporator and the residue formed was washed with diethyl ether to obtain the corresponding epoxy **14**–**17** and **19**) (Scheme 1).

*(1aR,2R,2aR,5aS,6S,6aS)-4-(4-chlorophenyl)hexahydro-3H-2,6-epoxyoxireno[2,3-f]isoindole-3,5(4H)-dione (***14***).* White solid. TLC: R_f_ = 0.52 (hexane/ethyl acetate 1:2 *v*/*v*). mp: 259.9–260.9 °C; 75% yield. IR (ATR, *ῡ*/cm^−1^): 3099, 2976, 1776, 1706, 1490, 1375, 1267, 1185, 1089, 1014, 862, 832, 732, 628, and 513. ^1^H NMR (300 MHz; DMSO-d_6_; *δ* = 3.3 ppm); *δ*: 3.24 (2H, s, H2a, and H5a); 3.65 (2H, s, H1a, and H6a); 4.68 (2H, s, H2, and H6); 7.04–7.20 (2H, m, H2’, and H6’); 7.32–7.51 (2H, m, H3’, and H5’). ^13^C NMR (75 MHz; DMSO-d_6_; *δ* = 40.0 ppm); *δ*: 48.01 (C2a and C5a); 49.35 (C1a and C6a); 76.61 (C2 and C6); 128.68 (C2’ and C6’); 129.17 (C3’ and C5’); 130.94 (C1’); 133.28 (C4’); 175.40 (C3 and C5).

*(1aR,2R,2aR,5aS,6S,6aS)-4-(4-bromophenyl)hexahydro-3H-2,6-epoxyoxireno[2,3-f]isoindole-3,5(4H)-dione (***15***).* White solid. TLC: R_f_ = 0.55 (hexane/ethyl acetate 1:2 *v*/*v*). mp: 275.5–276.4 °C; 68% yield. IR (ATR, *ῡ*/cm^−1^): 3095, 2987, 1776, 1706, 1486, 1379, 1185, 1014, 832, and 628. ^1^H NMR (300 MHz, DMSO-*d*_6_, and CDCl_3_); *δ*: 3.20 (2H, s, H2a, and H5a); 3.63 (2H, s, H1a, and H6a); 4.71 (2H, s, H2, and H6); 7.07–7.30 (2H, m, H2’, and H6’); 7.36–7.48 (1H, m, and H3’); 7.50–7.65 (1H, m, and H5’). ^13^C NMR (75 MHz, DMSO-*d*_6_, and CDCl_3_); *δ*: 47.98 (C2a and C5a); 49.39 (C1a and C6a); 76.80 (C2 and C6); 121.69 (C4’); 128.28 (C2’); 128.99 (C6’); 130.96 (C3’); 131.39 (C5’); 131.93 (C1’); 175.00 (C3 and C5).

*(1aR,2R,2aR,5aS,6S,6aS)-4-(p-tolyl)hexahydro-3H-2,6-epoxyoxireno[2,3-f]isoindole-3,5(4H)-dione (***16***).* White solid. TLC: R_f_ = 0.50 (hexane/ethyl acetate 1:2 *v*/*v*). mp: 239.8–240.9 °C; 62% yield. IR (ATR, *ῡ*/cm^−1^): 3039, 1776, 1706, 1512, 1375, 1185, 1018, 862, 799, 673, 624, and 498. ^1^H NMR (300 MHz, CDCl_3_, *δ* = 7.26 ppm); *δ*: 2.36 (3H, s, and H7’); 3.11 (2H, s, H2a, and H5a); 3.52 (2H, s, H1a, and H6a); 4.92 (2H, s, H2, and H6); 7.05–7.15 (2H, m, H3’, and H5’); 7.19–7.31 (2H, m, H2’, and H6’). ^13^C NMR (75 MHz, CDCl_3_, *δ_CDCl3_* = 77.0 ppm); *δ*: 20.88 (C7’); 47.55 (C2a and C5a); 49.41 (C1a and C6a); 58.05 (C2 and C6); 125.97 (C2’ and C6’); 129.58 (C3’ and C5’); 129.70 (C1’); 138.87 (C4’); 174.41 (C3 and C5).

*(1aR,2R,2aR,5aS,6S,6aS)-4-(4-fluorophenyl)hexahydro-3H-2,6-epoxyoxireno[2,3-f]isoindole-3,5(4H)-dione (***17***).* White solid. TLC: R_f_ = 0.43 (hexane/ethyl acetate 1:2 *v*/*v*). mp: 244.7–245.6 °C; 75% yield. IR (ATR, *ῡ*/cm^−1^): 2983, 1776, 1706, 1505, 1386, 1181, 1018, 866, 795, 673, 624, and 498. ^1^H NMR (300 MHz, DMSO-*d_6_*, and CDCl_3_); *δ*: 3.25 (2H, s, H2a, and H5a); 3.68 (2H, s, H1a, and H6a); 4.75 (2H, s, H2, and H6); 7.18–7.23 (4H, m, H2’, H3’, H5’, and H6’). ^13^C NMR (75 MHz, DMSO-*d*_6_, and CDCl_3_); *δ*: 47.97 (C2a and C5a); 49.43 (C1a and C6a); 76.80 (C2 and C6); 115.73 (C3’); 116.03 (C5’); 128.88 (C2’); 128, 98 (C6’); 160.05 (C1’); 163.31 (C4’); 175.28 (C3 and C5).

*(1aR,2R,2aR,5aS,6S,6aS)-4-(4-methoxyphenyl)hexahydro-3H-2,6-epoxyoxireno[2,3-f]isoindole-3,5(4H)-dione (***19***).* White solid. TLC: R_f_ = 0.33 (hexane/ethyl acetate 1:2 *v*/*v*). mp: 251.9–252.4 °C; 98% yield. IR (ATR, *ῡ*/cm^−1^): 2961, 1776, 1706, 1512, 1393, 1248, 1181, 1018, 862, 799, 762, 624, and 516. ^1^H NMR (300 MHz, DMSO-*d_6_*, and CDCl_3_); *δ:* 3.25 (2H, s, H2a, and H5a); 3.61 (2H, s, H1a, and H6a); 3.79 (1H, s, and H7’); 4.74 (2H, s, H2, and H6); 6.85–7.00 (2H, m, H3’, and H5’); 7.05–7.19 (2H, m, H2’, and H6’). ^13^C NMR (75 MHz, DMSO-*d_6_*, and CDCl_3_); *δ*: 47.9 (C7’); 49.9 (C2a and C5a); 55.8 (C1a and C6a); 76.6 (C2 and C6); 114.0 (C3’ and C5’); 124.6 (C2’ and C6’); 128.3 (C1’); 159.6 (C4’); 175.4 (C3 and C5).

#### 3.2.5. Synthesis of Epoxy **18**

In a 50 mL round-bottom flask, the previously synthesized imide **12** (1 mmol) was dissolved in 20 mL of DCM. Then, 77% *m*-CPBA (4 mmol) was added in small portions. The mixture was stirred at room temperature for 26 h. The end of the reaction was observed using a TLC analysis. After that, the solvent was removed on a rotary evaporator and the residue was washed with diethyl ether to obtain compound **18** (Scheme 1).

*(1aR,2R,2aR,5aS,6S,6aS)-4-phenylhexahydro-3H-2,6-epoxyoxireno[2,3-f]isoindole-3,5(4H)-dione (***18***).* White solid. TLC: R_f_ = 0.48 (hexane/ethyl acetate 1:2 *v*/*v*). mp: 248.5–250.1 °C; 75% yield. IR (ATR, *ῡ*/cm^−1^): 3073, 1776, 1706, 1490, 1375, 1181, 1018, 992, 855, 762, 695, 606, and 513. ^1^H NMR (300 MHz, DMSO-*d_6_*, and CDCl_3_); *δ*: 3.25 (2H, s, H2a, and H5a); 3.63 (2H, s, H1a, and H6a); 4.72 (2H, s, H2, and H6); 7.08–7.24 (2H, m, H2’, and H6’); 7.27–7.48 (3H, m, H3’, H4’, and H5’). ^13^C NMR (75 MHz, DMSO-*d_6_*, and CDCl_3_); *δ*: 48.20 (C2a and C5a); 49.71 (C1a and C6a); 77.12 (C2 and C6); 126.99 (C2’ and C6’); 127.01 (C3’ and C5’); 132.35 (C4’); 136.85 (C1’); 175.47 (C3 and C5).

### 3.3. Experimental Procedure for the Bioassay

The stock solution (1000 μM) for each test substance was prepared in an aqueous solution of DMSO 0.3% *v*/*v* (Sigma-Aldrich). Solutions at other concentrations (500, 250, 125, and 50 μM) were obtained through dilutions of the stock solutions. The aliquots (5 mL) of all solutions were poured into 90 mm diameter Petri dishes containing germination paper (Whatman n.1) and 20 seeds of *L. sativa* spp., *A. cepa* spp., *C. sativus* spp., *B. Pilosa*, or *T. aestivum* per plate. The lettuce seeds (*L. sativa* spp.) are of the *Vitória Santo Antão Lettuce* cultivar, from the Topseed Garden line by Topseed, batch: 077732, with 85% germination and 99% purity. The onion seeds (*A. cepa* spp.) are of the *Baia Periforme Onion* cultivar, from the Topseed Garden line by Topseed, batch: 049969, with 85% germination and 99% purity. The cucumber seeds (*C. sativus* spp.) are of the traditional cucumber cultivar, from Topseed, batch: 058807, with 85% germination and 99.9% purity. The *Bidens pilosa* seeds were collected from the experimental field at the Agronomy Department of the Federal University of Viçosa, Viçosa, Minas Gerais, Brazil, with 95% germination. The wheat seeds *T. aestivum*, cultivar *ORS Soberano*, were provided by the Wheat Department of the Federal University of Viçosa, Minas Gerais, Brazil, with 90% germination. Subsequently, the dishes were sealed with plastic film and placed in a germination chamber (B.O.D.) at 25 °C, in the absence of light, for 120 h. After this period, the dishes were stored at −10 °C for 24 h to stop the biological process and to precisely measure the stretching of the root. All treatments were replicated three times in an entirely randomized delineation. Aqueous 0.3% (*v*/*v*) DMSO was used as a negative control. The commercial herbicide Dual (Dual Gold from Syngenta, Paulínia, SP, Brazil) was used as a positive control, at the same concentrations as the synthesized compound solutions (1000, 500, 250, 125, and 50 μM). Mix 80–100% of 2-chloro-*N*-(2-ethyl-6-methylphenyl)-*N*-[(2*S*)-1-methoxypropan-2-yl]acetamide and 0–20% 2-chloro-*N*-(2-ethyl-6-methylphenyl)-*N*-[(2*S*)-1-methoxypropan-2-yl]acetamide (*S*-metolachloro).

### 3.4. Procedure for Molecular Docking

#### 3.4.1. Pharmacophoric Search

The chemical structures of substances **4**, **5**, **6**, **7**, and **8**, selected for presenting the best results in the bioassays, were drawn using the computer program MarvinSketch 19.21 (ChemAxon; https://www.chemaxon.com). Both enantiomers were drawn for each substance. In addition, structures containing protonated and deprotonated carboxyls were considered (Figure 5).

The computational programs ACD ChemSketch 12.0121 (Advanced Chemical Development 2012), OpenBabel 3.0.0 [80], Open3Dalign 2.3 [81], and MOPAC 22.1.1 [82] were used as described in the literature [83] to obtain the most stable conformations of compounds **4**–**8**, which underwent a pharmacophoric search using Lisica 1.0.1 [84] and the Ligand Expo database [85]. All proteins whose ligands had Tanimoto scores ≥ 0.5 in the pharmacophoric search and were not chemically bound to the proteins were selected for the next step.

#### 3.4.2. Docking

The three-dimensional structures of tubulin 5S4L [44], and all tubulins with similarities of amino acid sequences equal to or greater than 90% in relation to the amino acid sequence of 5S4L, were obtained from the RCSB Protein Data Bank [86]. The three-dimensional structures underwent the removal of alternation locations with VMD 1.9.3 [87] and alignment with Lovoalign 21.027 [88]. All those structures complexed to a tubulin inhibitor, with up to 3.0 Å of root-mean-square deviation of atomic positions (RMSD) in relation to the tubulin 5S4L, with no missing atoms in the binding sites or close to them, and no amino acid mutations were selected for the docking step. The selected tubulin complexes were subjected to the addition of hydrogen atoms with the UCSF Chimera 1.17.3 computational program [89], which eliminated all components of the complexes that did not correspond to tubulin, guanosine-5’-triphosphate (GTP), guanosine-5’-diphosphate (GDP), and magnesium ion (Mg^2+^). Then, the hydrogenated structures were converted to a pdbqt format using the computer program MGLtools 1.5.7rc1 [90]. Tubulin inhibitors (Figure 8 and Figure 9) were drawn and underwent a pka calculation using the program Marvin Sketch 19.21 (ChemAxon; https://www.chemaxon.com). The most stable conformation of each compound was obtained as described above, to be converted to the pdbqt format using MGLTools 1.5.7rc1 [90].

The most stable conformations of tubulin inhibitors and compounds **4RSRSH**, **4RSRS**, **4SRSRH**, **4SRSR**, **5RSRSH**, **5RSRS**, **5SRSRH**, **5SRSR**, **6RSRSH**, **6RSRS**, **6SRSRH**, **6SRSR**, **7RSRSH**, **7RSRS**, **7SRSRH**, **7SRSR**, and **8**, in the pdbqt format, were docked to tubulins 5S4L [44], 5S4V [44], 5S52 [44], 5S5A [44], 5S5R [44], 1SA1 [63], 3UT5 [64], 4EB6 [64], 4O2A [62], 4O2B [62], 4X1L [91], 4X1K [91], 4X1Y [91], 4X20 [91], 5BMV, 5C8Y [65], 5CA1 [65], 5J2T [92], 5JH7 [78], 5JVD [66], 5LP6 [67], 5LXS [93], 5LXT [93], 5LYJ [68], 5NFZ [69], 5NG1 [69], 5NJH [77], 5OSK [70], 5XI5 [94], 5XIW [95], 5XKE [94], 5XLZ [72], 5YL2 [95], 5ZXH [73], 6GJ4 [74], 6K9V [75], and 6KNZ [76] using the computational program QuickVina 2.1 [79]. Except for the exhaustiveness, which was set to 256, all parameters remained with the default values. The docking was directed to cobblestone-shaped regions (B, C, and D; Figure 7) corresponding to the size of tubulin inhibitors in their experimental complexes with the protein (1SA1, 3UT5, 4EB6, 4O2A, 4O2B, 4X1I, 4X1K, 4X1Y, 4X20, 5BMV, 5C8Y, 5CA1, 5J2T, 5JH7, 5JVD, 5LP6, 5LYJ, 5NFZ, 5NG1, 5NJH, 5OSK, 5XI5, 5XIW, 5XKE, 5XLZ, 5YL2, 5ZXH, 6GJ4, 6K9V, and6KNZ), plus 10 Angstrons on all three Cartesian axes.

#### 3.4.3. Statistical Calculation

The affinities calculated with the QuickVina 2.1 program were subjected to the Shapiro–Wilk normality test [96] and Bartlett homoscedasticity test [97]. As neither normality (*p* < 0.05) nor homogeneity of variances (*p* < 0.05) was observed, the Kruskal–Wallis test was performed [98], which resulted in *p* < 0.00001. Consequently, treatments were compared through the Conover test at 5% significance, using the single-step method to adjust *p*-values [99]. All statistical calculations were performed using the computer program R 4.4.0. Specifically, to perform the Conover test, the R package PMCMRplus was used [100].

## 4. Conclusions

In conclusion, the synthesized compounds were obtained through simple synthetic routes and exhibited yields ranging from 60% to 99%. The conversion of maleic anhydride into nitrogenated terpenoid derivatives produced compounds with significant phytotoxic activity. Epoxy lactones **2**, **3**, **4**, **5**, **6**, and **7** showed promising results, especially against *B. pilosa*, exhibiting 100% inhibition of seedling growth at concentrations of 500 and 1000 µM. These results are superior to those of the commercial herbicide Dual at the same concentrations. Additionally, an in silico analysis identified plant tubulin, an essential protein in the plant cell cytoskeleton, as a possible enzymatic target of compounds **4**–**7**. These findings suggest that terpenoid derivatives may serve as a promising structural platform in the search for new compounds with phytotoxic properties.

## Data Availability

The original contributions presented in this study are included in the article/Supplementary Material. Further inquiries can be directed to the corresponding author.

## Supplementary Materials

The following supporting information can be downloaded at: https://www.mdpi.com/article/10.3390/plants14131933/s1.

## References

[1] Horvath D.P., Clay S.A., Swanton C.J., Anderson J.V., Chao W.S. (2023). Weed-Induced Crop Yield Loss: A New Paradigm and New Challenges. Trends Plant Sci..

[2] Colbach N., Adeux G., Cordeau S., Moreau D. (2023). Weed-Induced Yield Loss through Resource Competition Cannot Be Sidelined. Trends Plant Sci..

[3] Gharde Y., Singh P.K., Dubey R.P., Gupta P.K. (2018). Assessment of Yield and Economic Losses in Agriculture Due to Weeds in India. Crop Prot..

[4] Kubiak A., Wolna-Maruwka A., Niewiadomska A., Pilarska A.A. (2022). The Problem of Weed Infestation of Agricultural Plantations vs. the Assumptions of the European Biodiversity Strategy. Agronomy.

[5] Das T.K., Behera B., Nath C.P., Ghosh S., Sen S., Raj R., Ghosh S., Sharma A.R., Yaduraju N.T., Nalia A. (2024). Herbicides Use in Crop Production: An Analysis of Cost-Benefit, Non-Target Toxicities and Environmental Risks. Crop Prot..

[6] Gwatidzo V.O., Chipomho J., Parwada C. (2023). Understanding Mechanisms of Herbicide Selectivity in Agro-Ecosystems: A Review. Adv. Chem. Res..

[7] Peterson M.A., Collavo A., Ovejero R., Shivrain V., Walsh M.J. (2018). The Challenge of Herbicide Resistance around the World: A Current Summary. Pest Manag. Sci..

[8] Lan Y., Sun Y., Liu Z., Wei S., Huang H., Cao Y., Li W., Huang Z. (2022). Mechanism of Resistance to Pyroxsulam in Multiple-Resistant Alopecurus Myosuroides from China. Plants.

[9] Heap I. The International Herbicide-Resistant Weed Database. http://www.weedscience.org/.

[10] Heap I. (2014). Global Perspective of Herbicide-Resistant Weeds. Pest Manag. Sci..

[11] Alvarenga E.S., Macías F.A., Ferreira S.S., Galindo J.C.G., Molinillo J.M.G. (2025). Exploring Sesquiterpene Lactones from *Saussurea lappa*: Isolation, Structural Modifications, and Herbicide Bioassay Evaluation. Plants.

[12] Mantilla Afanador J.G., Araujo S.H.C., Teixeira M.G., Lopes D.T., Cerceau C.I., Andreazza F., Oliveira D.C., Bernardi D., Moura W.S., Aguiar R.W.S. (2023). Novel Lactone-Based Insecticides and *Drosophila suzukii* Management: Synthesis, Potential Action Mechanisms and Selectivity for Non-Target Parasitoids. Insects.

[13] Costa T.S., Araujo A.F., Baia V.C., Demuner A.J., Alvarenga E.S. (2025). Asymmetric Synthesis of a Chiral Cyclic Imide: Structure Elucidation via Spectrometric Methods and DFT Calculations, and Biological Assay. J. Mol. Struct..

[14] Torrent K.B.A., Alvarenga E.S. (2021). Synthesis and Identification of Epoxy Derivatives of 5-Methylhexahydroisoindole-1,3-Dione and Biological Evaluation. Molecules.

[15] Gomes S.F., Alvarenga E.S., Baia V.C., Oliveira D.F. (2024). *N*-Phenylnorbornenesuccinimide Derivatives, Agricultural Defensive, and Enzymatic Target Selection. Pest Manag. Sci..

[16] Ramos D., Alvarenga E., Silva J., Cerceau C., Sartori S., Carneiro V., Oliveira D. (2024). Study of the Interference in Target Seed Germination Caused by Dienamides and Epoxy Derivatives, in the Search for New Herbicides. J. Braz. Chem. Soc..

[17] Alvarenga E.S., Carneiro V.M.T., Silva S.A., Siqueira R.P., Bressan G.C. (2019). Synthesis of Novel Amides, Characterization by Spectrometric Methods, Cytotoxic Activity and Theoretical Calculations. J. Mol. Struct..

[18] De Alvarenga E.S., Carneiro V.M.T., Resende G.C., Picanço M.C., Farias E.D.S., Lopes M.C. (2012). Synthesis and Insecticidal Activity of an Oxabicyclolactone and Novel Pyrethroids. Molecules.

[19] Kouame K.B.-J., Savin M.C., Willett C.D., Bertucci M.B., Butts T.R., Grantz E., Roma-Burgos N. (2022). S-Metolachlor Persistence in Soil as Influenced by within-Season and Inter-Annual Herbicide Use. Environ. Adv..

[20] Carvalho-Moore P., Norsworthy J.K., González-Torralva F., Hwang J.-I., Patel J.D., Barber L.T., Butts T.R., McElroy J.S. (2022). Unraveling the Mechanism of Resistance in a Glufosinate-Resistant Palmer Amaranth (*Amaranthus palmeri*) Accession. Weed Sci..

[21] Walsh K.D., Soltani N., Shropshire C., Sikkema P.H. (2015). Weed Control in Soybean with Imazethapyr Applied Alone or in Tank Mix with Saflufenacil/Dimethenamid-P. Weed Sci..

[22] Lewis K.A., Tzilivakis J., Warner D.J., Green A. (2016). An International Database for Pesticide Risk Assessments and Management. Hum. Ecol. Risk Assess. Int. J..

[23] Houk K.N., Liu F., Yang Z., Seeman J.I. (2021). Evolution of the Diels–Alder Reaction Mechanism since the 1930s: Woodward, Houk with Woodward, and the Influence of Computational Chemistry on Understanding Cycloadditions. Angew. Chem. Int. Ed..

[24] Gregoritza M., Brandl F.P. (2015). The Diels–Alder Reaction: A Powerful Tool for the Design of Drug Delivery Systems and Biomaterials. Eur. J. Pharm. Biopharm..

[25] Chen G., Gao J., Huang W., Li Z., Zhao Y. (2022). Synthesis and Bioactivity Evaluation of 5,6-Epoxynorcantharidin Mono-Amide and Imide Derivatives. Monatshefte Chem.-Chem. Mon..

[26] Salvati M.E., Balog A., Shan W., Wei D.D., Pickering D., Attar R.M., Geng J., Rizzo C.A., Gottardis M.M., Weinmann R. (2005). Structure Based Approach to the Design of Bicyclic-1H-Isoindole-1,3(2H)- Dione Based Androgen Receptor Antagonists. Bioorg. Med. Chem. Lett..

[27] Worldwide Protein Data Bank (wwPDB) Full wwPDB X-Ray Structure Validation Report for PDB ID 2DPZ.

[28] Sun C., Robl J.A., Wang T.C., Huang Y., Kuhns J.E., Lupisella J.A., Beehler B.C., Golla R., Sleph P.G., Seethala R. (2006). Discovery of Potent, Orally-Active, and Muscle-Selective Androgen Receptor Modulators Based on an N-Aryl-Hydroxybicyclohydantoin Scaffold. J. Med. Chem..

[29] Ostrowski J., Kuhns J.E., Lupisella J.A., Manfredi M.C., Beehler B.C., Krystek S.R., Bi Y., Sun C., Seethala R., Golla R. (2007). Pharmacological and X-Ray Structural Characterization of a Novel Selective Androgen Receptor Modulator: Potent Hyperanabolic Stimulation of Skeletal Muscle with Hypostimulation of Prostate in Rats. Endocrinology.

[30] Crichlow G.V., Lubetsky J.B., Leng L., Bucala R., Lolis E.J. (2009). Structural and Kinetic Analyses of Macrophage Migration Inhibitory Factor Active Site Interactions. Biochemistry.

[31] DeVore N.M., Smith B.D., Urban M.J., Scott E.E. (2008). Key Residues Controlling Phenacetin Metabolism by Human Cytochrome P450 2A Enzymes. Drug Metab. Dispos..

[32] Chen Y., Shoichet B.K. (2009). Molecular Docking and Ligand Specificity in Fragment-Based Inhibitor Discovery. Nat. Chem. Biol..

[33] Ahmed-Belkacem A., Colliandre L., Ahnou N., Nevers Q., Gelin M., Bessin Y., Brillet R., Cala O., Douguet D., Bourguet W. (2016). Fragment-Based Discovery of a New Family of Non-Peptidic Small-Molecule Cyclophilin Inhibitors with Potent Antiviral Activities. Nat. Commun..

[34] Lee S., Park E.H., Ko H.J., Bang W.G., Kim H.Y., Kim K.H., Choi I.G. (2015). Crystal Structure Analysis of a Bacterial Aryl Acylamidase Belonging to the Amidase Signature Enzyme Family. Biochem. Biophys. Res. Commun..

[35] Unterlass J.E., Baslé A., Blackburn T.J., Tucker J., Cano C., Noble M.E.M., Curtin N.J. (2018). Validating and Enabling Phosphoglycerate Dehydrogenase (PHGDH) as a Target for Fragment-Based Drug Discovery in PHGDH-Amplified Breast Cancer. Oncotarget.

[36] McIntyre P.J., Collins P.M., Vrzal L., Birchall K., Arnold L.H., Mpamhanga C., Coombs P.J., Burgess S.G., Richards M.W., Winter A. (2017). Characterization of Three Druggable Hot-Spots in the Aurora-A/TPX2 Interaction Using Biochemical, Biophysical, and Fragment-Based Approaches. ACS Chem. Biol..

[37] Worldwide Protein Data Bank (wwPDB) Full wwPDB X-Ray Structure Validation Report for PDB ID 5QHU.

[38] Worldwide Protein Data Bank (wwPDB) Full wwPDB X-Ray Structure Validation Report for PDB ID 5QPV.

[39] Nichols C., Ng J., Keshu A., Kelly G., Conte M.R., Marber M.S., Fraternali F., De Nicola G.F. (2020). Mining the PDB for Tractable Cases Where X-Ray Crystallography Combined with Fragment Screens Can Be Used to Systematically Design Protein-Protein Inhibitors: Two Test Cases Illustrated by IL1β-IL1R and P38α-TAB1 Complexes. J. Med. Chem..

[40] Douangamath A., Fearon D., Gehrtz P., Krojer T., Lukacik P., Owen C.D., Resnick E., Strain-Damerell C., Aimon A., Ábrányi-Balogh P. (2020). Crystallographic and Electrophilic Fragment Screening of the SARS-CoV-2 Main Protease. Nat. Commun..

[41] Bradshaw W.J., Kennedy E.C., Moreira T., Smith L.A., Chalk R., Katis V.L., Benesch J.L.P., Brennan P.E., Murphy E.J., Gileadi O. (2024). Regulation of Inositol 5-Phosphatase Activity by the C2 Domain of SHIP1 and SHIP2. Structure.

[42] Worldwide Protein Data Bank (wwPDB) Full wwPDB X-Ray Structure Validation Report for PDB ID 5RYN.

[43] Schuller M., Correy G.J., Gahbauer S., Fearon D., Wu T., Díaz R.E., Young I.D., Carvalho Martins L., Smith D.H., Schulze-Gahmen U. (2021). Fragment Binding to the Nsp3 Macrodomain of SARS-CoV-2 Identified through Crystallographic Screening and Computational Docking. Sci. Adv..

[44] Mühlethaler T., Gioia D., Prota A.E., Sharpe M.E., Cavalli A., Steinmetz M.O. (2021). Comprehensive Analysis of Binding Sites in Tubulin. Angew. Chem.-Int. Ed..

[45] Kany A.M., Sikandar A., Haupenthal J., Yahiaoui S., Maurer C.K., Proschak E., Köhnke J., Hartmann R.W. (2018). Binding Mode Characterization and Early in Vivo Evaluation of Fragment-Like Thiols as Inhibitors of the Virulence Factor LasB from *Pseudomonas aeruginosa*. ACS Infect. Dis..

[46] Simoben C.V., Robaa D., Chakrabarti A., Schmidtkunz K., Marek M., Lancelot J., Kannan S., Melesina J., Shaik T.B., Pierce R.J. (2018). A Novel Class of Schistosoma Mansoni Histone Deacetylase 8 (HDAC8) Inhibitors Identified by Structure-Based Virtual Screening and in Vitro Testing. Molecules.

[47] Carcache D.A., Vulpetti A., Kallen J., Mattes H., Orain D., Stringer R., Vangrevelinghe E., Wolf R.M., Kaupmann K., Ottl J. (2018). Optimizing a Weakly Binding Fragment into a Potent RORÎt Inverse Agonist with Efficacy in an in Vivo Inflammation Model. J. Med. Chem..

[48] Wolter M., Valenti D., Cossar P.J., Levy L.M., Hristeva S., Genski T., Hoffmann T., Brunsveld L., Tzalis D., Ottmann C. (2020). Fragment-Based Stabilizers of Protein–Protein Interactions through Imine-Based Tethering. Angew. Chem.-Int. Ed..

[49] Zhao Y., Mahy W., Willis N.J., Woodward H.L., Steadman D., Bayle E.D., Atkinson B.N., Sipthorp J., Vecchia L., Ruza R.R. (2022). Structural Analysis and Development of Notum Fragment Screening Hits. ACS Chem. Neurosci..

[50] Liu H., Forouhar F., Lin A.J., Wang Q., Polychronidou V., Soni R.K., Xia X., Stockwell B.R. (2022). Small-Molecule Allosteric Inhibitors of GPX4. Cell Chem. Biol..

[51] St. Denis J.D., Chessari G., Cleasby A., Cons B.D., Cowan S., Dalton S.E., East C., Murray C.W., O’Reilly M., Peakman T. (2022). X-Ray Screening of an Electrophilic Fragment Library and Application toward the Development of a Novel ERK 1/2 Covalent Inhibitor. J. Med. Chem..

[52] Atkinson B.N., Willis N.J., Zhao Y., Patel C., Frew S., Costelloe K., Magno L., Svensson F., Jones E.Y., Fish P.V. (2023). Designed Switch from Covalent to Non-Covalent Inhibitors of Carboxylesterase Notum Activity. Eur. J. Med. Chem..

[53] Wang W., Zhang H., Wang X., Patterson J., Winter P., Graham K., Ghosh S., Lee J.C., Katsetos C.D., Mackey J.R. (2017). Novel Mutations Involving ΒI-, ΒIIA-, or ΒIVB-Tubulin Isotypes with Functional Resemblance to ΒIII-Tubulin in Breast Cancer. Protoplasma.

[54] Hammond J.W., Cai D., Verhey K.J. (2008). Tubulin Modifications and Their Cellular Functions. Curr. Opin. Cell Biol..

[55] Wasteneys G.O. (2002). Microtubule Organization in the Green Kingdom: Chaos or Self-Order?. J. Cell Sci..

[56] Morejohn L.C., Bureau T.E., Molè-Bajer J., Bajer A.S., Fosket D.E. (1987). Oryzalin, a Dinitroaniline Herbicide, Binds to Plant Tubulin and Inhibits Microtubule Polymerization in Vitro. Planta.

[57] Hugdahl J.D., Morejohn L.C. (1993). Rapid and Reversible High-Affinity Binding of the Dinitroaniline Herbicide Oryzalin to Tubulin from *Zea mays* L.. Plant Physiol..

[58] Giles N.L., Armson A., Reid S.A. (2009). Characterization of Trifluralin Binding with Recombinant Tubulin from *Trypanosoma brucei*. Parasitol. Res..

[59] Duke S.O., Powles S.B. (2008). Glyphosate: A Once-in-a-Century Herbicide. Pest Manag. Sci..

[60] Tresch S., Plath P., Grossmann K. (2005). Herbicidal Cyanoacrylates with Antimicrotubule Mechanism of Action. Pest Manag. Sci..

[61] Nyporko A.Y., Blume Y.B. (2014). Structural Mechanisms of Interaction of Cyanolcrylates with Plant Tubulin. Cytol. Genet..

[62] Prota A.E., Danel F., Bachmann F., Bargsten K., Buey R.M., Pohlmann J., Reinelt S., Lane H., Steinmetz M.O. (2014). The Novel Microtubule-Destabilizing Drug BAL27862 Binds to the Colchicine Site of Tubulin with Distinct Effects on Microtubule Organization. J. Mol. Biol..

[63] Ravelli R.B.G., Gigant B., Curmi P.A., Jourdain I., Lachkar S., Sobel A., Knossow M. (2004). Insight into Tubulin Regulation from a Complex with Colchicine and a Stathmin-like Domain. Nature.

[64] Ranaivoson F.M., Gigant B., Berritt S., Joullié M., Knossow M. (2012). Structural Plasticity of Tubulin Assembly Probed by Vinca-Domain Ligands. Acta Crystallogr. D Biol. Crystallogr..

[65] Wang Y., Zhang H., Gigant B., Yu Y., Wu Y., Chen X., Lai Q., Yang Z., Chen Q., Yang J. (2016). Structures of a Diverse Set of Colchicine Binding Site Inhibitors in Complex with Tubulin Provide a Rationale for Drug Discovery. FEBS J..

[66] Canela M.D., Noppen S., Bueno O., Prota A.E., Bargsten K., Sáez-Calvo G., Jimeno M.L., Benkheil M., Ribatti D., Velázquez S. (2017). Antivascular and Antitumor Properties of the Tubulin-Binding Chalcone TUB091. Oncotarget.

[67] Marangon J., Christodoulou M.S., Casagrande F.V.M., Tiana G., Dalla Via L., Aliverti A., Passarella D., Cappelletti G., Ricagno S. (2016). Tools for the Rational Design of Bivalent Microtubule-Targeting Drugs. Biochem. Biophys. Res. Commun..

[68] Gaspari R., Prota A.E., Bargsten K., Cavalli A., Steinmetz M.O. (2017). Structural Basis of Cis- and Trans-Combretastatin Binding to Tubulin. Chem.

[69] Field J.J., Pera B., Gallego J.E., Calvo E., Rodríguez-Salarichs J., Sáez-Calvo G., Zuwerra D., Jordi M., Andreu J.M., Prota A.E. (2018). Zampanolide Binding to Tubulin Indicates Cross-Talk of Taxane Site with Colchicine and Nucleotide Sites. J. Nat. Prod..

[70] Dohle W., Jourdan F.L., Menchon G., Prota A.E., Foster P.A., Mannion P., Hamel E., Thomas M.P., Kasprzyk P.G., Ferrandis E. (2018). Quinazolinone-Based Anticancer Agents: Synthesis, Antiproliferative SAR, Antitubulin Activity, and Tubulin Co-Crystal Structure. J. Med. Chem..

[71] Muzaffar A., Brossi A., Lin C.M., Hamel E. (1990). Antitubulin Effects of Derivatives of 3-Demethylthiocolchicine, Methylthio Ethers of Natural Colchicinoids, and Thioketones Derived from Thiocolchicine. Comparison with Colchicinoids. J. Med. Chem..

[72] Cheng J., Wu Y., Wang Y., Wang C., Wang Y., Wu C., Zeng S., Yu Y., Chen Q. (2018). Structure of a Benzylidene Derivative of 9(10H)-Anthracenone in Complex with Tubulin Provides a Rationale for Drug Design. Biochem. Biophys. Res. Commun..

[73] Li Z., Ma L., Wu C., Meng T., Ma L., Zheng W., Yu Y., Chen Q., Yang J., Shen J. (2018). The Structure of MT189-Tubulin Complex Provides Insights into Drug Design. Lett. Drug Des. Discov..

[74] Brindisi M., Ulivieri C., Alfano G., Gemma S., de Asís Balaguer F., Khan T., Grillo A., Chemi G., Menchon G., Prota A.E. (2019). Structure-Activity Relationships, Biological Evaluation and Structural Studies of Novel Pyrrolonaphthoxazepines as Antitumor Agents. Eur. J. Med. Chem..

[75] Li Y., Yang J., Niu L., Hu D., Li H., Chen L., Yu Y., Chen Q. (2020). Structural Insights into the Design of Indole Derivatives as Tubulin Polymerization Inhibitors. FEBS Lett..

[76] Niu L., Yang J., Yan W., Yu Y., Zheng Y., Ye H., Chen Q., Chen L. (2019). Reversible Binding of the Anticancer Drug KXO1 (Tirbanibulin) to the Colchicine-Binding Site of β-Tubulin Explains KXO1’s Low Clinical Toxicity. J. Biol. Chem..

[77] Sáez-Calvo G., Sharma A., Balaguer F.d.A., Barasoain I., Rodríguez-Salarichs J., Olieric N., Muñoz-Hernández H., Berbís M.Á., Wendeborn S., Peñalva M.A. (2017). Triazolopyrimidines Are Microtubule-Stabilizing Agents That Bind the Vinca Inhibitor Site of Tubulin. Cell Chem. Biol..

[78] Doodhi H., Prota A.E., Rodríguez-García R., Xiao H., Custar D.W., Bargsten K., Katrukha E.A., Hilbert M., Hua S., Jiang K. (2016). Termination of Protofilament Elongation by Eribulin Induces Lattice Defects That Promote Microtubule Catastrophes. Curr. Biol..

[79] Alhossary A., Handoko S.D., Mu Y., Kwoh C.K. (2015). Fast, Accurate, and Reliable Molecular Docking with QuickVina 2. Bioinformatics.

[80] O’Boyle N.M., Banck M., James C.A., Morley C., Vandermeersch T., Hutchison G.R. (2011). Open Babel. J. Cheminform..

[81] Tosco P., Balle T., Shiri F. (2011). Open3DALIGN: An Open-Source Software Aimed at Unsupervised Ligand Alignment. J. Comput. Aided Mol. Des..

[82] Stewart J.J.P., Stewart A.C. (2023). A Semiempirical Method Optimized for Modeling Proteins. J. Mol. Model..

[83] Fráguas R.M., Costa V.A., Terra W.C., Aguiar A.P., Martins S.J., Campos V.P., Oliveira D.F. (2020). Toxicities of 4,5-Dihydroisoxazoles against Root-Knot Nematodes and in Silico Studies of Their Modes of Action. J. Agric. Food Chem..

[84] Lešnik S., Štular T., Brus B., Knez D., Gobec S., Janežič D., Konc J. (2015). LiSiCA: A Software for Ligand-Based Virtual Screening and Its Application for the Discovery of Butyrylcholinesterase Inhibitors. J. Chem. Inf. Model..

[85] Feng Z., Chen L., Maddula H., Akcan O., Oughtred R., Berman H.M., Westbrook J. (2004). Ligand Depot: A Data Warehouse for Ligands Bound to Macromolecules. Bioinformatics.

[86] Saur I.M.L., Panstruga R., Schulze-Lefert P. (2021). NOD-like Receptor-Mediated Plant Immunity: From Structure to Cell Death. Nat. Rev. Immunol..

[87] Yamada Y., Gohda S., Abe K., Togo T., Shimano N., Sasaki T., Tanaka H., Ono H., Ohba T., Kubo S. (2017). Carbon Materials with Controlled Edge Structures. Carbon.

[88] Martínez L., Andreani R., Martínez J.M. (2007). Convergent Algorithms for Protein Structural Alignment. BMC Bioinform..

[89] Pettersen E.F., Goddard T.D., Huang C.C., Couch G.S., Greenblatt D.M., Meng E.C., Ferrin T.E. (2004). UCSF Chimera—A Visualization System for Exploratory Research and Analysis. J. Comput. Chem..

[90] Allouche A. (2012). Software News and Updates Gabedit—A Graphical User Interface for Computational Chemistry Softwares. J. Comput. Chem..

[91] Maderna A., Doroski M., Subramanyam C., Porte A., Leverett C.A., Vetelino B.C., Chen Z., Risley H., Parris K., Pandit J. (2014). Discovery of Cytotoxic Dolastatin 10 Analogues with N-Terminal Modifications. J. Med. Chem..

[92] Waight A.B., Bargsten K., Doronina S., Steinmetz M.O., Sussman D., Prota A.E. (2016). Structural Basis of Microtubule Destabilization by Potent Auristatin Anti-Mitotics. PLoS ONE.

[93] Prota A.E., Bargsten K., Redondo-Horcajo M., Smith A.B., Yang C.P.H., McDaid H.M., Paterson I., Horwitz S.B., Fernando Díaz J., Steinmetz M.O. (2017). Structural Basis of Microtubule Stabilization by Discodermolide. ChemBioChem.

[94] Berman H., Henrick K., Nakamura H. (2003). Announcing the Worldwide Protein Data Bank. Nat. Struct. Biol..

[95] Yang J., Yan W., Yu Y., Wang Y., Yang T., Xue L., Yuan X., Long C., Liu Z., Chen X. (2018). The Compound Millepachine and Its Derivatives Inhibit Tubulin Polymerization by Irreversibly Binding to the Colchicine-Binding Site in β-Tubulin. J. Biol. Chem..

[96] Shapiro S.S., Wilk M.B. (1965). An Analysis of Variance Test for Normality (Complete Samples). Biometrika.

[97] Bartlett M.S. (1937). Properties of Sufficiency and Statistical Tests. Proc. R. Soc. Lond. A Math. Phys. Sci..

[98] Kruskal W.H., Wallis W.A. (1952). Use of Ranks in One-Criterion Variance Analysis. J. Am. Stat. Assoc..

[99] (1915). University of California. J. Educ..

[100] Thorsten P. (2023). R Package “PMCMRplus”. Calculate Pairwise Multiple Comparisons of Mean Rank Sums Extended, version 1.9.10. https://cran.r-project.org/.

