# Supplementary material for: Synthesis of Epoxyoxirenes: Phytotoxic Activity and Enzymatic Target Identification
Source: Plants (Basel). 2025 Jun 24;14(13):1933. doi: 10.3390/plants14131933 (PMC12251973; doi:10.3390/plants14131933)
Supplement: Supplementary file 1 [file plants-14-01933-s001.zip › plants-3684951-supplementary.pdf]

Supplementary Material

Synthesis of Epoxyoxirenes: Phytotoxic Activity and Enzymatic Target Identification

Kamylla C. F. de Faria, Elson S. Alvarenga, Denilson F. Oliveira, Vitor C. Baia, and Armin F. Isenmann

[1]Department of Chemistry, Universidade Federal de Viçosa, Viçosa 36570-900, MG, Brazil; kamyllacalzolariferreira@gmail.com (K.C.F.F.); elson@ufv.br (ESA); vitor@ufv.br (V.C.B.)
[2]Department of Chemistry, Universidade Federal de Lavras, Lavras 37203-202, MG, Brazil; denilson@ufla.br
[3]Department of Metallurgy and Chemistry, Centro Federal de Educação Tecnológica de Minas Gerais, Timoteo 35180-008, MG, Brazil; armincefetmg@gmail.com
*Correspondence: elson@ufv.br

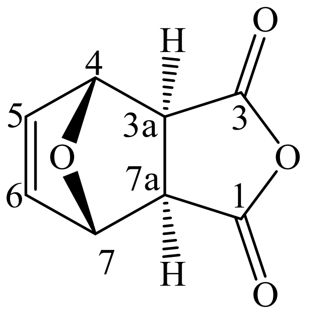

Figure S1. (3a*R*,4*S*,7*R*,7a*S*)-3a,4,7,7a-tetrahydro-4,7-epoxyisobenzofuran-1,3-dione (**1**).

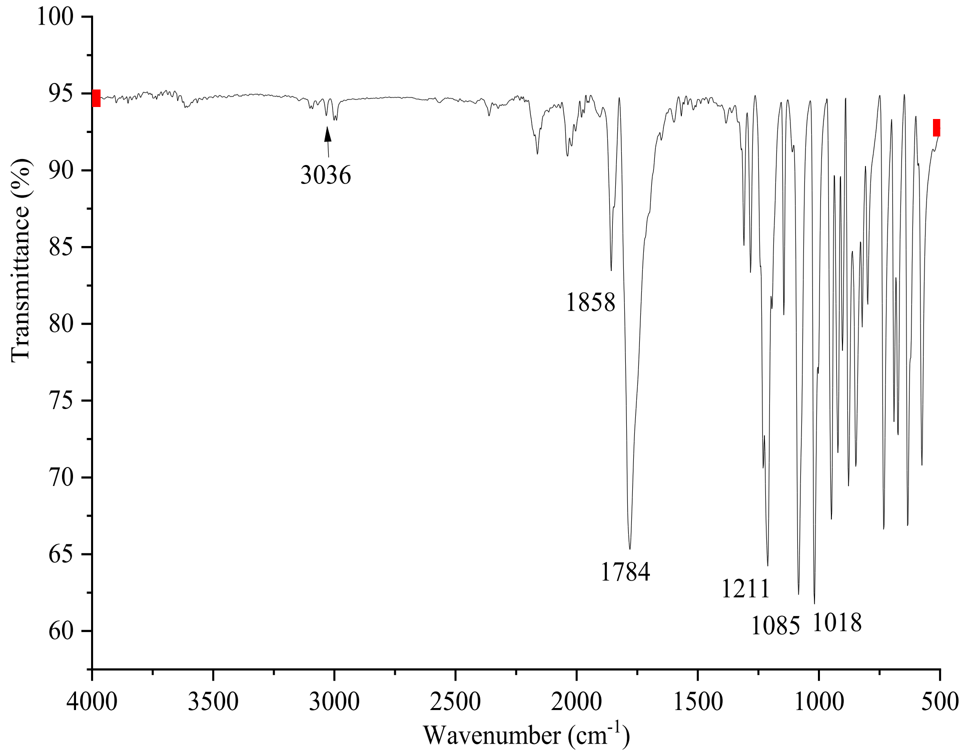

Figure S2. Infrared of precursor **1** by spectrophotometer *FT-IR VARIAN 660-ATR*.

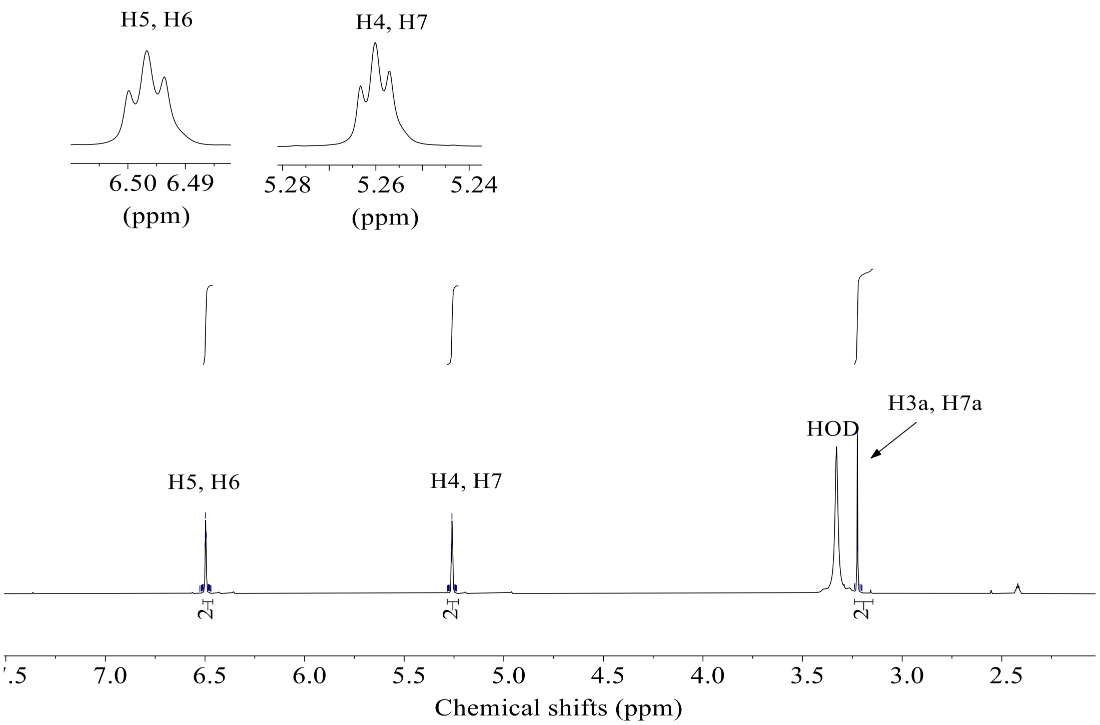

Figure S3. ^{1}H NMR (300 MHz; DMSO-d_6; δ= 3.3 ppm) of precursor **1**.

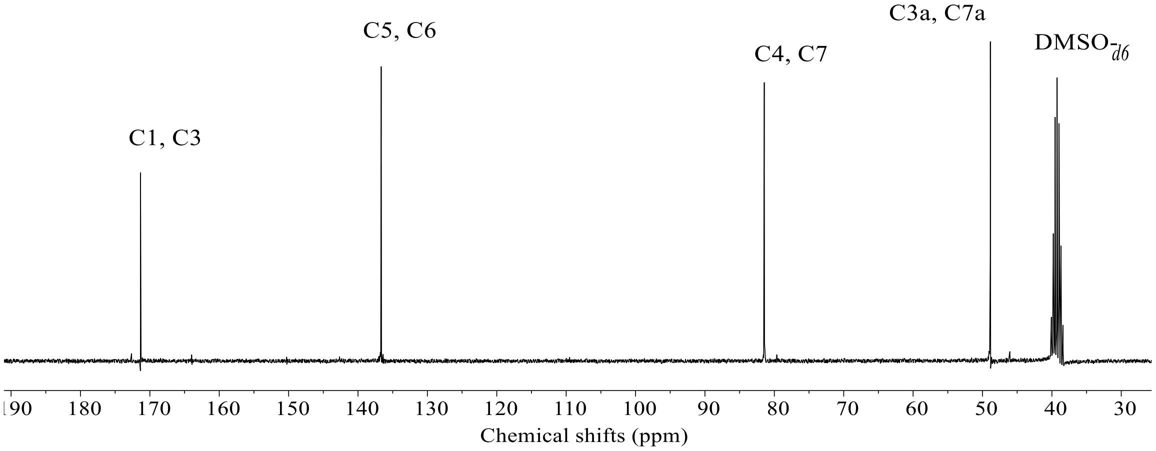

Figure S4. ^{13}C NMR (75 MHz; DMSO-d_6; δ= 40.0 ppm) of precursor **1**.

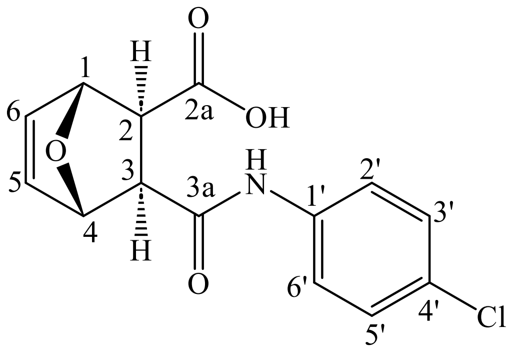

Figure S5. (1*R*,2*S*,3*R*,4*S*)-3-((4-chlorophenyl)carbamoyl)-7-oxabicyclo[2.2.1]hept-5-ene-2-carboxylic acid (**2**).

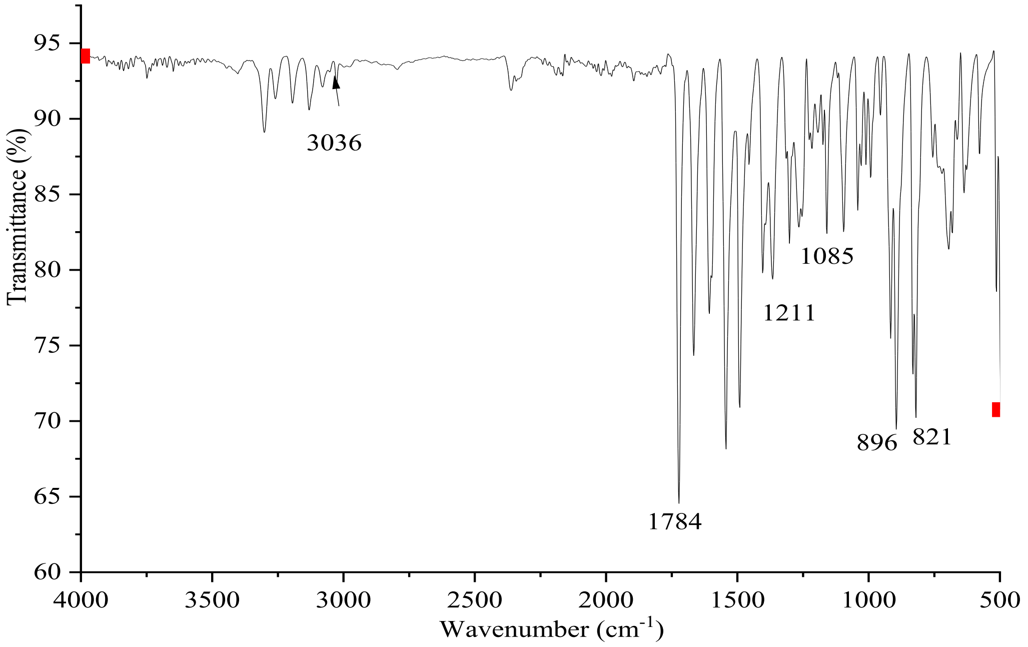

Figure S6. Infrared of compound **2** by spectrophotometer *FT-IR VARIAN 660-ATR.*

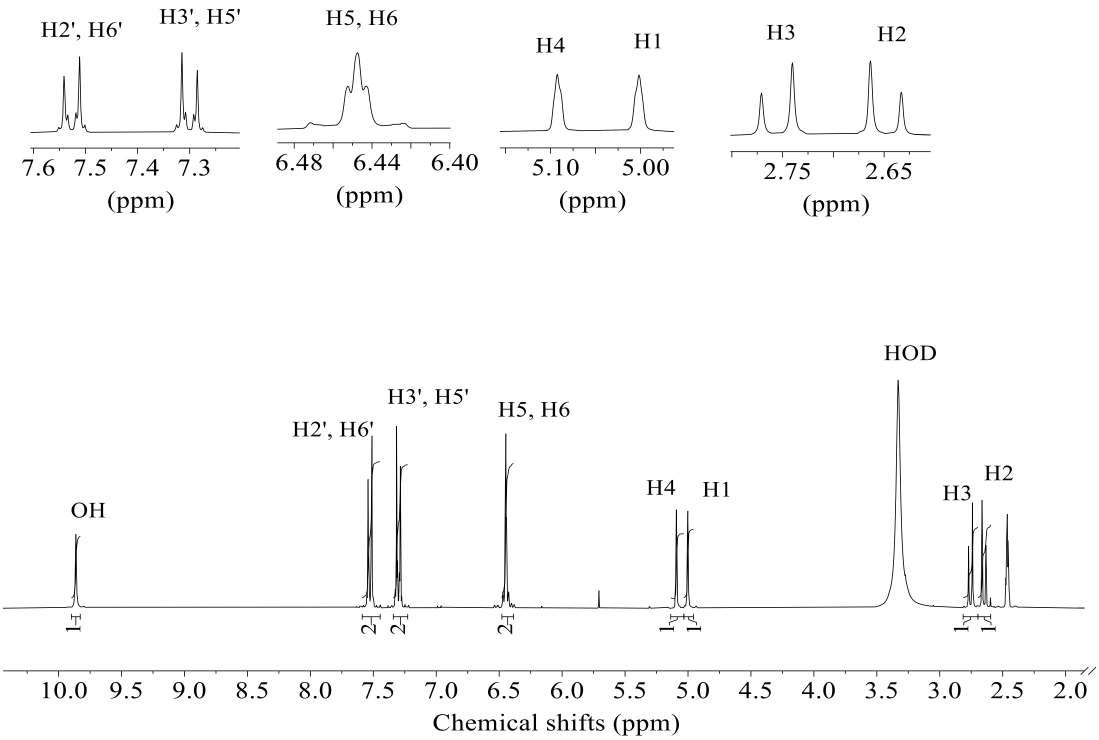

Figure S7. ^{1}H NMR (300 MHz; DMSO-d_6; δ= 3,3 ppm) of compound **2**.

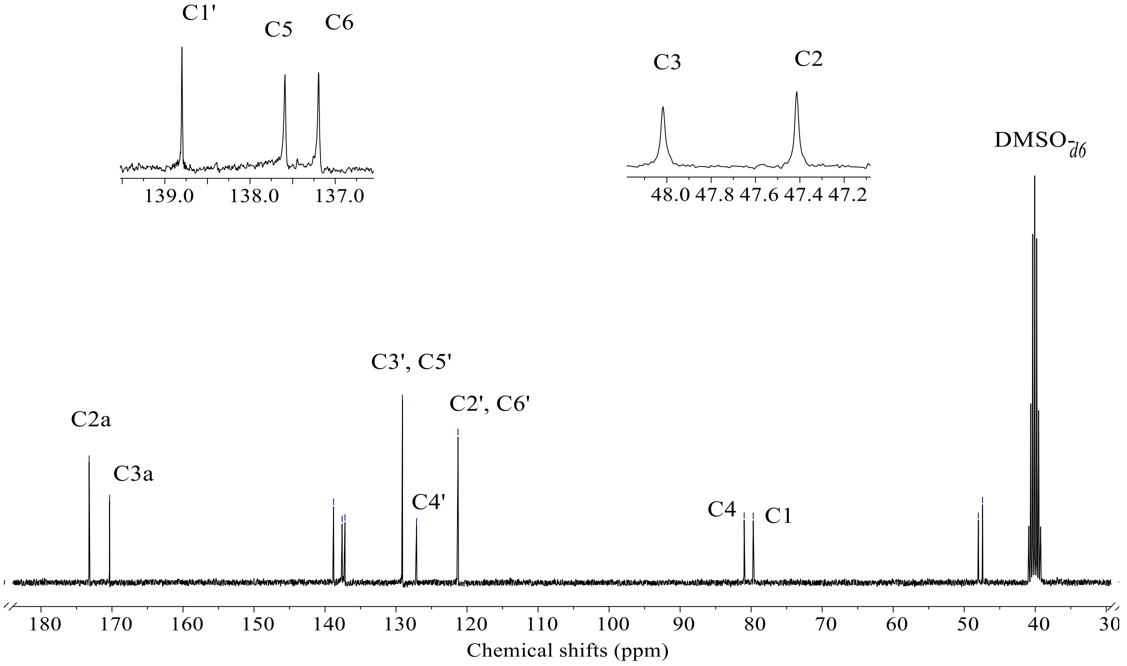

Figure S8. ^{13}C NMR (75 MHz; DMSO-d_6; δ= 40,0 ppm) of compound **2**.

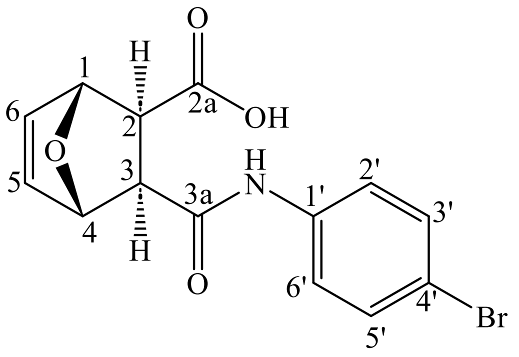

Figure S9. (1*R*,2*S*,3*R*,4*S*)-3-((4-bromophenyl)carbamoyl)-7-oxabicyclo[2.2.1]hept-5-ene-2-carboxylic acid (**3**).

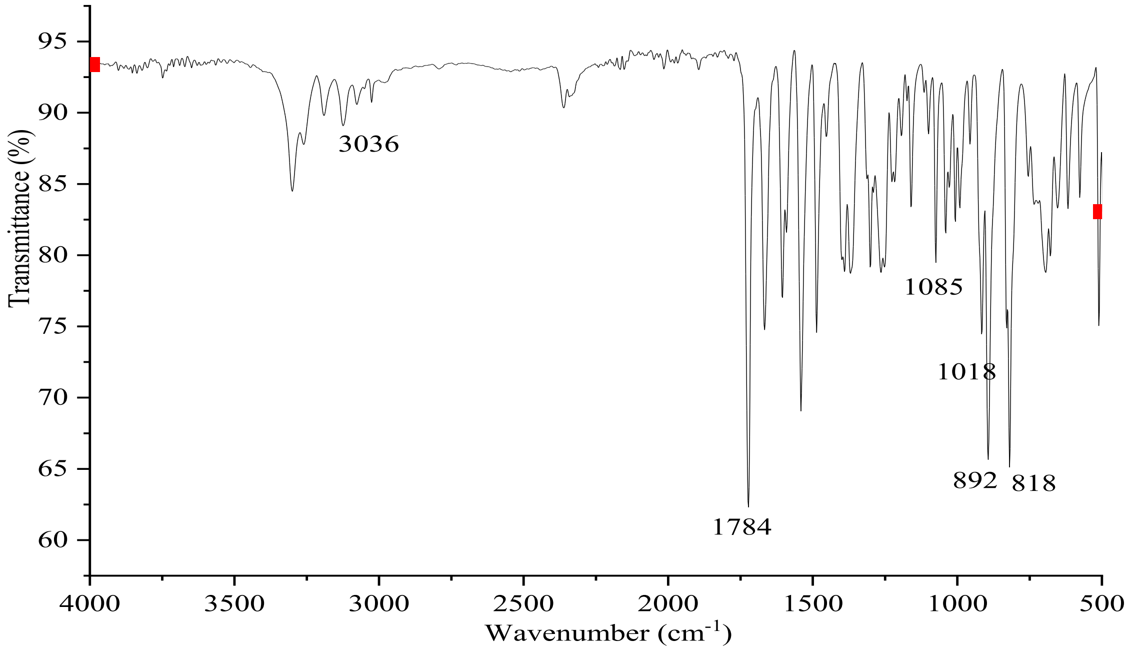

Figure S10. Infrared of compound **3** by spectrophotometer *FT-IR VARIAN 660-ATR*.

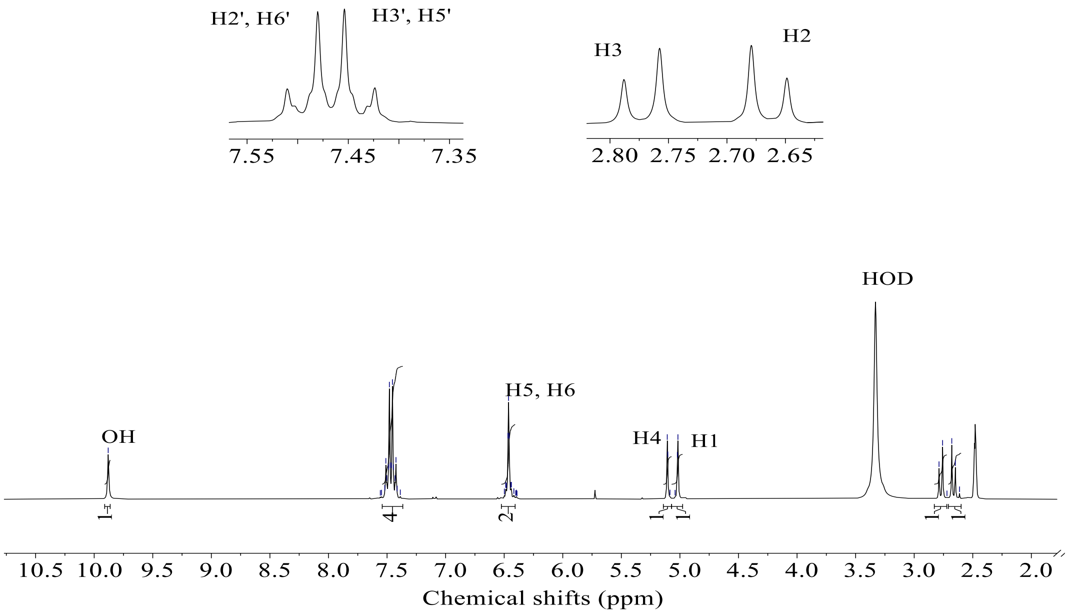

Figure S11. ^{1}H NMR (300 MHz; DMSO-d_6; δ= 3,3 ppm) of compound **3**.

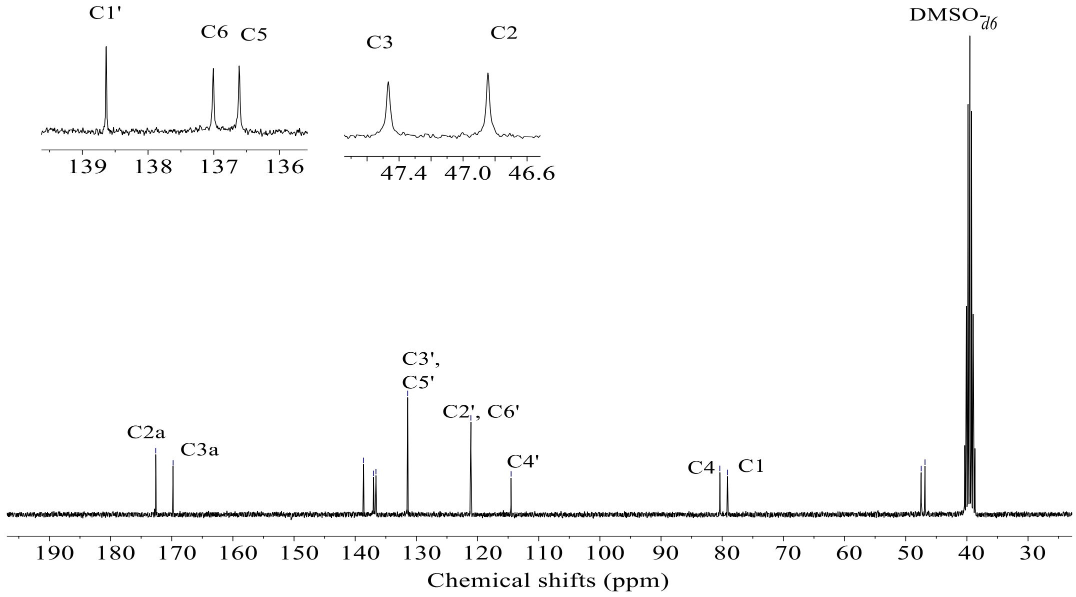

Figure S12. ^{13}C NMR (75 MHz; DMSO-d_6; δ= 40,0 ppm) of compound **3**.

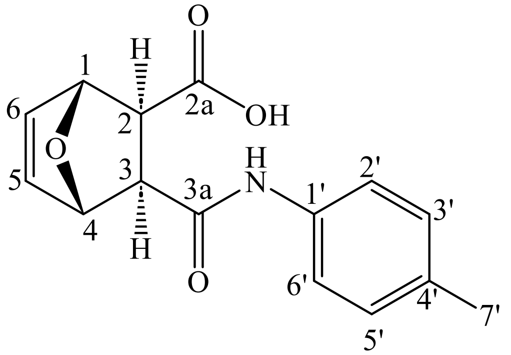

Figure S13. (1*R*,2*S*,3*R*,4*S*)-4-(p-tolylcarbamoyl)-7-oxabyciclo[2.2.1]hept-1-ene-5-caboxilic acid (**4**).

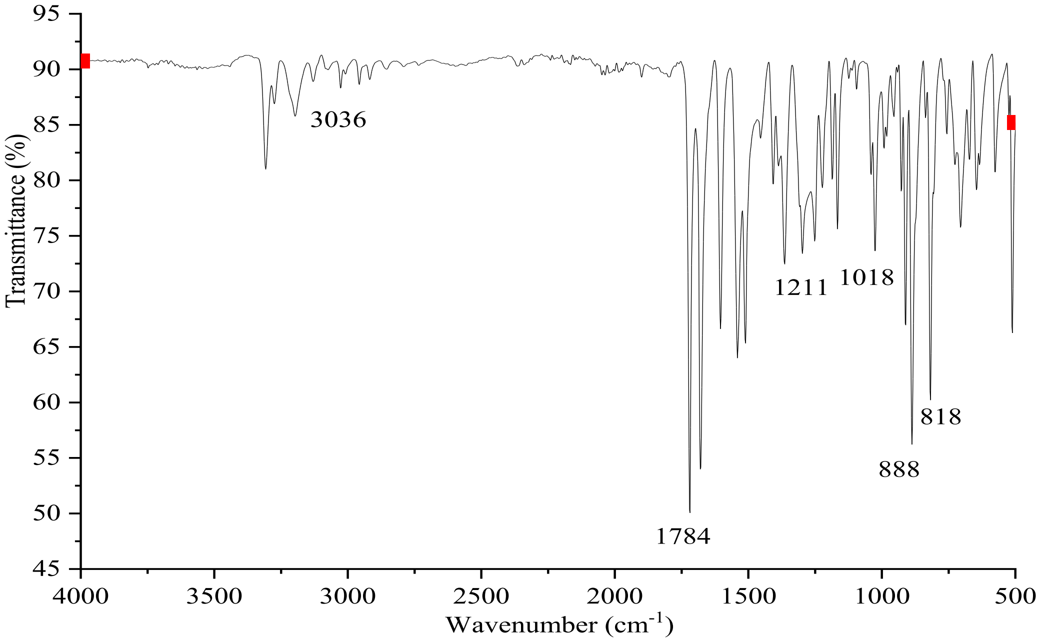

Figure S14. Infrared of compound **4** by spectrophotometer *FT-IR VARIAN 660-ATR*.

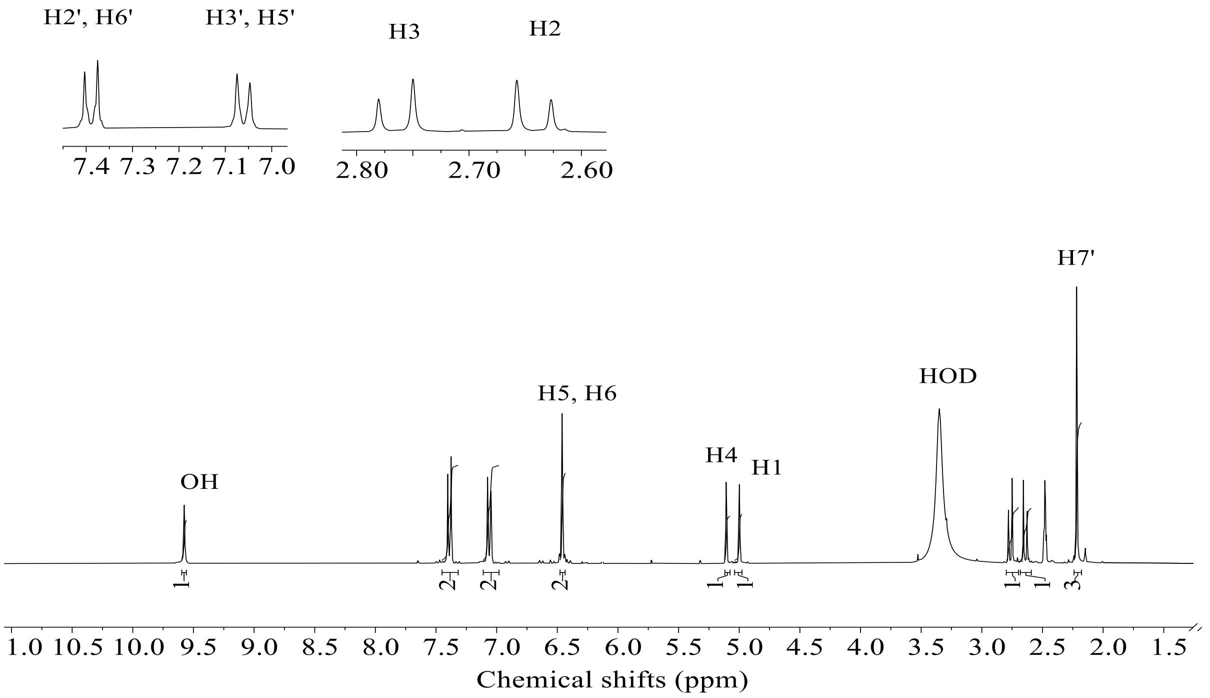

Figure S15. ^{1}H NMR (300 MHz; DMSO-d_6; δ= 3,3 ppm) of compound **4**.

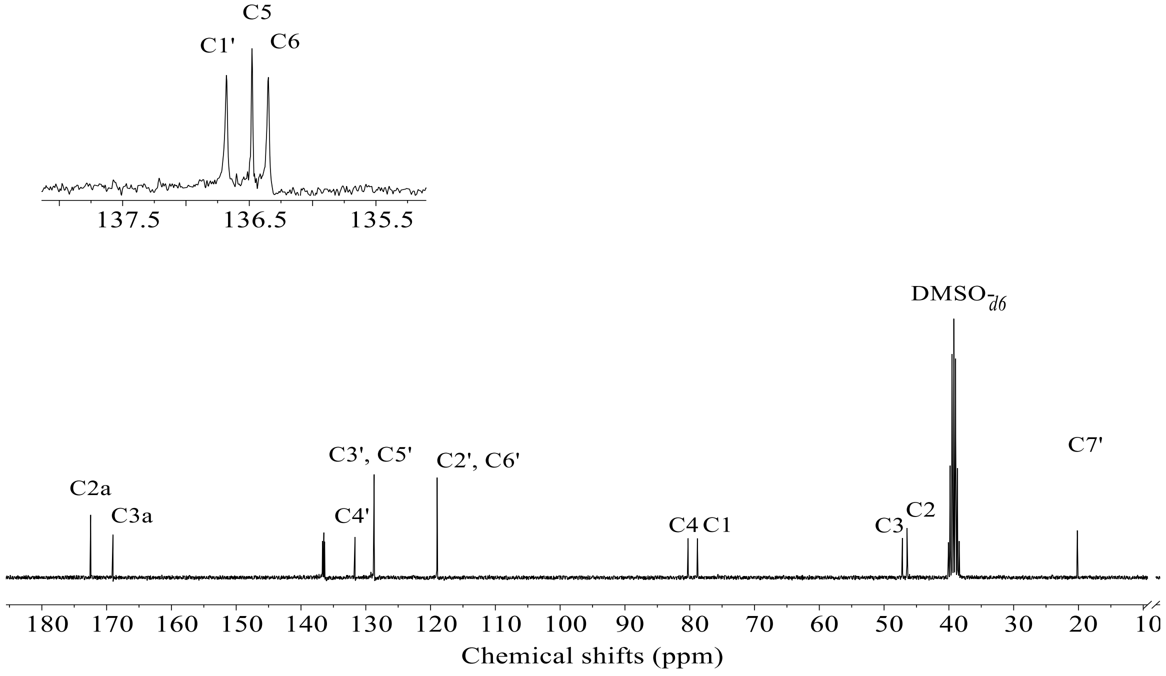

Figure S16. ^{13}C NMR (75 MHz; DMSO-d_6; δ= 40,0 ppm) of compound **4**.

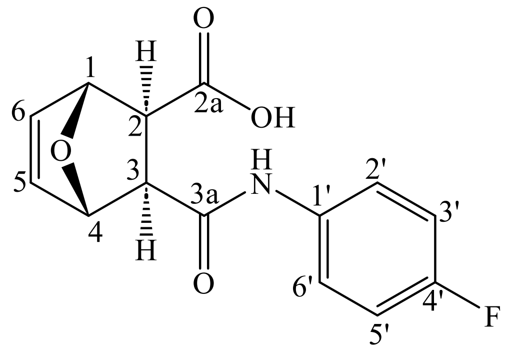

Figure S17. (1*R*,2*S*,3*R*,4*S*)-3-((4-fluorophenyl)carbamoyl)-7-oxabyciclo[2.2.1]hept-5-ene-2-caboxilic acid (**5**).

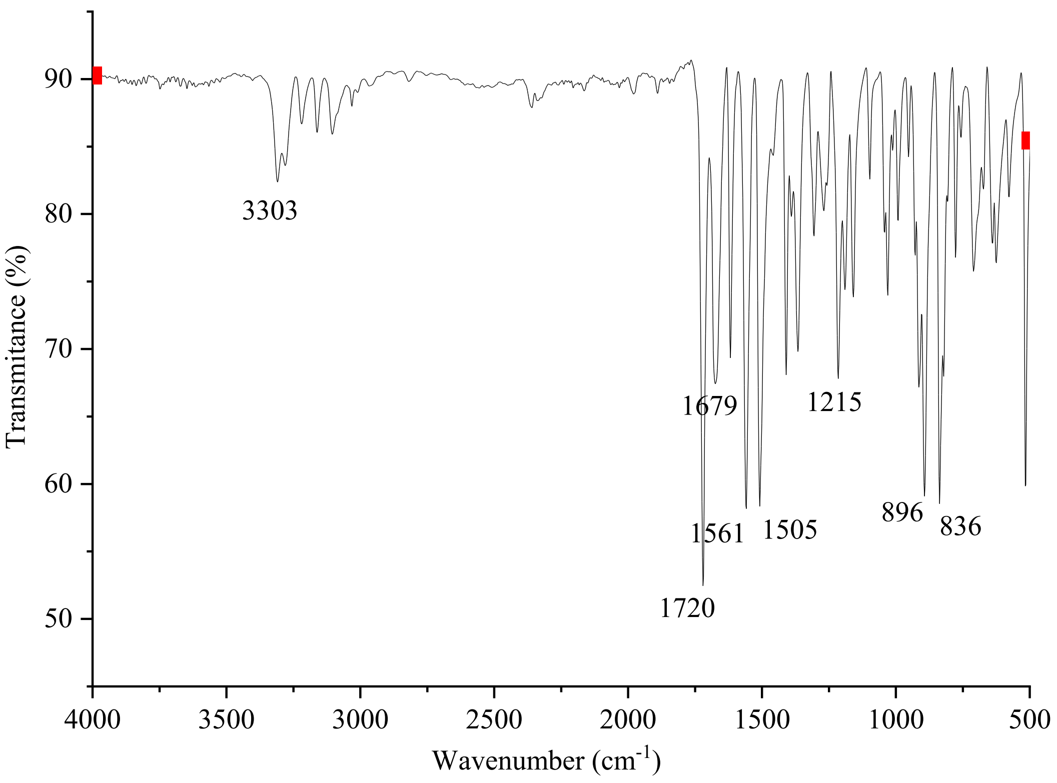

Figure S18. Infrared of compound **5** by spectrophotometer *FT-IR VARIAN 660-ATR*.

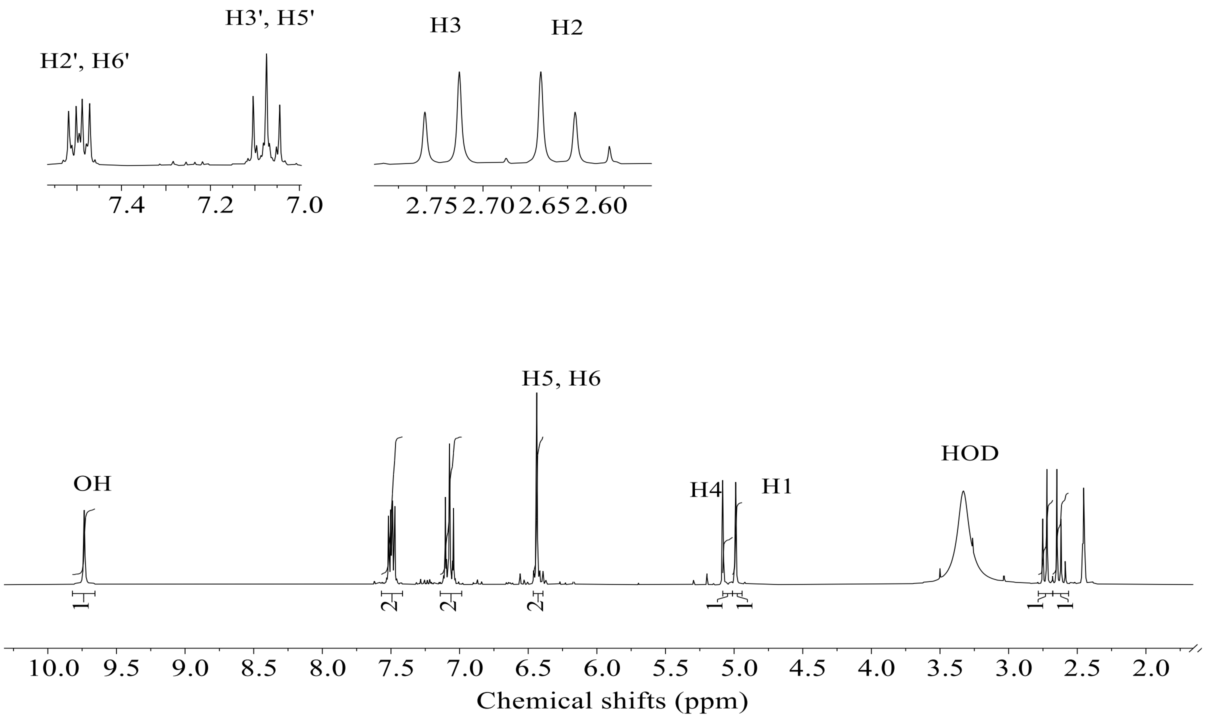

Figure S19. ^{1}H NMR (300 MHz; DMSO-d_6; δ= 3,3 ppm) of compound **5**.

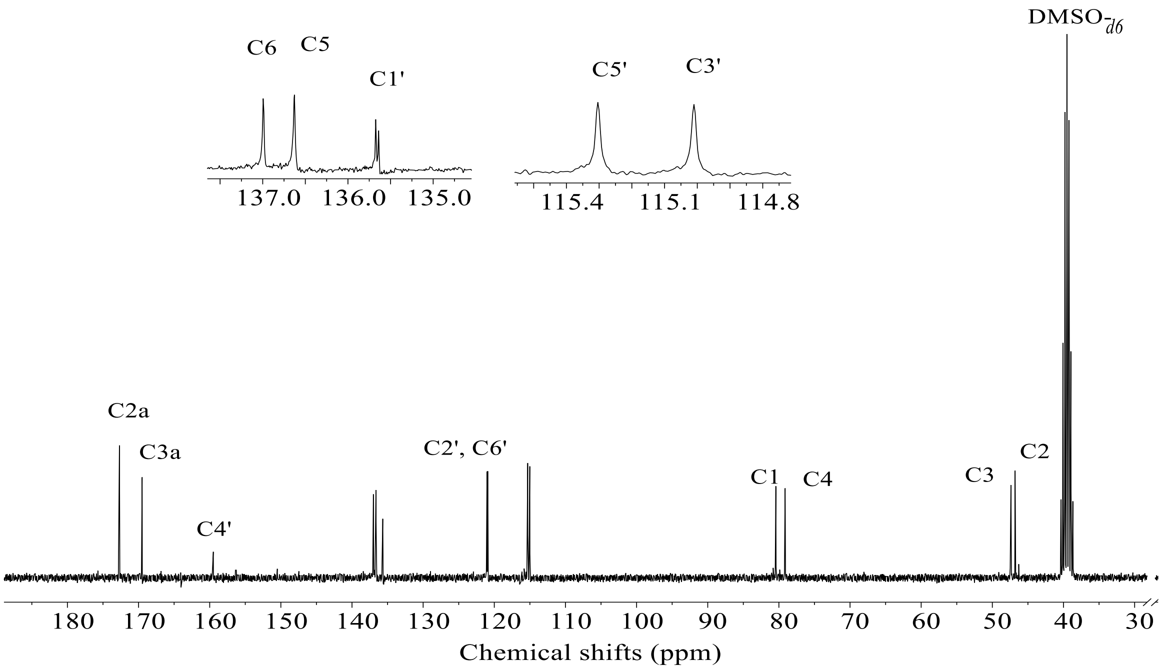

Figure S20. ^{13}C NMR (75 MHz; DMSO-d_6; δ= 40,0 ppm) of compound **5**.

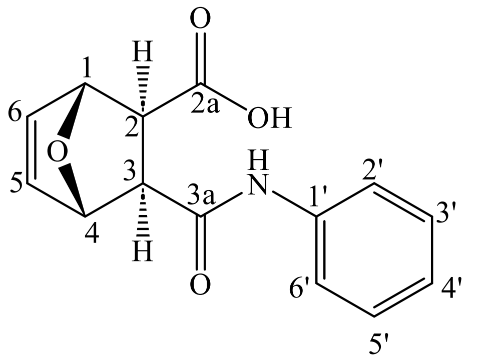

Figure S21. (1*R*,2*S*,3*R*,4*S*)-3-(phenylcarbamoyl)-7-oxabyciclo[2.2.1]hept-5-ene-2-caboxilic acid (**6**).

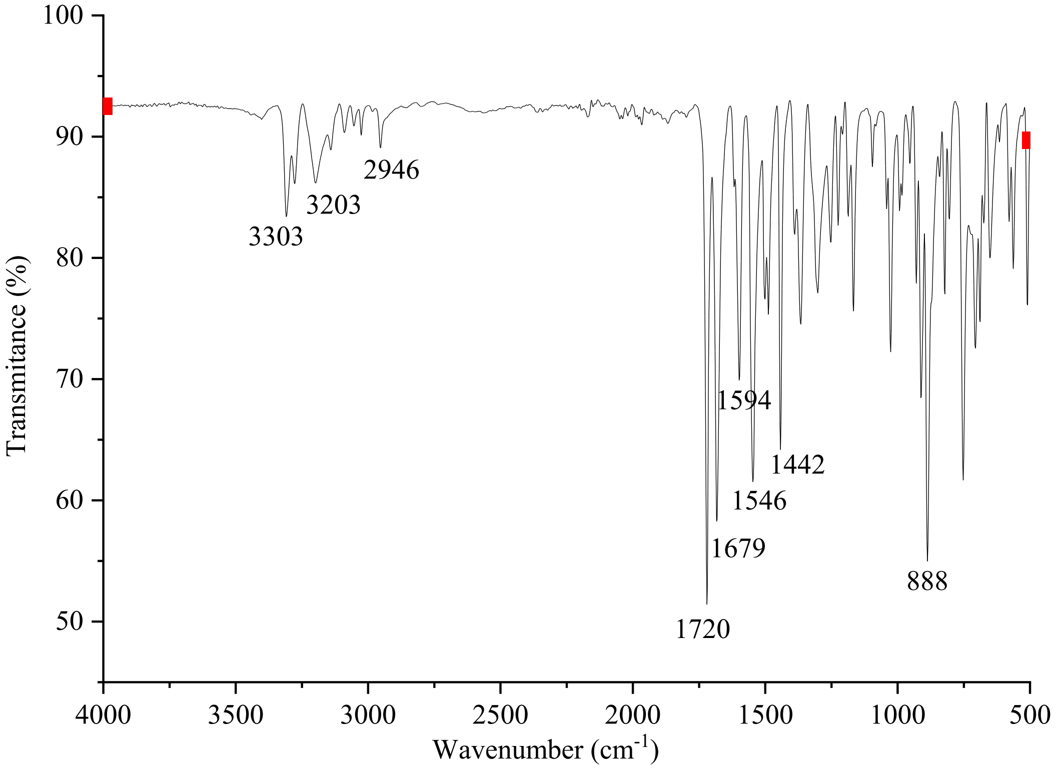

Figure S22. Infrared of compound **6** by spectrophotometer *FT-IR VARIAN 660-ATR.*

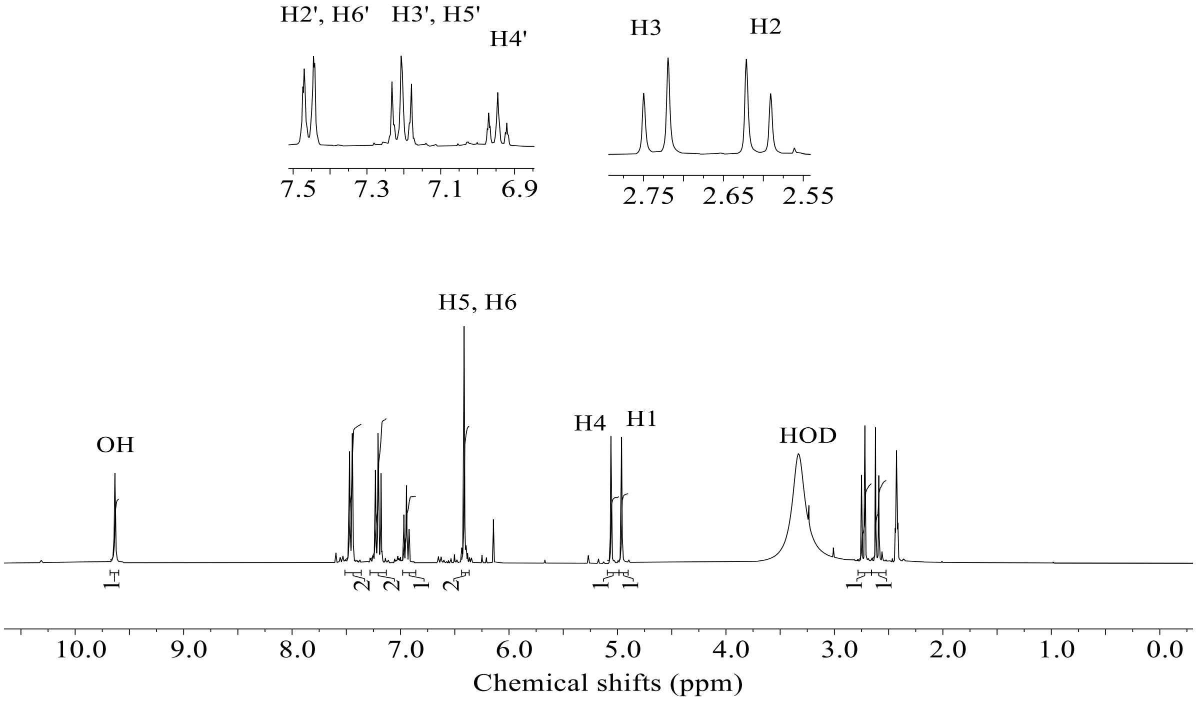

Figure S23. ^{1}H NMR (300 MHz; DMSO-d_6; δ= 3,3 ppm) of compound **6**.

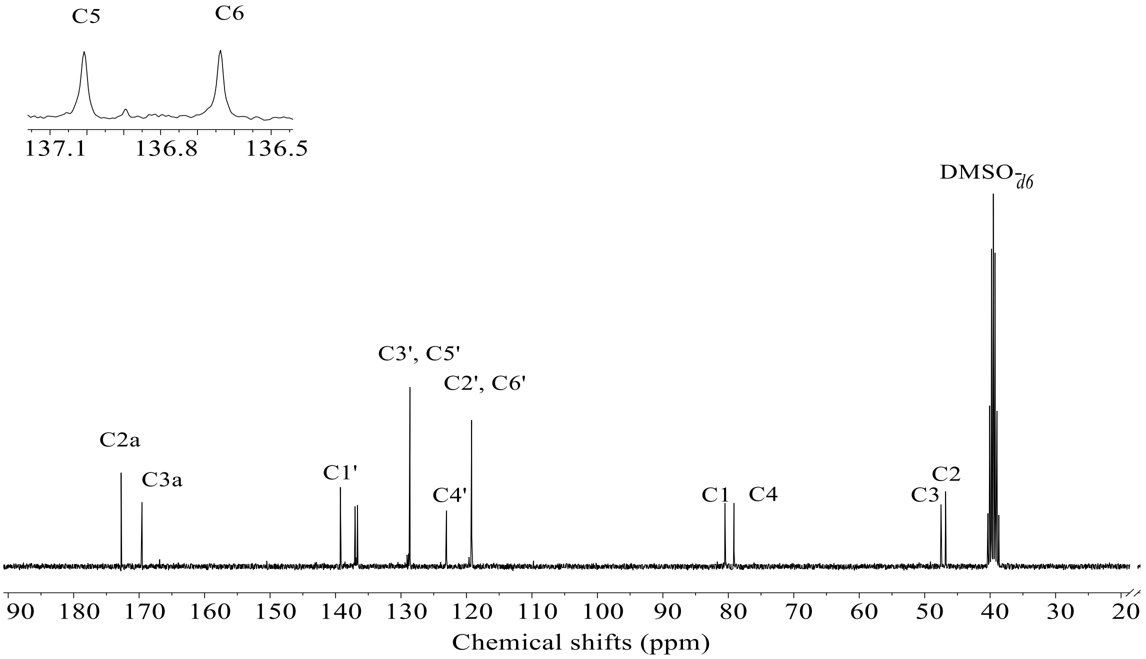

Figure S24. ^{13}C NMR (75 MHz; DMSO-d_6; δ= 40,0 ppm) of compound **6**.

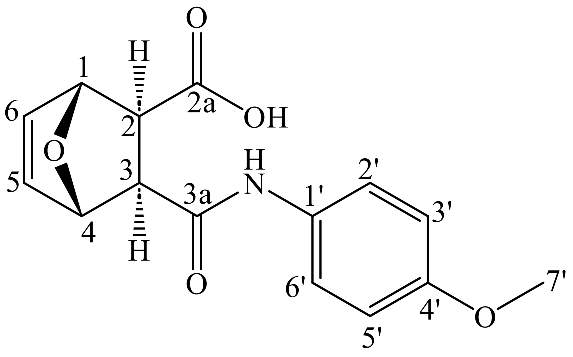

Figure S25. (1*R*,2*S*,3*R*,4*S*)-3-((4-methoxyphenyl)carbamoyl)-7-oxabyciclo[2.2.1]hept-5-ene-2-caboxilic acid (**7**).

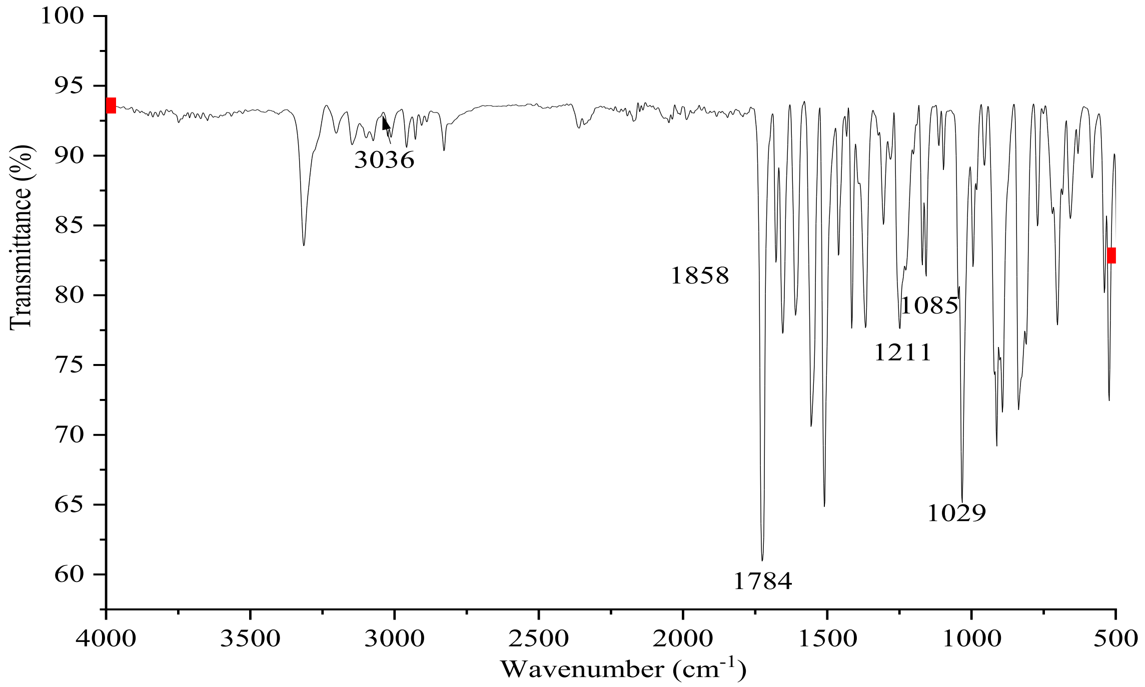

Figure S26. Infrared of compound **7** by spectrophotometer *FT-IR VARIAN 660-ATR*.

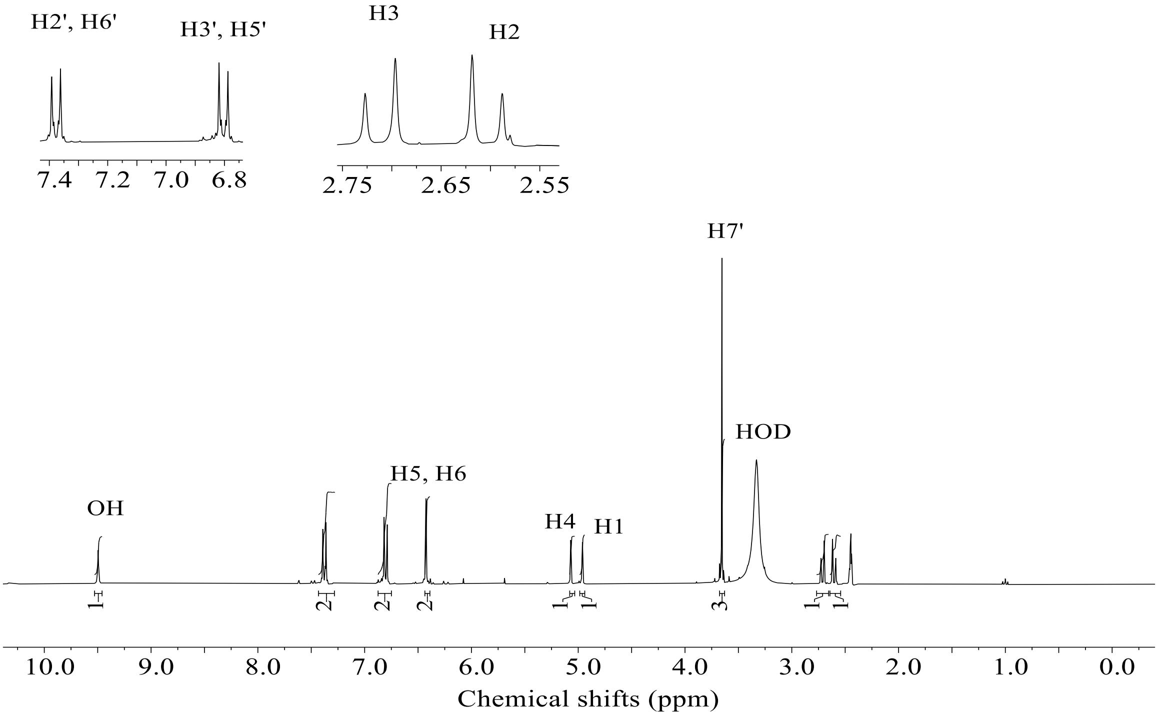

Figure S27. ^{1}H NMR (300 MHz; DMSO-d_6; δ= 3,3 ppm) of compound **7**.

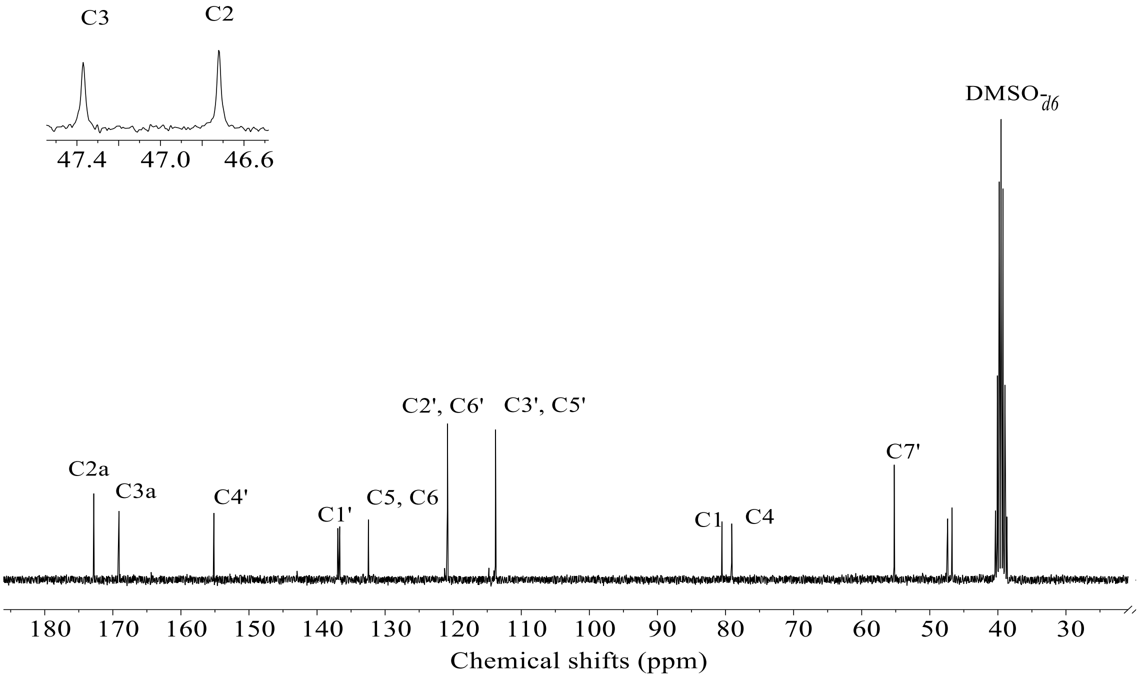

Figure S28. ^{13}C NMR (75 MHz; DMSO-d_6; δ= 40,0 ppm) of compound **7**.

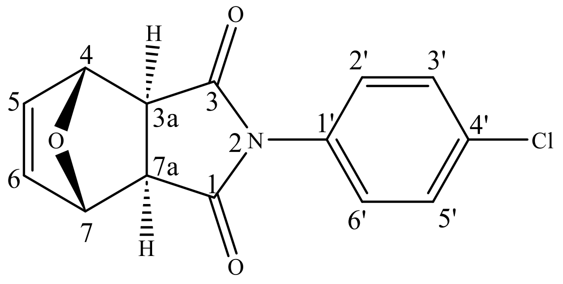

Figure S29. (3a*R*,4*S*,7*R*,7a*S*)-2-(4-chlorophenyl)-3a,4,7,7a-tetrahydro-1*H*-4,7-epoxyisoindole-1,3(2*H*)-dione (**8**).

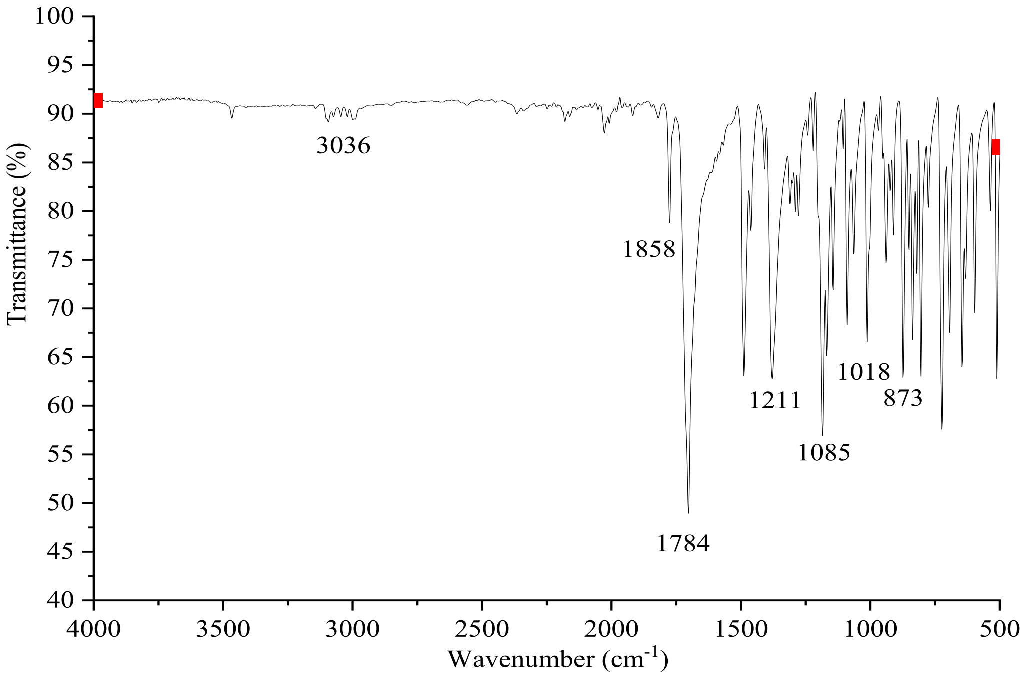

Figure S30. Infrared of compound **8** by spectrophotometer *FT-IR VARIAN 660-ATR.*

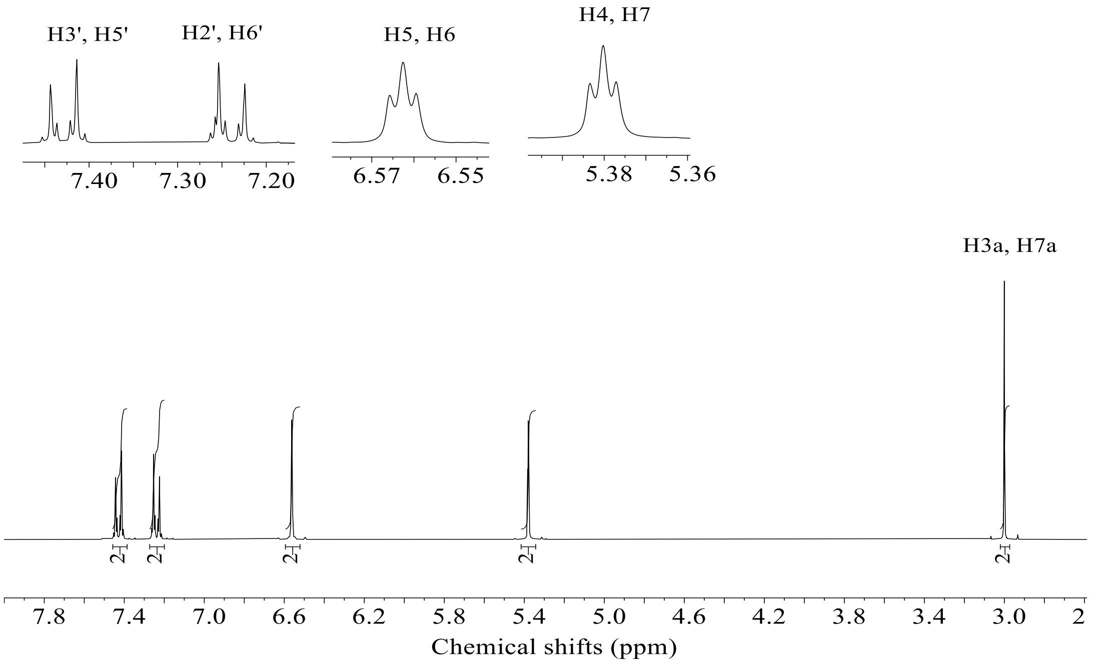

Figure S31. ^{1}H NMR (300 MHz, $CDCl_3$, δ= 7.26 ppm) of compound **8**.

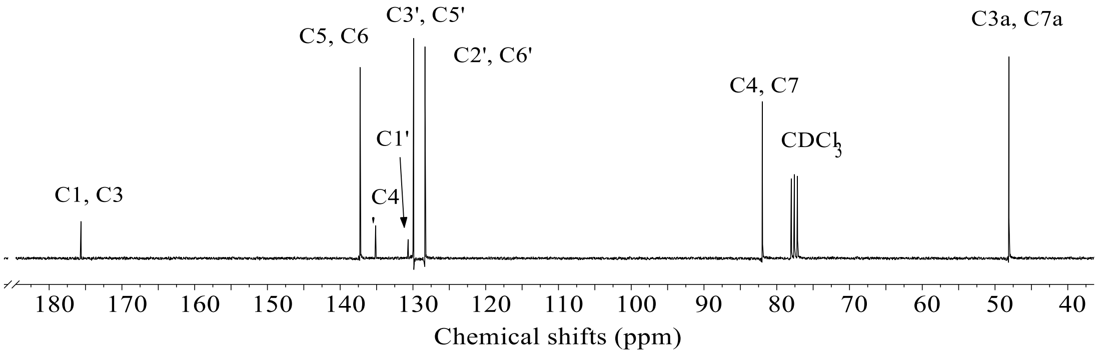

Figure S32.. ^{13}C NMR (75 MHz, $CDCl_3$, δ_{CDCl3} = 77.0 ppm) of compound **8**.

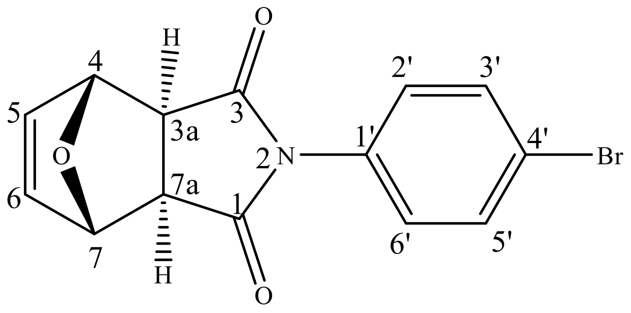

Figure S33. (3a*R*,4*S*,7*R*,7a*S*)-2-(4-bromophenyl)-3a,4,7,7a-tetrahydro-1*H*-4,7-epoxyisoindole-1,3(2*H*)-dione (**9**).

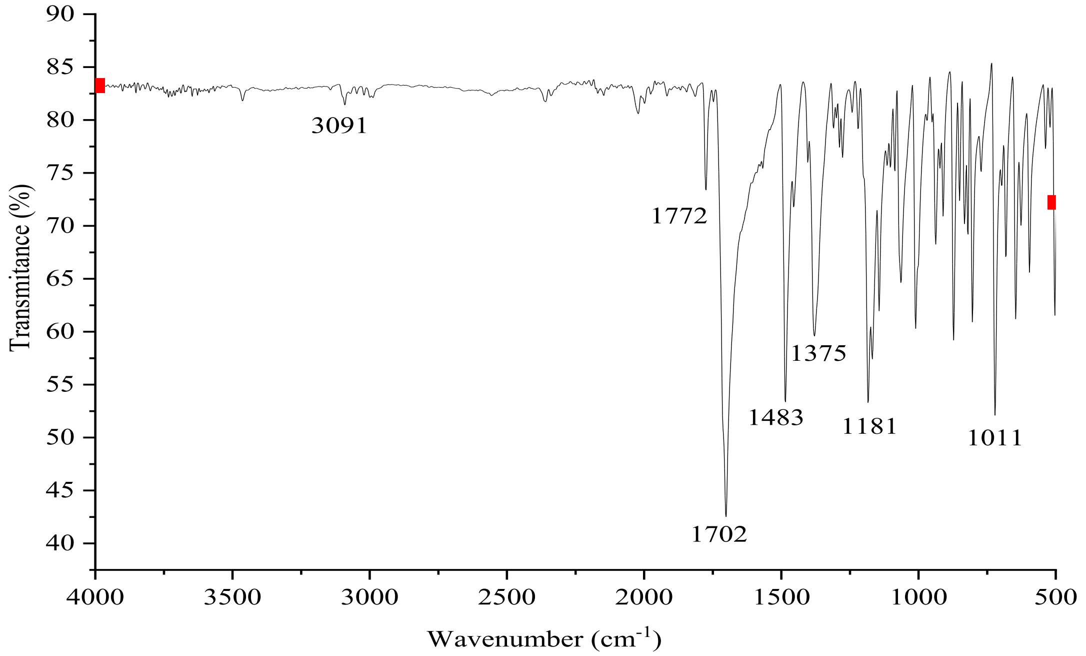

Figure S34. Infrared of compound **9** by spectrophotometer *FT-IR VARIAN 660-ATR*.

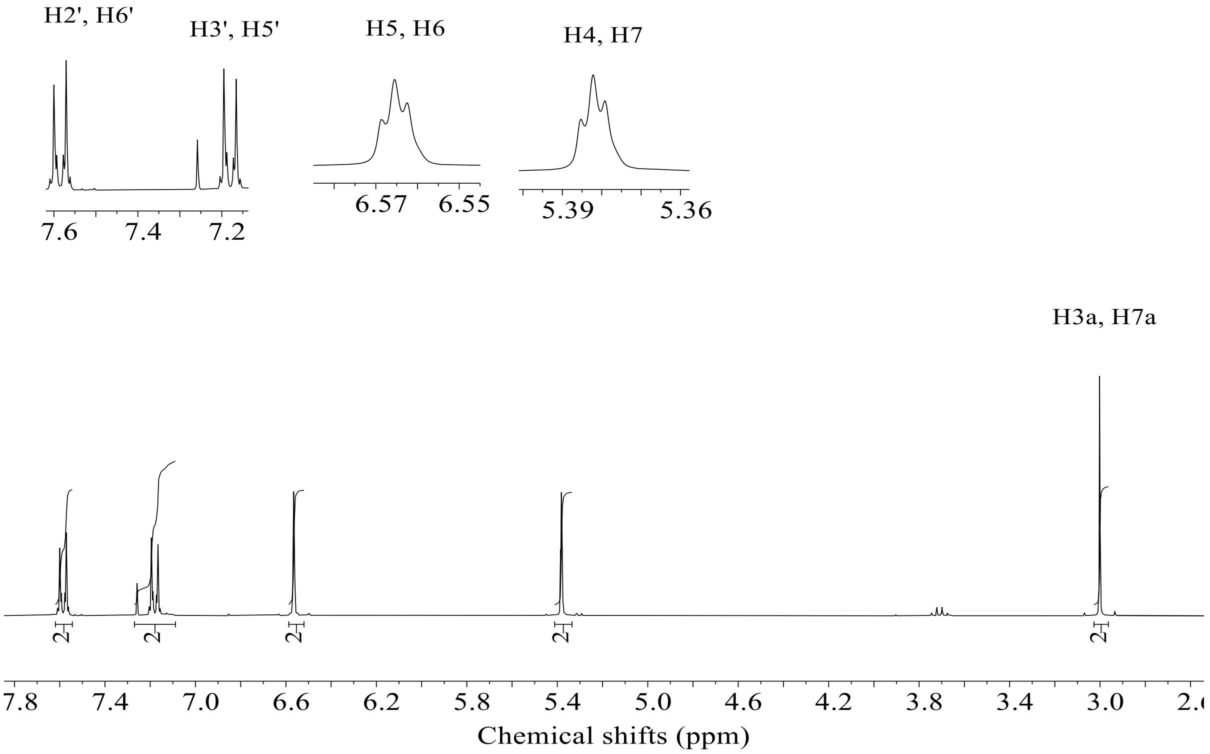

Figure S35. ^{1}H NMR (300 MHz, $CDCl_3$, δ= 7.26 ppm) of compound **9**.

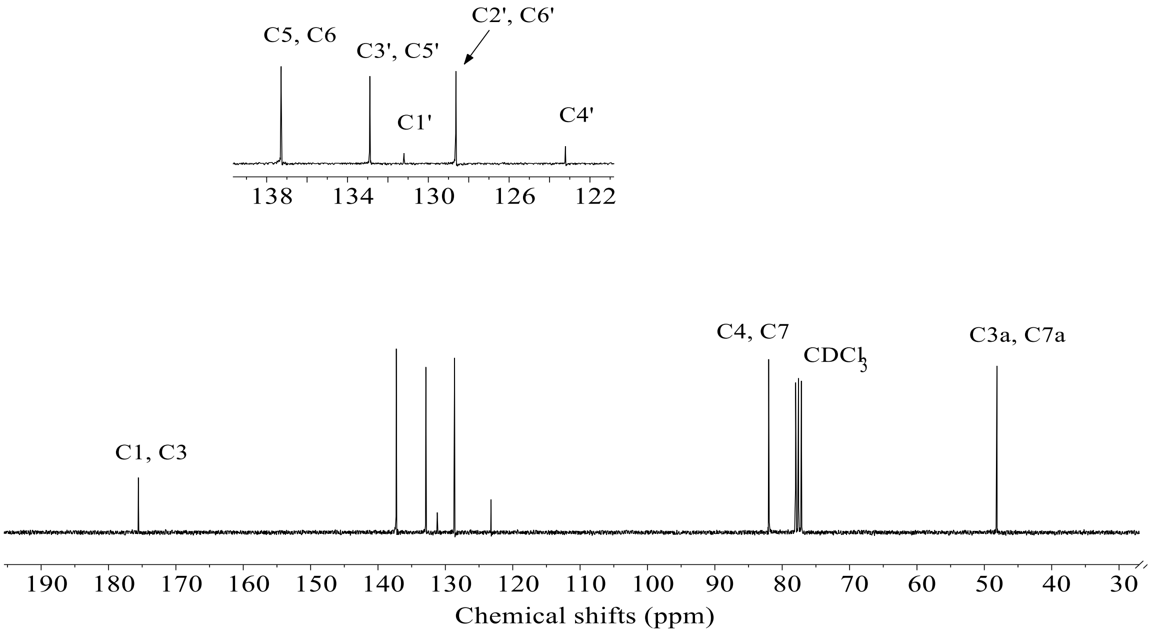

Figure S36. ^{13}C NMR (75 MHz, $CDCl_3$, δ_{CDCl3} = 77.0 ppm) of compound **9**.

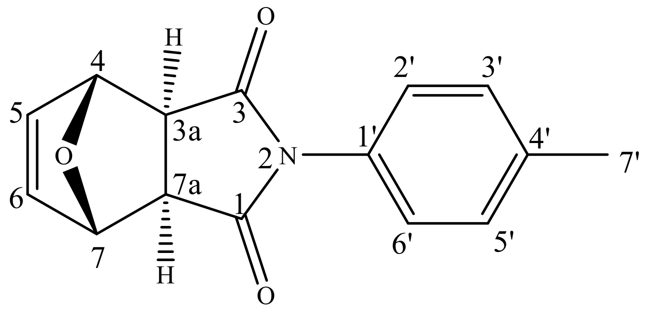

Figure S37. (3a*R*,4*S*,7*R*,7a*S*)-2-(p-tolyl)-3a,4,7,7a-tetrahydro-1*H*-4,7-epoxyisoindole-1,3(2*H*)-dione (**10**).

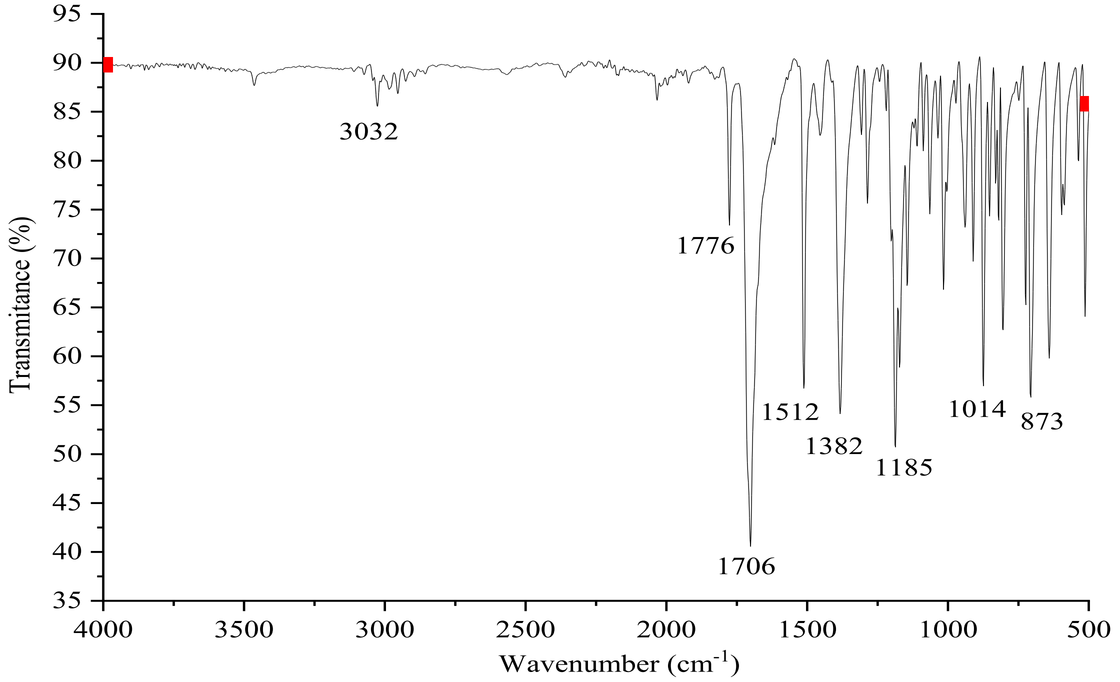

Figure S38. Infrared of compound **10** by spectrophotometer *FT-IR VARIAN 660-ATR.*

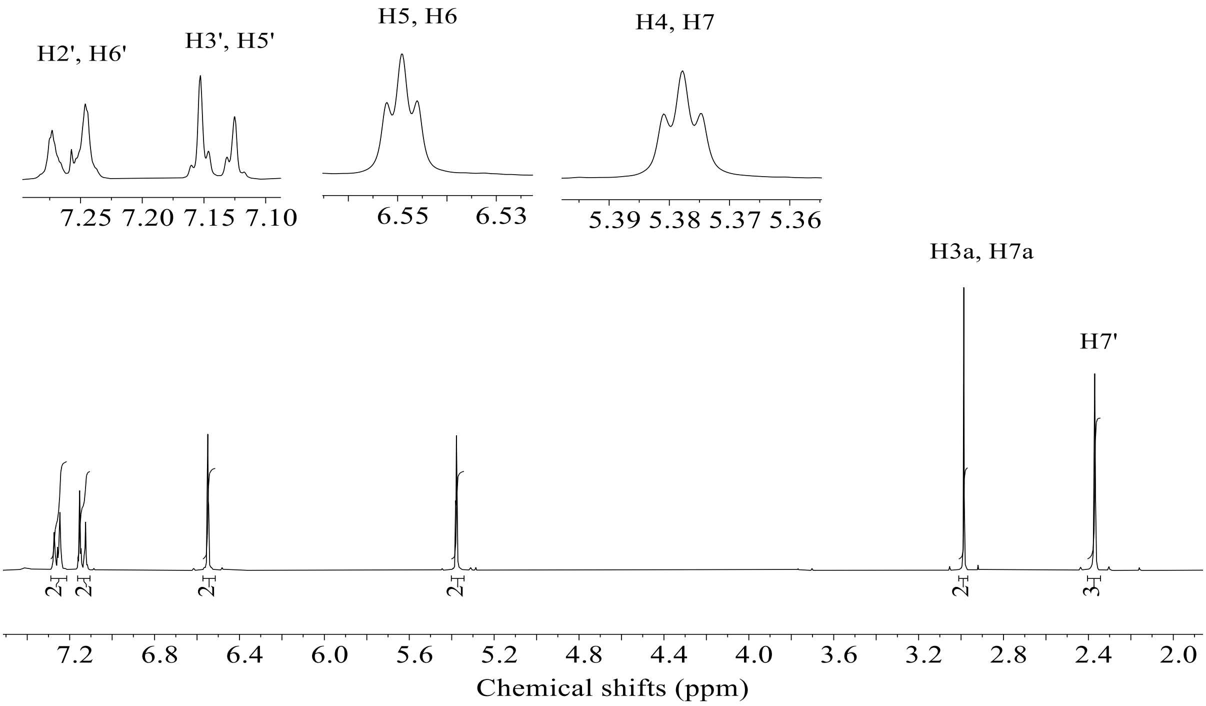

Figure S39. ¹H NMR (300 MHz, $CDCl_3$, δ= 7.26 ppm) of compound **10**.

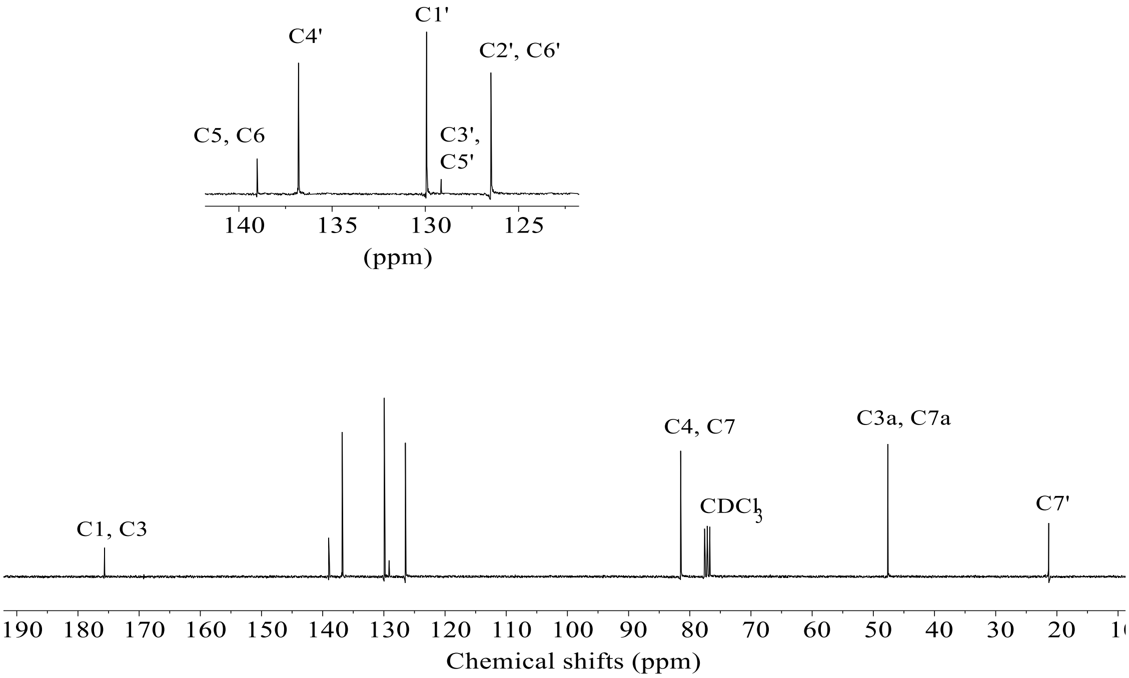

Figure S40. ¹³C NMR (75 MHz, $CDCl_3$, δ_{CDCl3} = 77.0 ppm) of compound **10**.

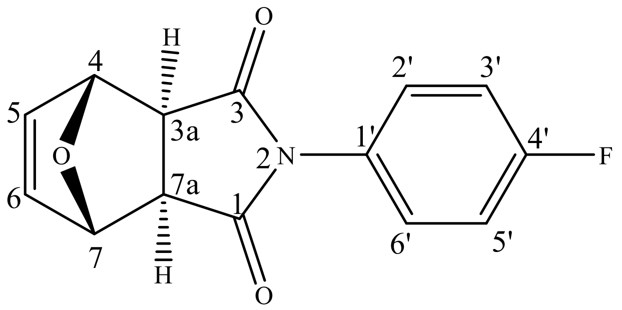

Figure S41. (3a*R*,4*S*,7*R*,7a*S*)-2-(4-fluorophenyl)-3a,4,7,7a-tetrahydro-1*H*-4,7-epoxyisoindole-1,3(2*H*)-dione (**11**).

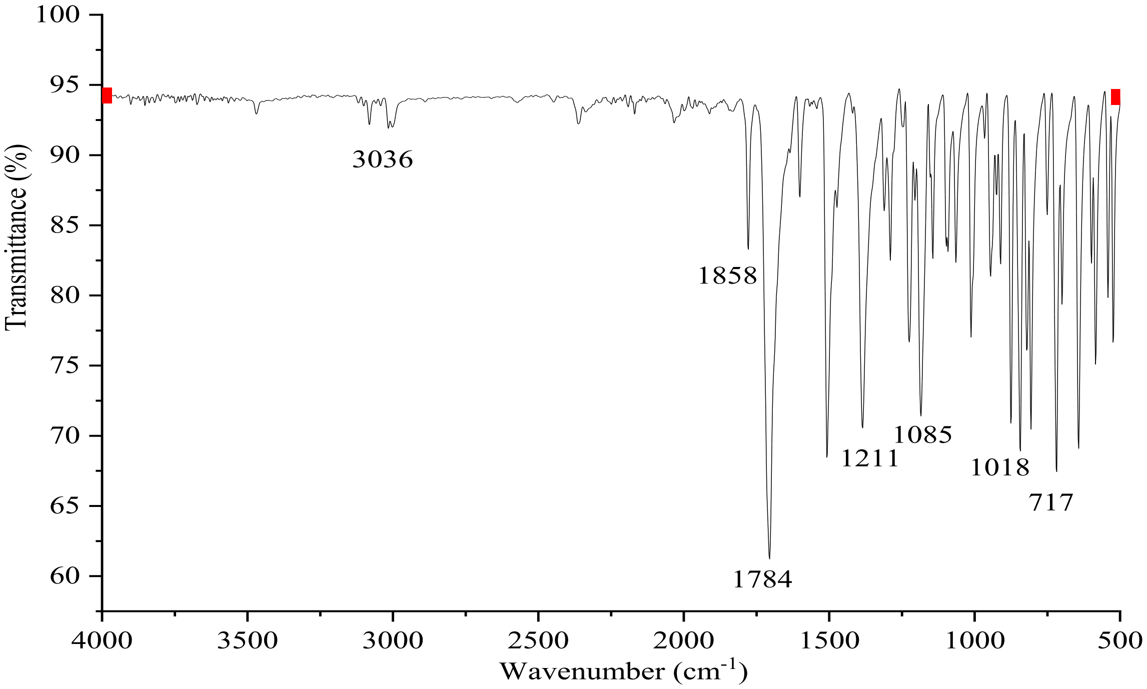

Figure S42. Infrared of compound **11** by spectrophotometer *FT-IR VARIAN 660-ATR*.

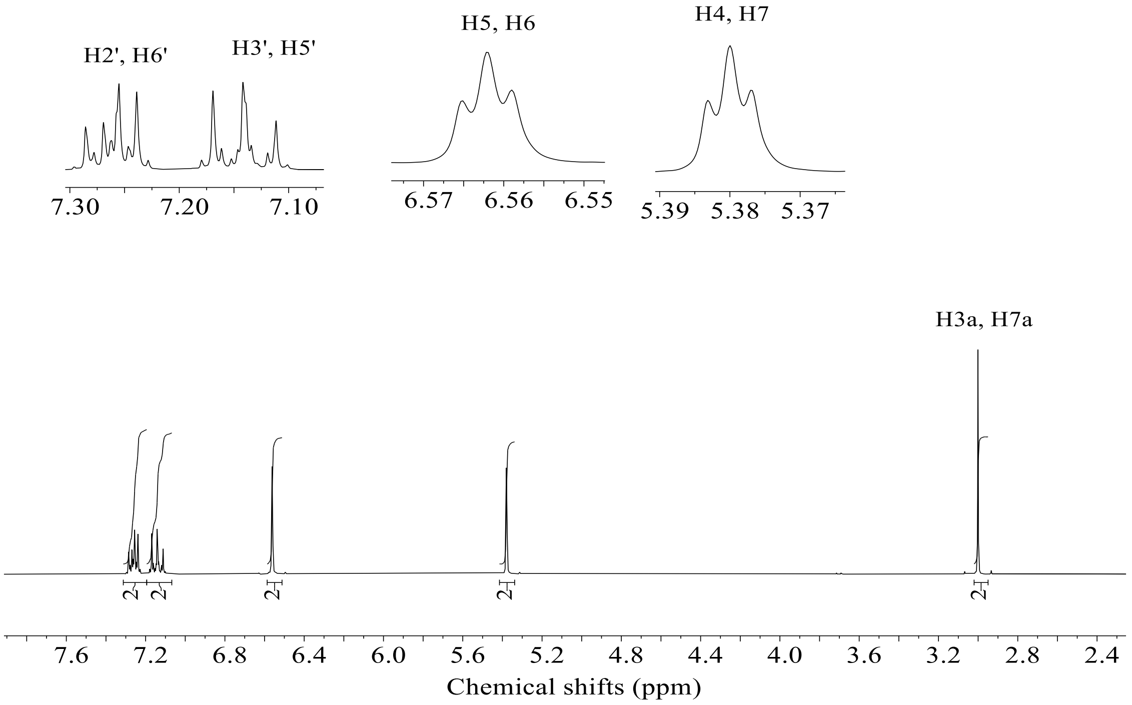

Figure S43. ^{1}H NMR (300 MHz, $CDCl_3$, δ= 7.26 ppm) of compound **11**.

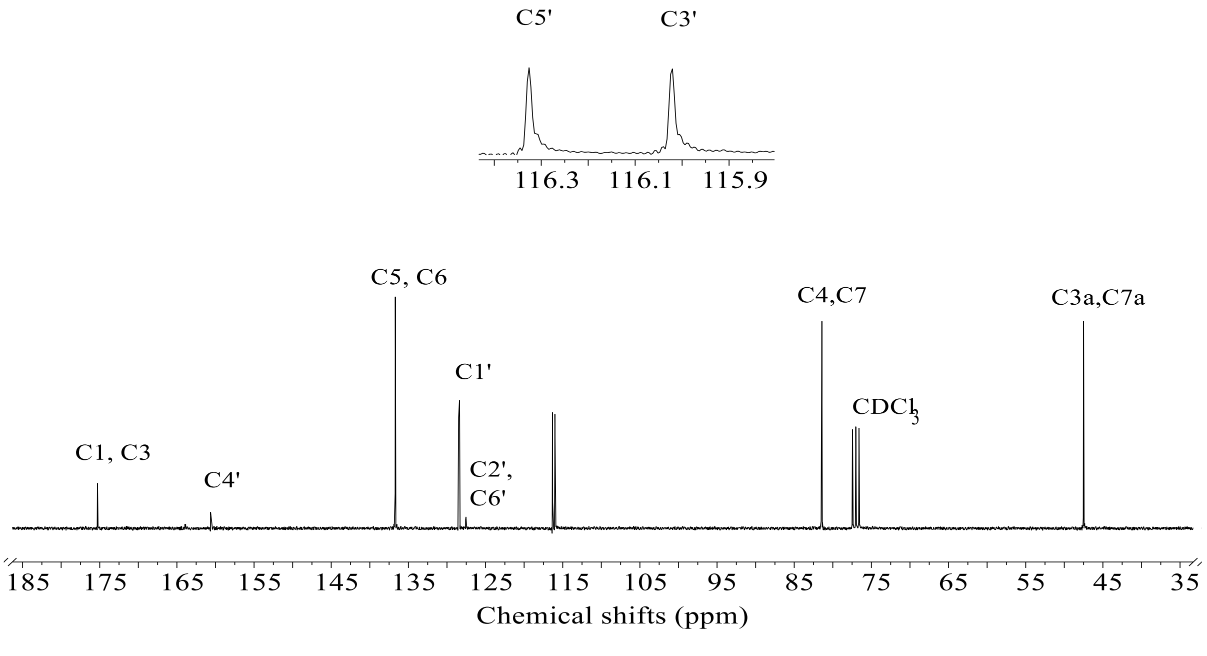

Figure S44. ^{13}C NMR (75 MHz, $CDCl_3$, δ_{CDCl3} = 77.0 ppm) of compound **11**.

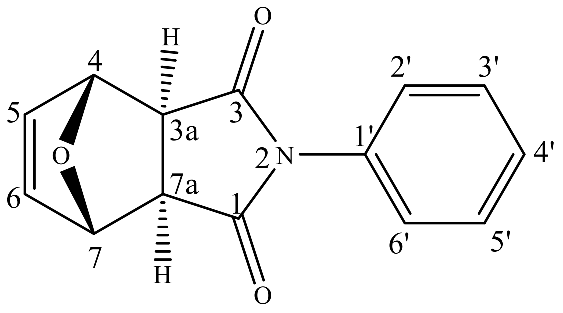

Figure S45. (3a*R*,4*S*,7*R*,7a*S*)-2-phenyl-3a,4,7,7a-tetrahydro-1*H*-4,7-epoxyisoindole-1,3(2*H*)-dione (**12**).

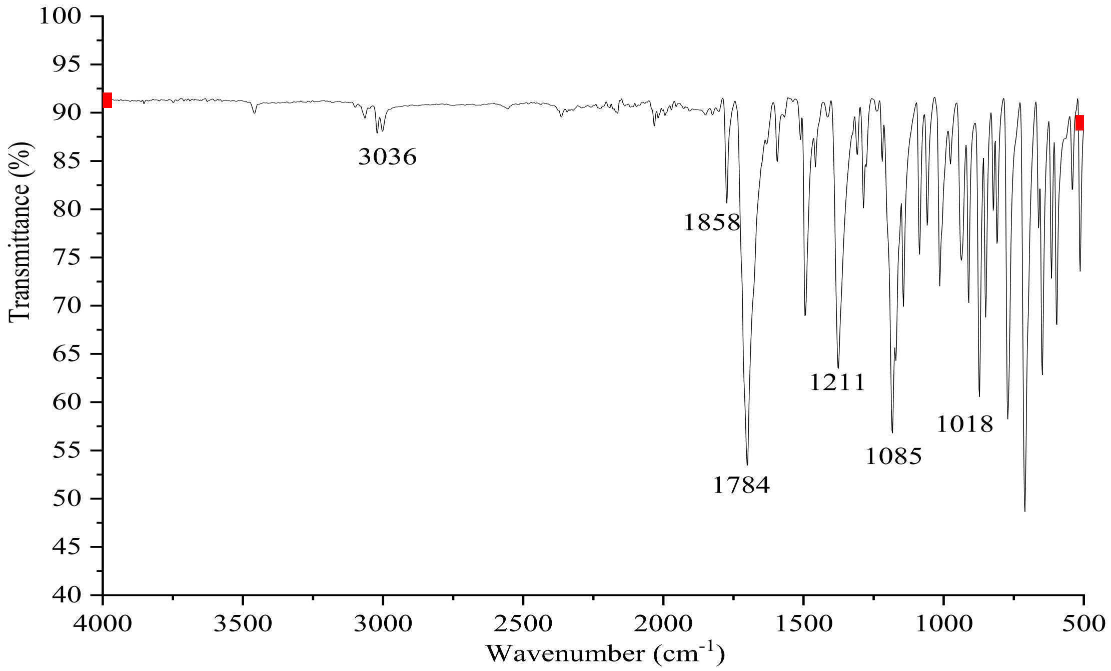

Figure S46. Infrared of compound **12** by spectrophotometer *FT-IR VARIAN 660-ATR*.

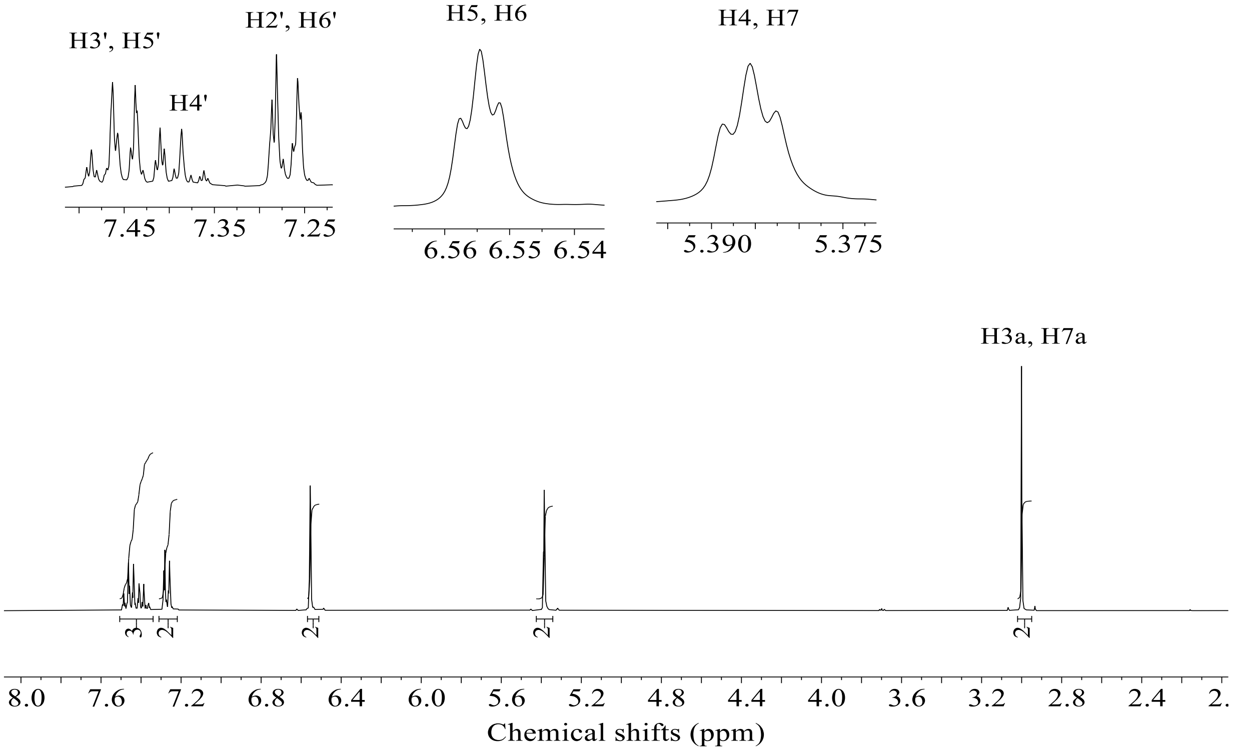

Figure S47. ^{1}H NMR (300 MHz, $CDCl_3$, δ_{CDCl3} = 7.26 ppm) of compound **12**.

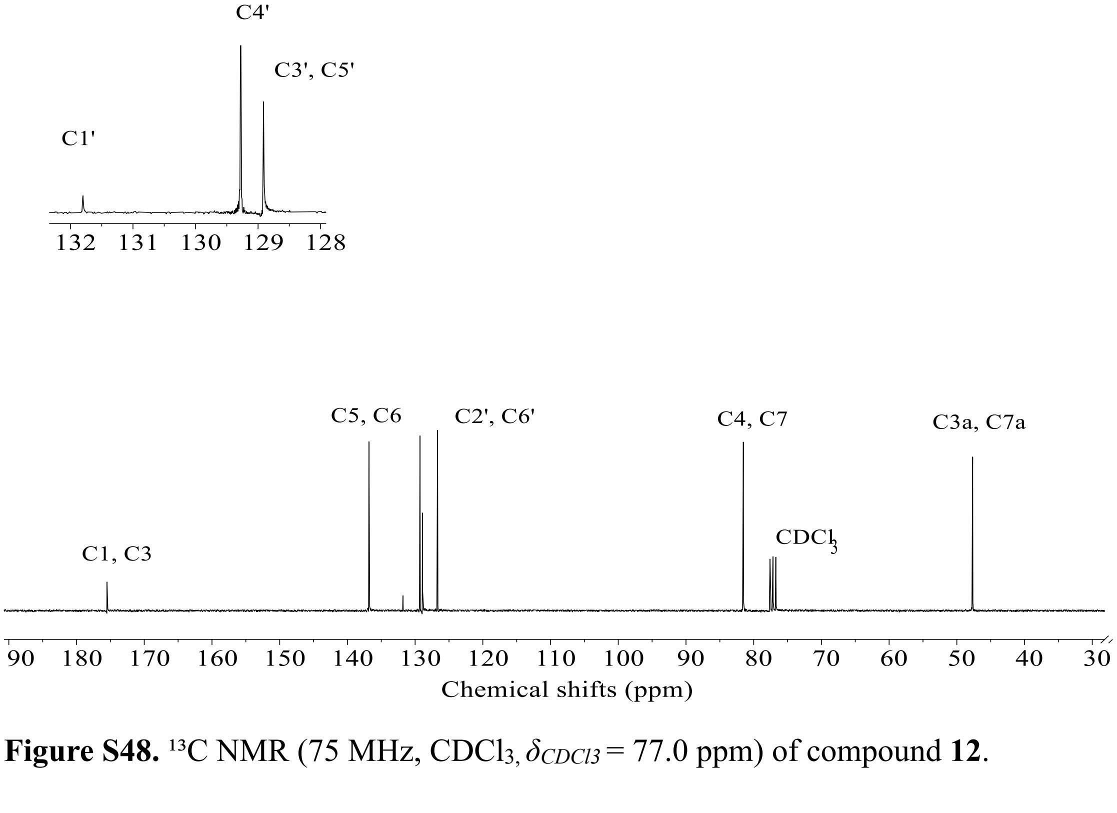

Figure S48. ^{13}C NMR (75 MHz, $CDCl_3$, δ_{CDCl3} = 77.0 ppm) of compound **12**.

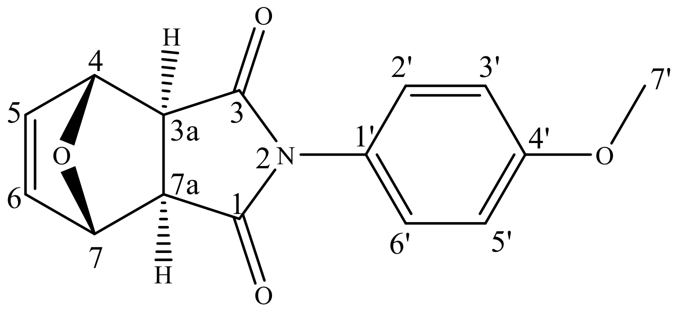

Figure S49. (3a*R*,4*S*,7*R*,7a*S*)-2-(4-methoxyphenyl)-3a,4,7,7a-tetrahydro-1*H*-4,7-epoxyisoindole-1,3(2*H*)-dione (**13**).

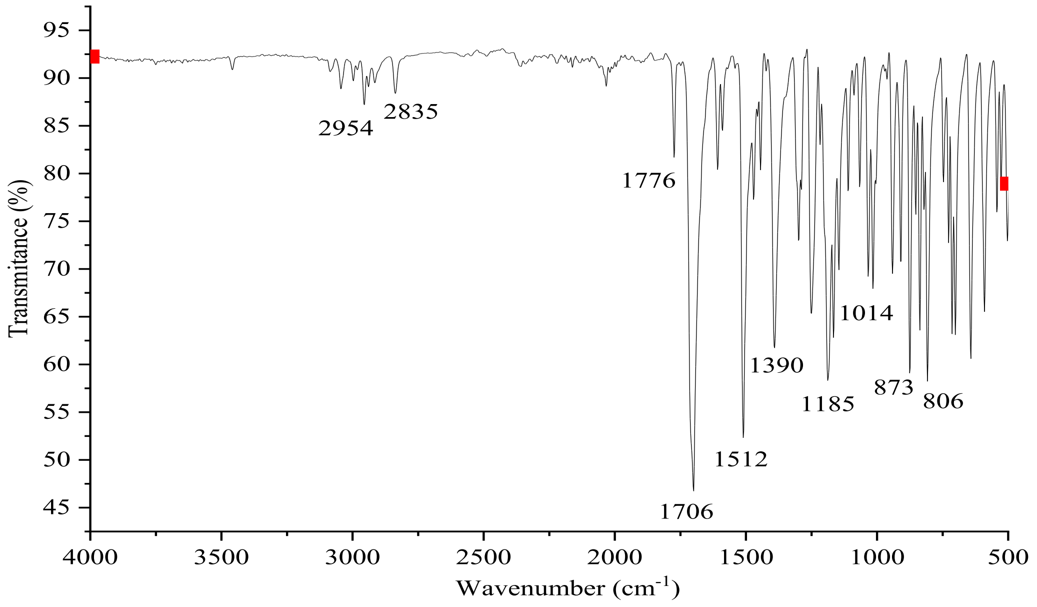

Figure S50. Infrared of compound **13** by spectrophotometer *FT-IR VARIAN 660-ATR*.

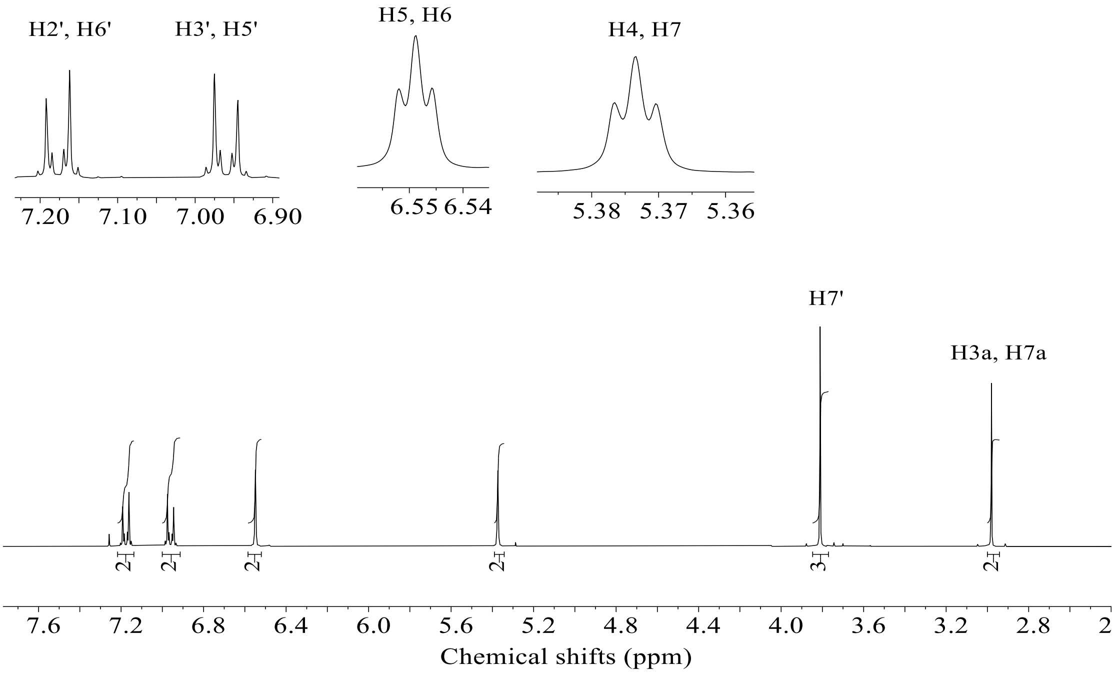

Figure S51. ^{1}H NMR (300 MHz, $CDCl_3$, δ_{CDCl3} = 7.26 ppm) of compound **13**.

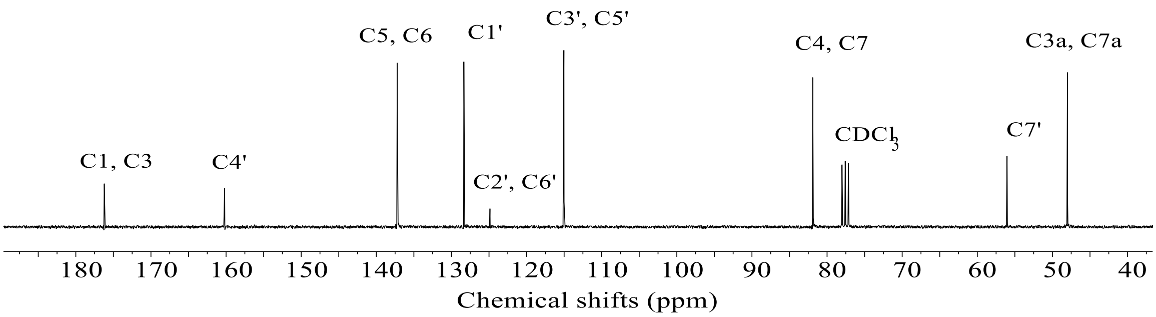

Figure S52. ^{13}C NMR (75 MHz, $CDCl_3$, δ_{CDCl3} = 77.0 ppm) of compound **13**.

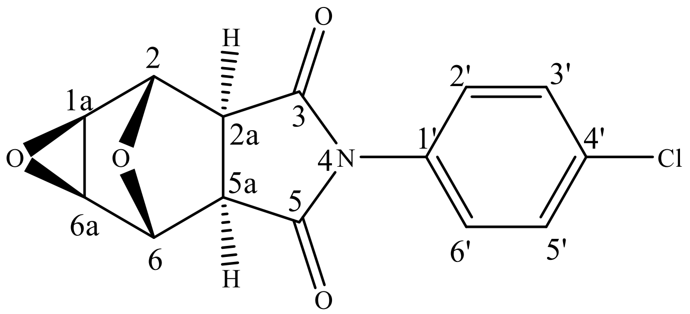

Figure S53. (1a*R*,2*R*,2a*R*,5a*S*,6*S*,6a*S*)-4-(4-chlorophenyl)hexahydro-3*H*-2,6-epoxyoxireno[2,3-*f*]isoindole-3,5(4*H*)-dione (**14**).

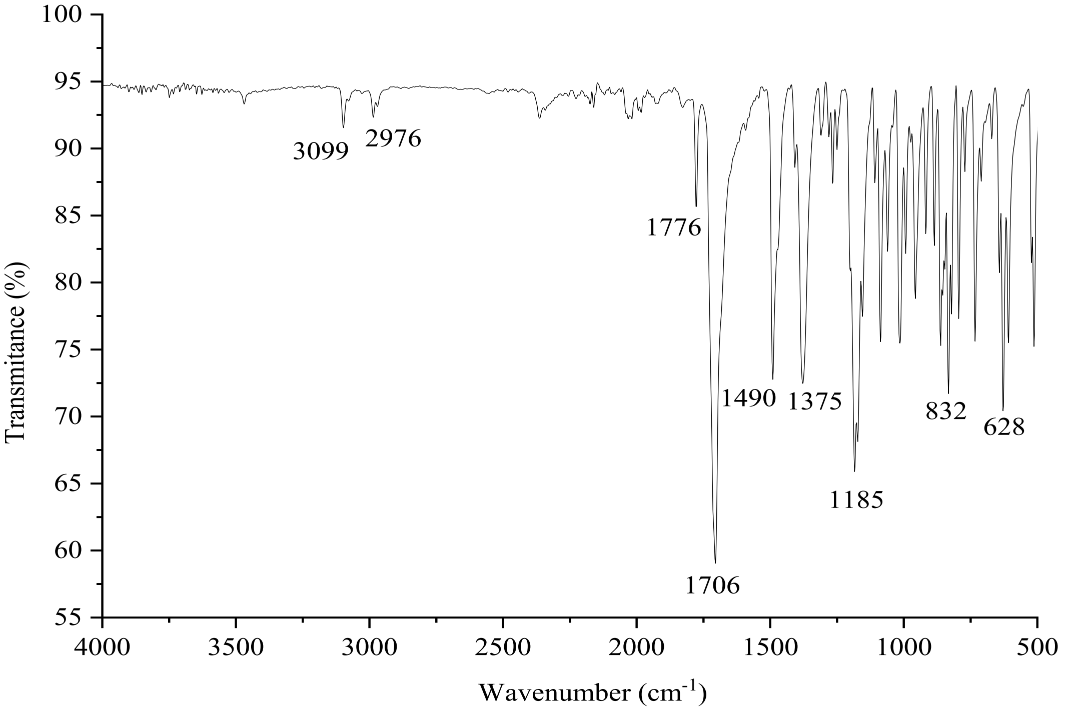

Figure S54. Infrared of compound **14** by spectrophotometer *FT-IR VARIAN 660-ATR*.

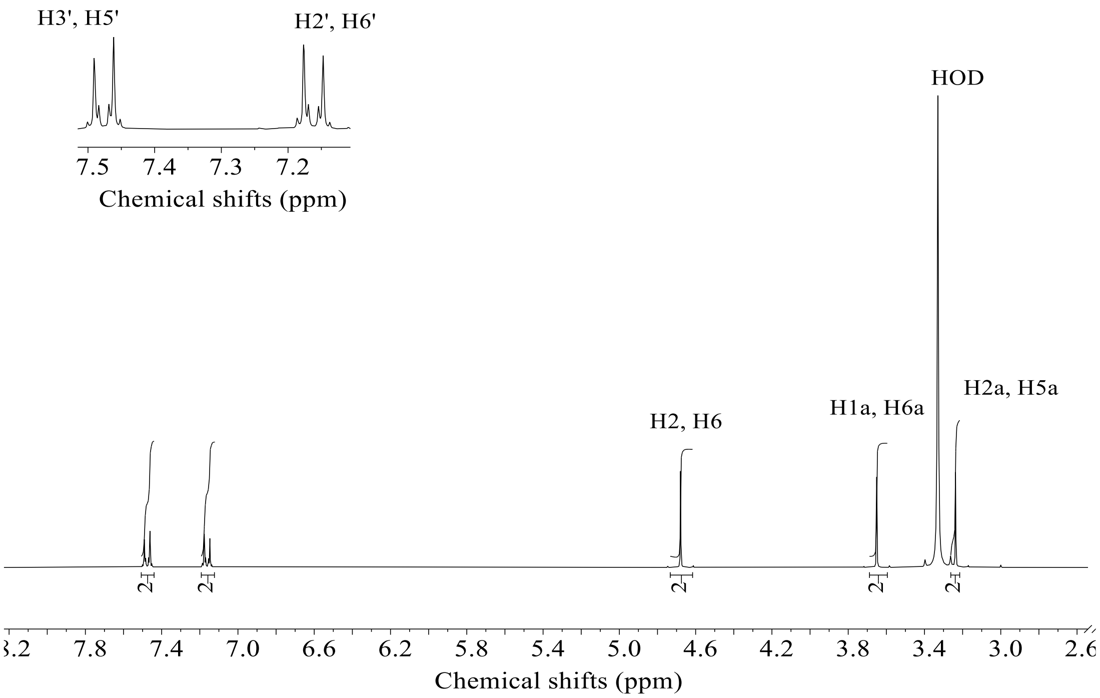

Figure S55. ^{1}H NMR (300 MHz; DMSO-d_6; δ= 3,3 ppm) of compound **14**.

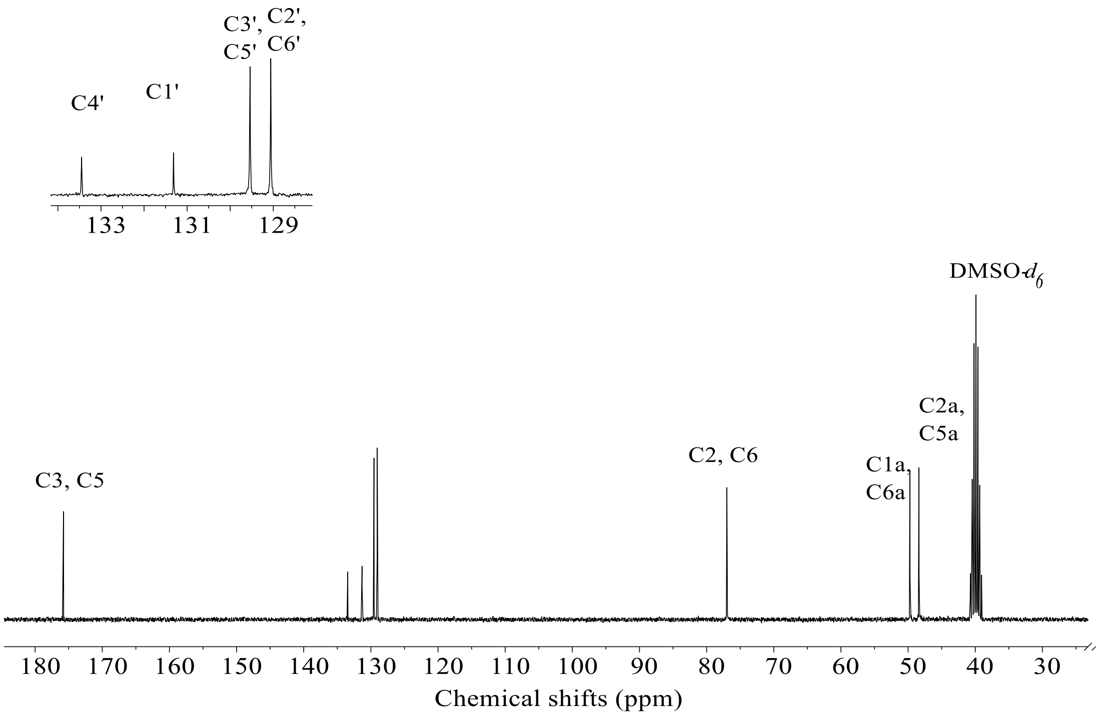

Figure 56. ^{13}C NMR (75 MHz; DMSO-d_6; δ= 40,0 ppm) of compound **14**.

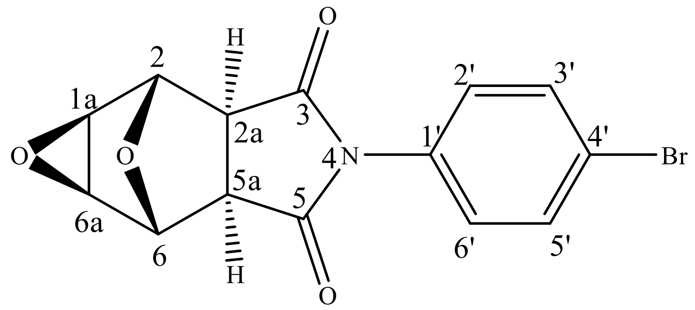

Figure S57. (1aR,2R,2aR,5aS,6S,6aS)-4-(4-bromophenyl)hexahydro-3H-2,6-epoxyoxireno[2,3-f]isoindole-3,5(4H)-dione (**15**).

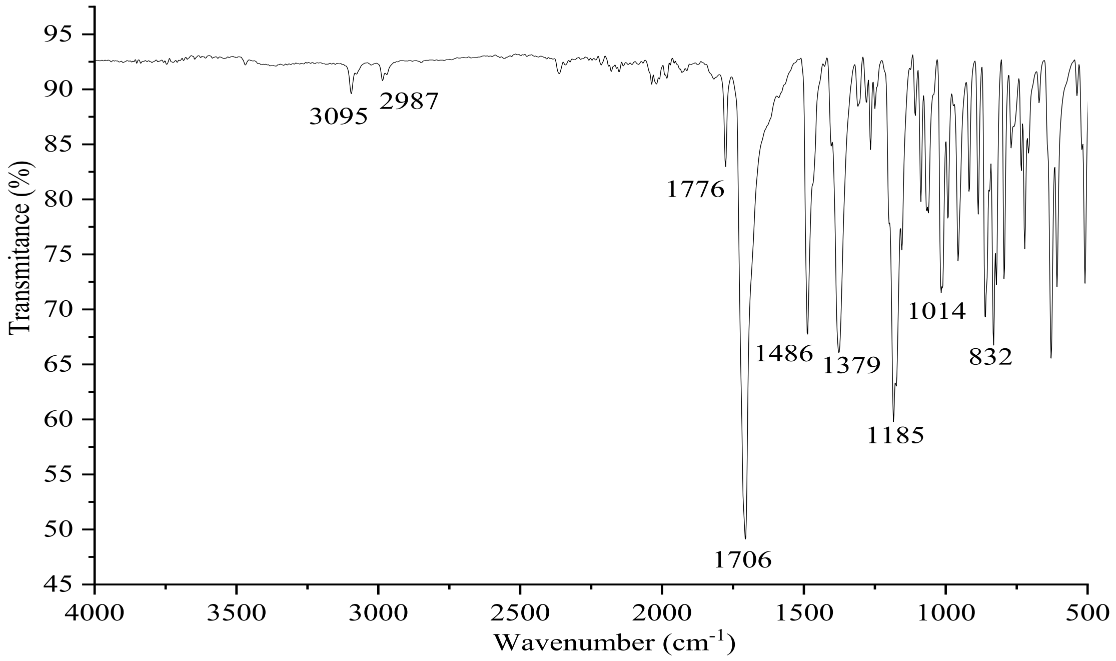

Figure S58. Infrared of compound **15** by spectrophotometer *FT-IR VARIAN 660-ATR.*

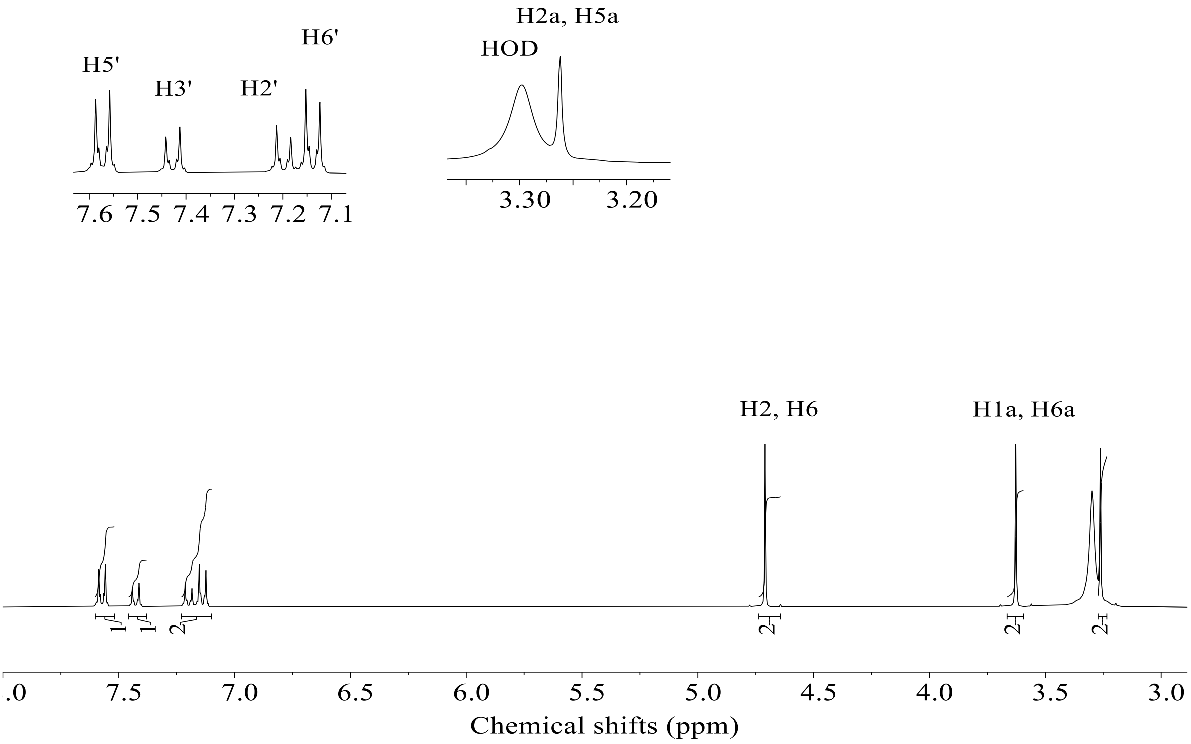

Figure S59. ^{1}H NMR (300 MHz; DMSO-d_6 and $CDCl_3$) of compound **15**.

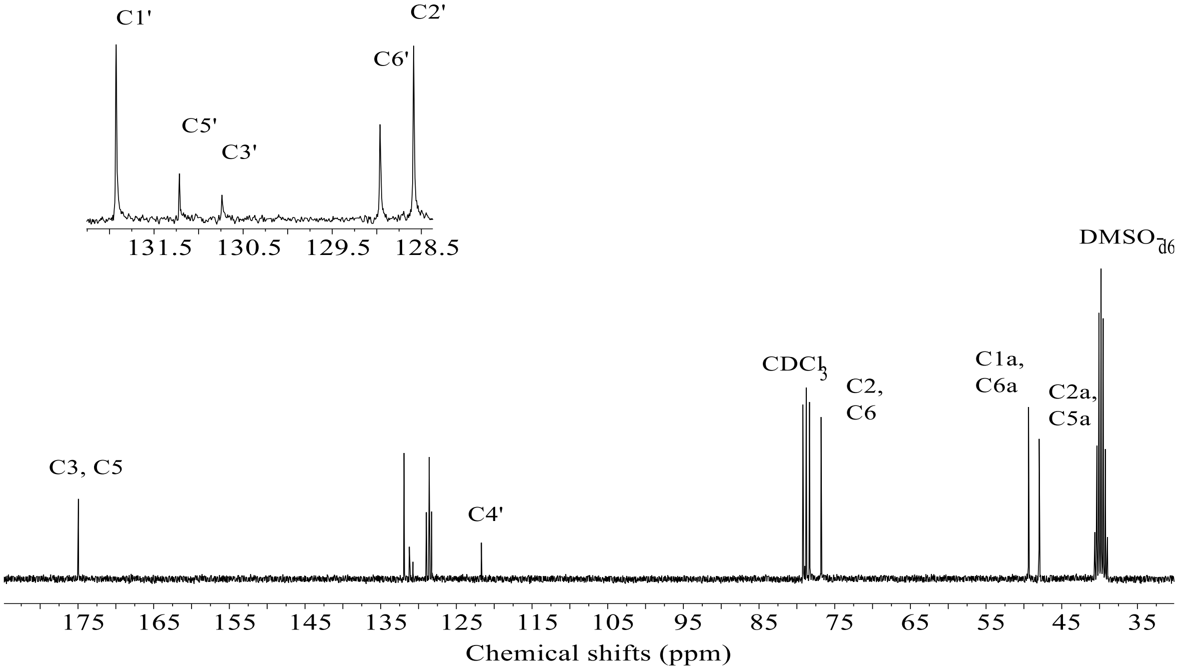

Figure S60. ^{13}C NMR (75 MHz; DMSO-d_6 and $CDCl_3$) of compound **15**.

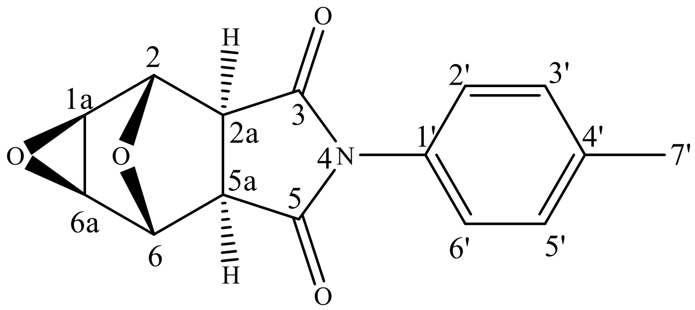

Figure S61. (1a*R*,2*R*,2a*R*,5a*S*,6*S*,6a*S*)-4-(p-tolyl)hexahydro-3*H*-2,6-epoxyoxireno[2,3-*f*]isoindole-3,5(4*H*)-dione (**16**).

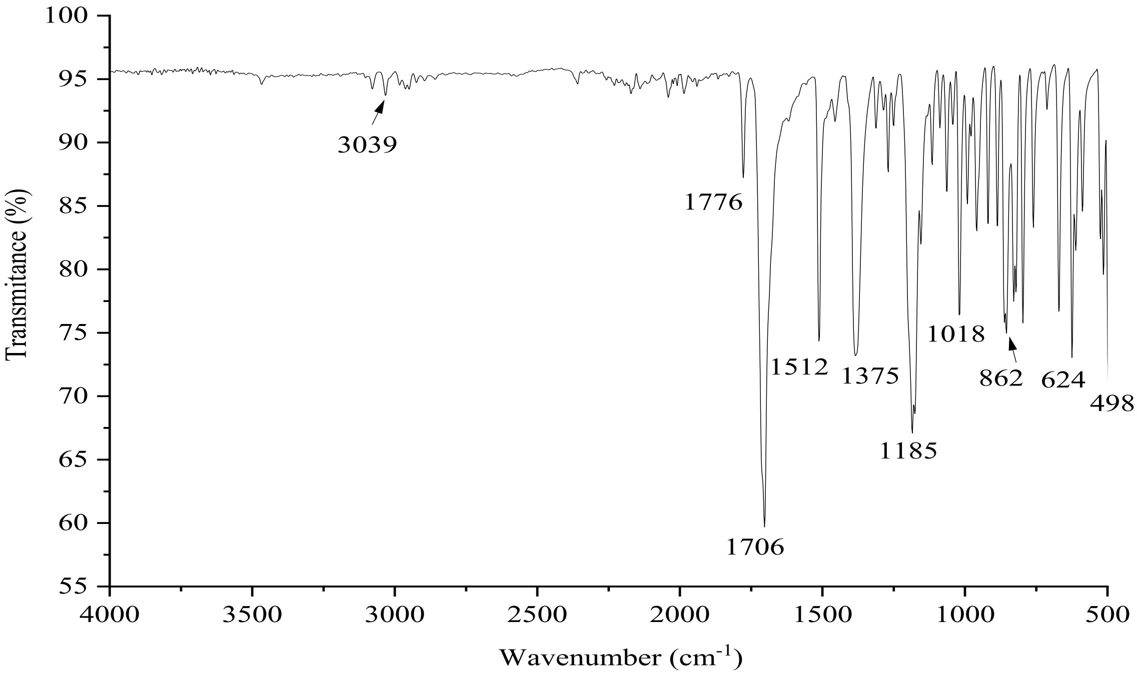

Figure S62. Infrared of compound **16** by spectrophotometer *FT-IR VARIAN 660-ATR.*

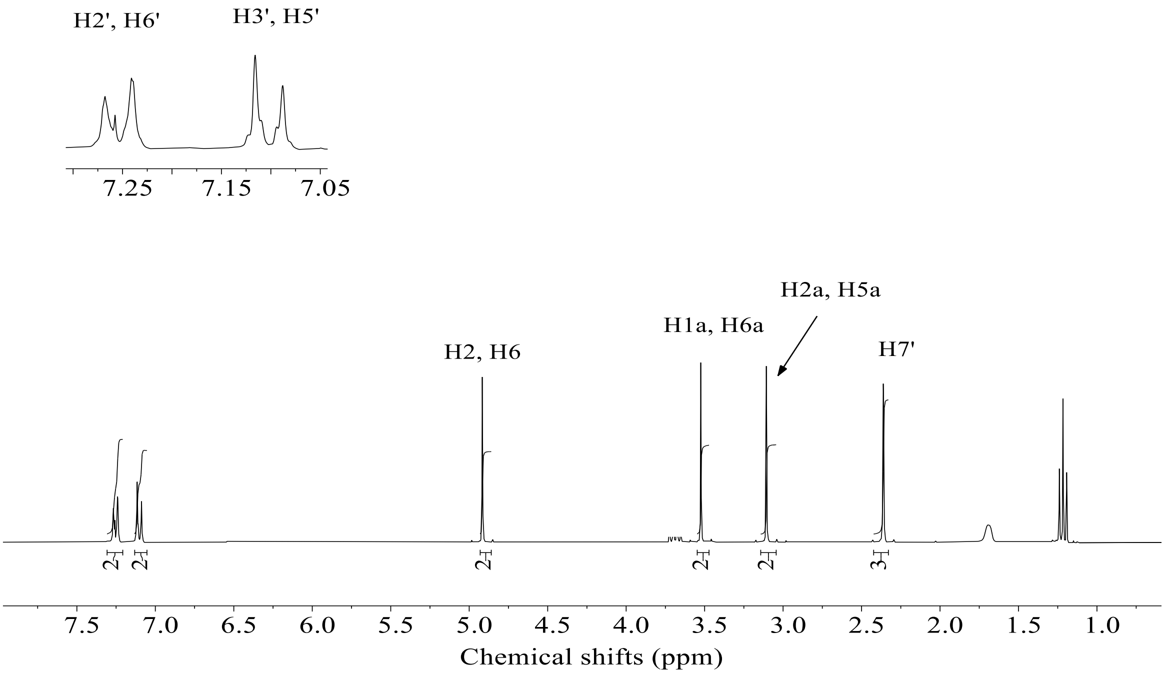

Figure S63. ^{1}H NMR (300 MHz, $CDCl_3$, δ_{CDCl3} = 7.26 ppm) of compound **16**.

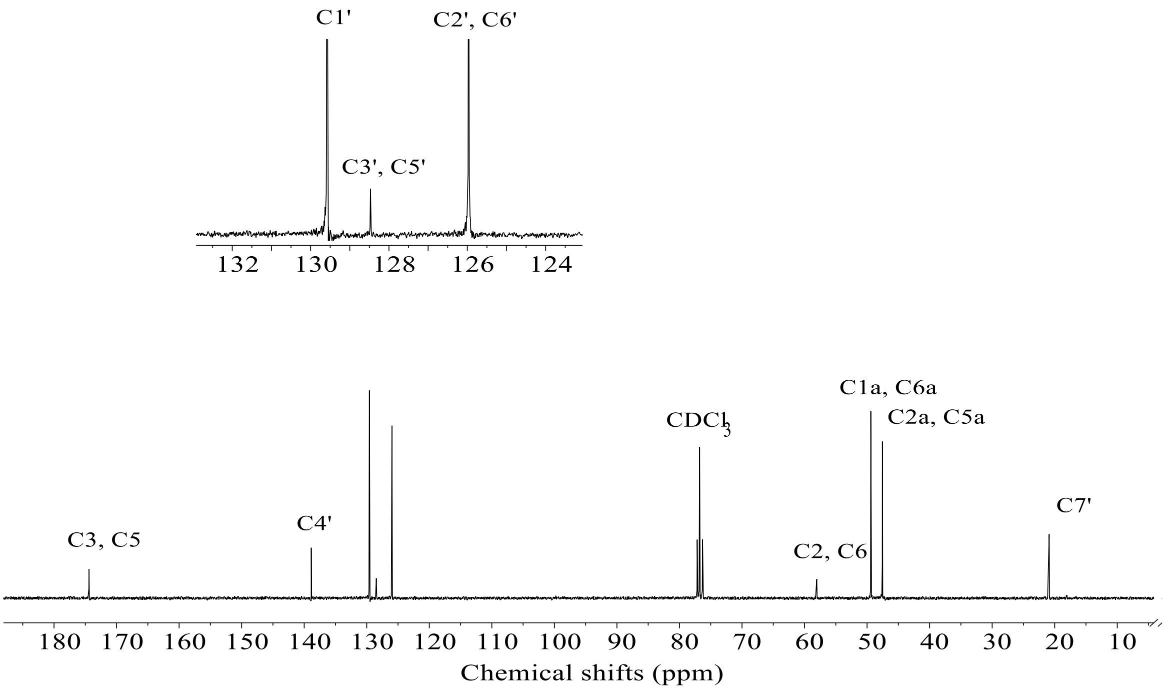

Figure S64. ^{13}C NMR (75 MHz, $CDCl_3$, δ_{CDCl3} = 77.0 ppm) of compound **16**.

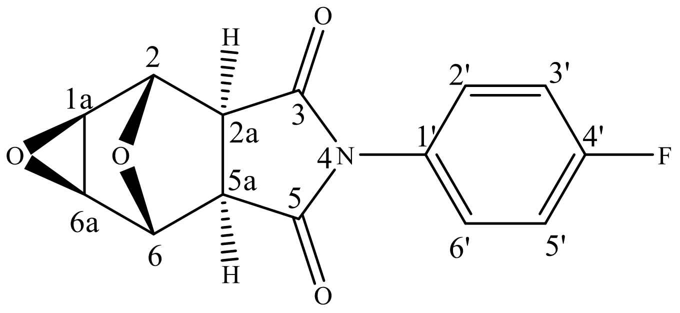

Figure S65. (1a*R*,2*R*,2a*R*,5a*S*,6*S*,6a*S*)-4-(4-fluorophenyl)hexahydro-3*H*-2,6-epoxyoxireno[2,3-*f*]isoindole-3,5(4*H*)-dione (**17**).

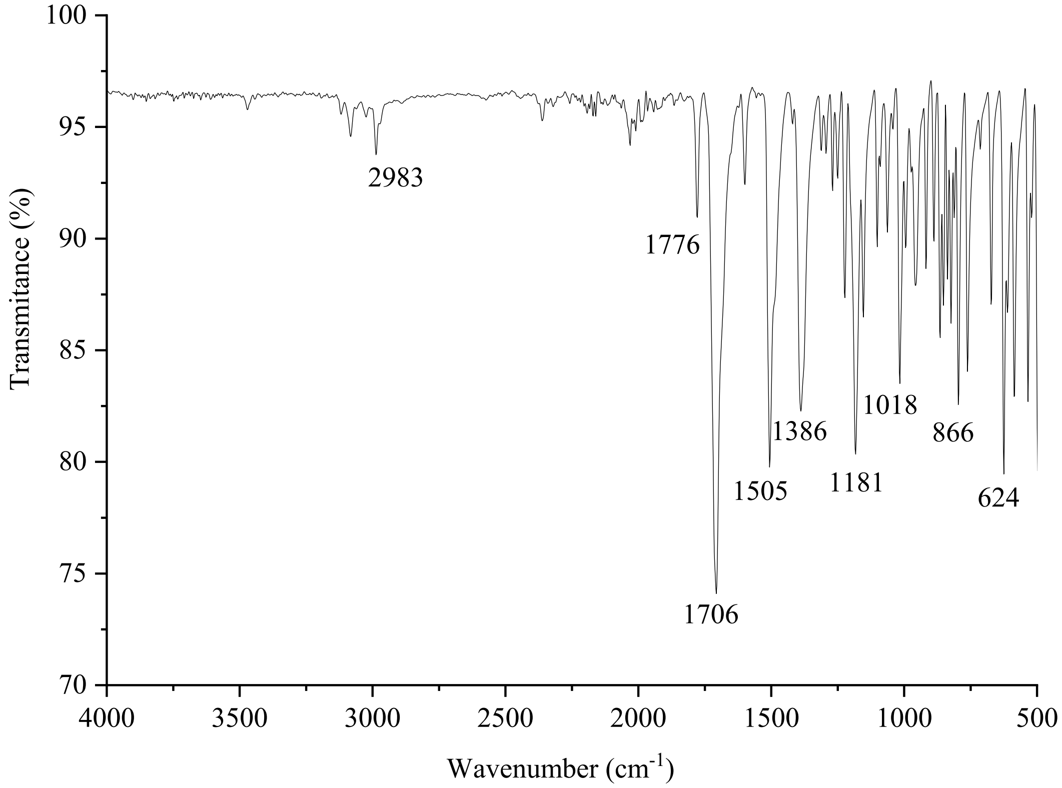

Figure S66. Infrared of compound **17** by spectrophotometer *FT-IR VARIAN 660-ATR*.

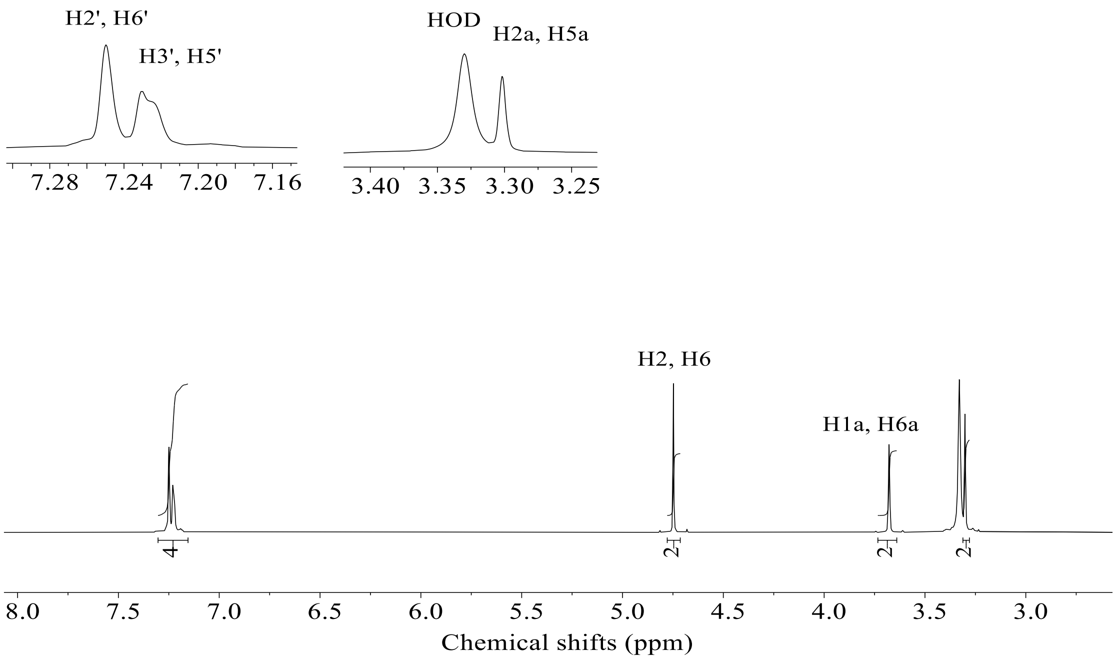

Figure S67. ^{1}H NMR (300 MHz, DMSO-d_6 and $CDCl_3$) of compound **17**.

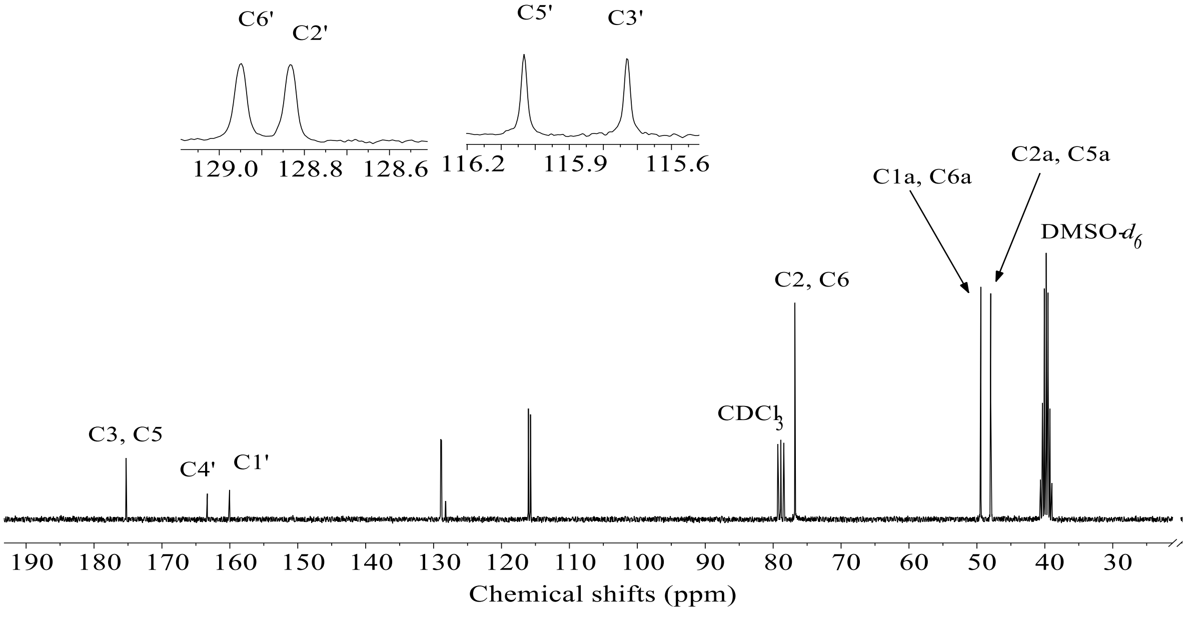

Figure S68. ^{13}C NMR (75 MHz; DMSO-d_6 and $CDCl_3$) of compound **17**.

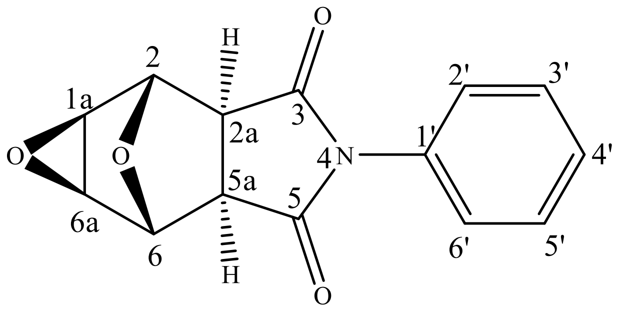

Figure S69. (1a*R*,2*R*,2a*R*,5a*S*,6*S*,6a*S*)-4-phenylhexahydro-3*H*-2,6-epoxyoxireno[2,3-*f*]isoindole-3,5(4*H*)-dione (**18**).

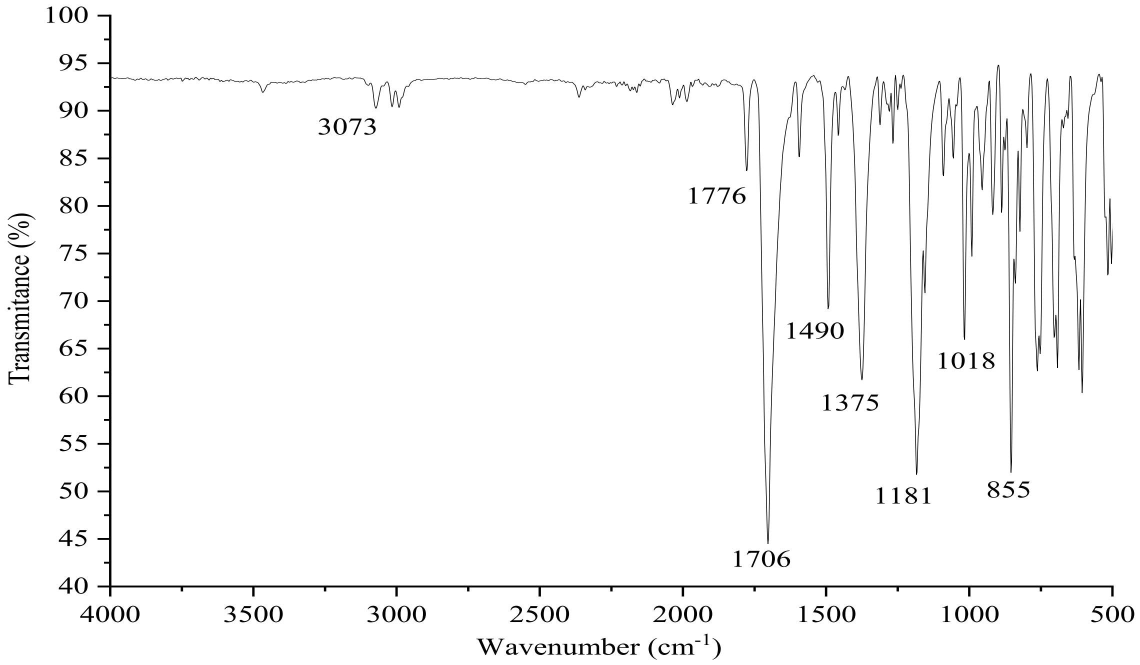

Figure S70. Infrared of compound **18** by spectrophotometer *FT-IR VARIAN 660-ATR.*

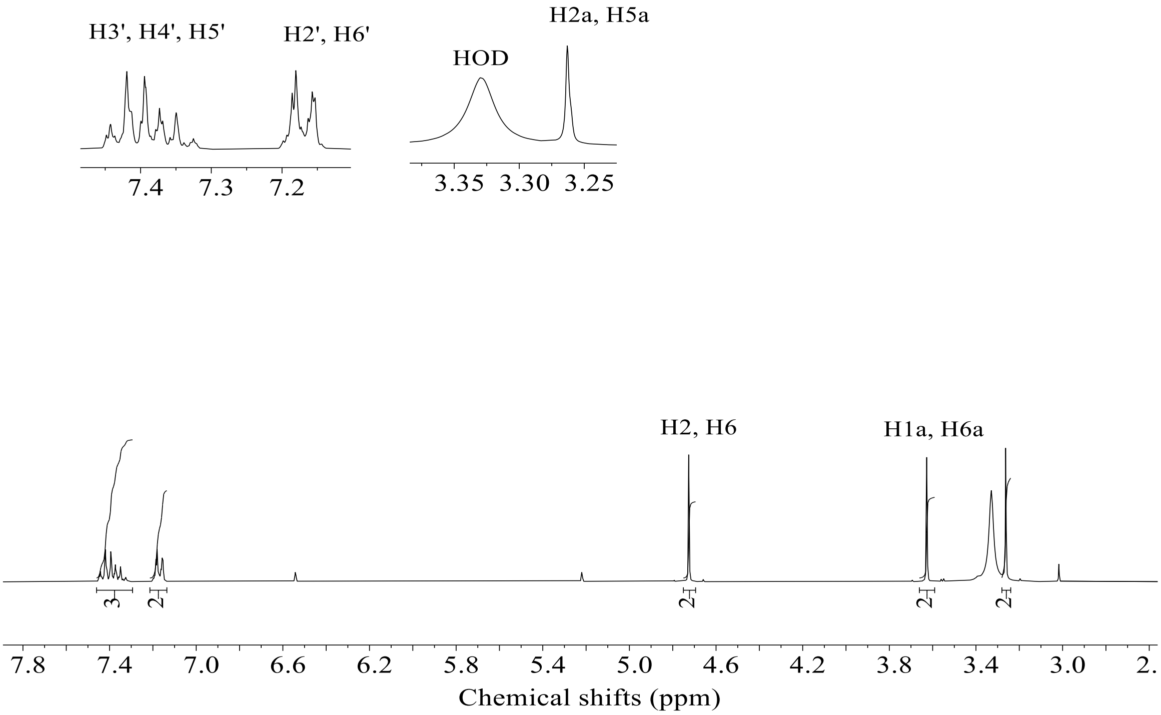

Figure S71. ^{1}H NMR (300 MHz, DMSO-d_6 and $CDCl_3$) of compound **18**.

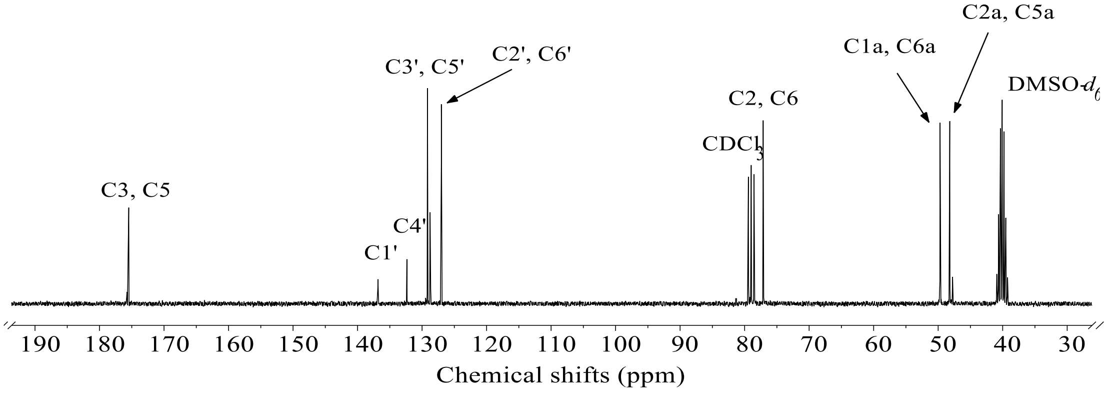

Figure S72. ^{13}C NMR (75 MHz; DMSO-d_6 and $CDCl_3$) of compound **18**.

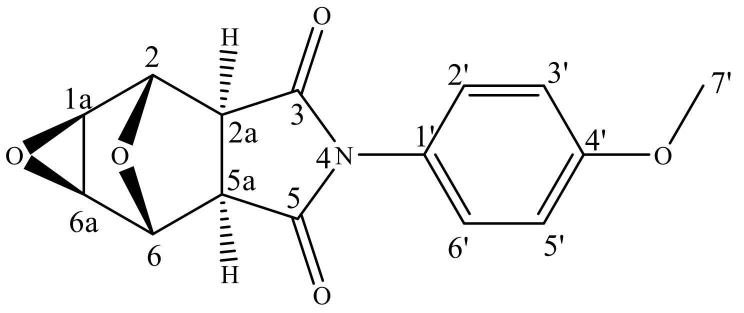

Figure S73. (1a*R*,2*R*,2a*R*,5a*S*,6*S*,6a*S*)-4-(4-methoxyphenyl)hexahydro-3*H*-2,6-epoxyoxireno[2,3-*f*]isoindole-3,5(4*H*)-dione (**19**).

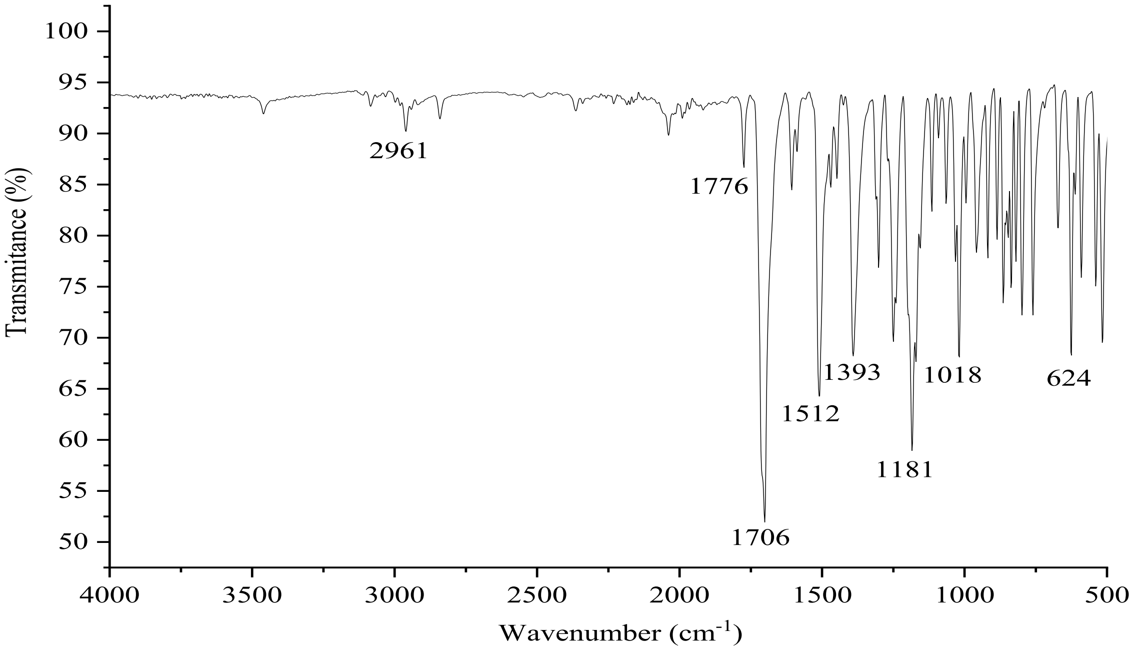

Figure S74. Infrared of compound **19** by spectrophotometer *FT-IR VARIAN 660-ATR*.

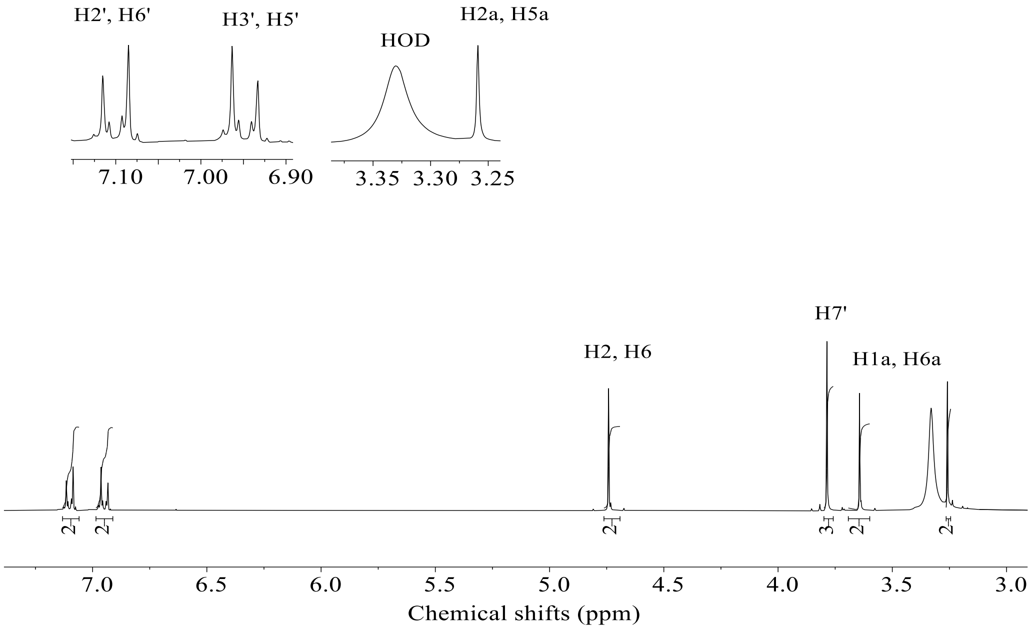

Figure S75. ^{1}H NMR (300 MHz, DMSO-d_6 and $CDCl_3$) of compound **19**.

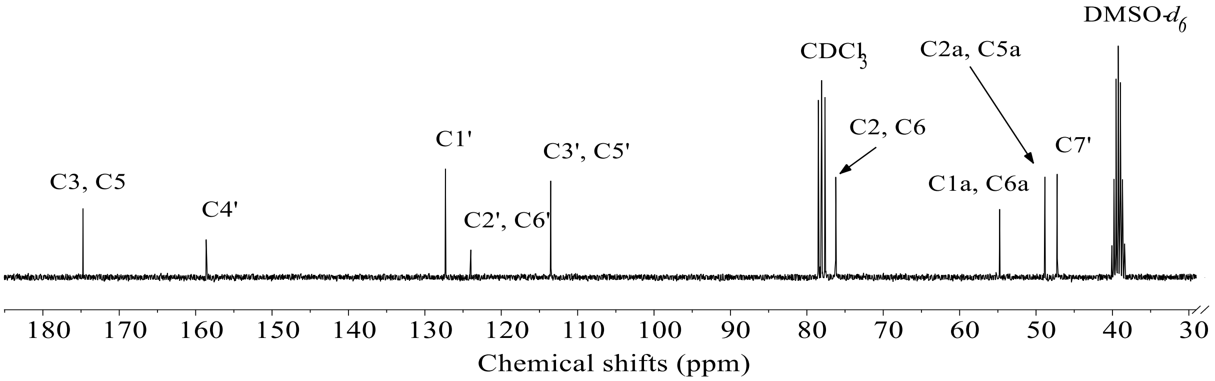

Figure S76. ^{13}C NMR (75 MHz; DMSO-d_6 and $CDCl_3$) of compound **19**.

Table S1. Root inhibition rate of compounds **2-19** against *Bidens pilosa* seedlings.

**Root length**

Compounds	50 μM	125 μM	250 μM	500 μM	1000 μM
**DUAL**	-44,76	-64,83	-60,86	-10,56	-44,76
**2**	-100,00	-100,00	-100,00	-100,00	-100,00
**3**	-100,00	-100,00	-100,00	-100,00	-100,00
**4**	-100,00	-90,95	-88,81	-100,00	-100,00
**5**	-100,00	-90,95	-90,00	-100,00	-100,00
**6**	-100,00	-90,95	-100,00	-100,00	-100,00
**7**	-100,00	-94,76	-93,81	-100,00	-100,00
**8**	-36,43	-28,69	-39,68	-53,21	-36,43
**9**	-65,87	-12,04	-46,41	-10,83	-65,87
**10**	-20,00	-58,48	-66,35	-93,45	-20,00
**11**	-4,76	-43,36	-100,00	-74,29	-4,76
**12**	-20,00	-38,33	-39,81	-67,05	-20,00
**13**	-100,00	-57,38	-66,67	-100,00	-100,00
**14**	-85,24	-36,29	-50,00	-90,00	-85,24
**15**	-21,90	-34,29	-34,76	-100,00	-21,90
**16**	-100,00	-11,90	-100,00	-100,00	-100,00
**17**	-72,86	-16,31	-8,76	-33,95	-72,86
**18**	-95,24	-100,00	-100,00	-56,90	-95,24
**19**	-32,38	-40,16	-24,95	-26,43	-32,38

Table S2. Shoot inhibition rate of compounds **2-19** against *Bidens pilosa* seedlings.

			**Shoot length**		
**Compounds**	**50 μM**	**125 μM**	**250 μM**	**500 μM**	**1000 μM**
**DUAL**	-70,15	-39,24	-41,87	-60,39	-70,15
**2**	-100,00	-100,00	-100,00	-100,00	-100,00
**3**	-100,00	-100,00	-100,00	-100,00	-100,00
**4**	-100,00	-6,67	-4,33	-100,00	-100,00
**5**	-100,00	-25,33	-22,00	-100,00	-100,00
**6**	-100,00	-51,56	-42,67	-100,00	-100,00
**7**	-100,00	-33,33	-40,00	-100,00	-100,00
**8**	-2,33	-83,83	-20,89	-57,33	-2,33
**9**	-48,89	-77,05	-20,05	-29,48	-48,89
**10**	-56,00	-21,80	-50,44	-51,33	-56,00
**11**	-48,00	-74,98	-100,00	-39,00	-48,00
**12**	-68,83	-25,93	-60,67	-28,40	-68,83
**13**	-100,00	-13,33	-92,67	-100,00	-100,00
**14**	-60,67	-99,87	-27,78	-12,00	-60,67
**15**	-16,00	-11,87	-48,67	-100,00	-16,00
**16**	-100,00	-65,33	-100,00	-100,00	-100,00
**17**	-32,00	-32,67	-46,93	-1,90	-32,00
**18**	-2,67	-100,00	-100,00	-63,00	-2,67
**19**	-4,00	-73,11	-99,60	-42,33	-4,00

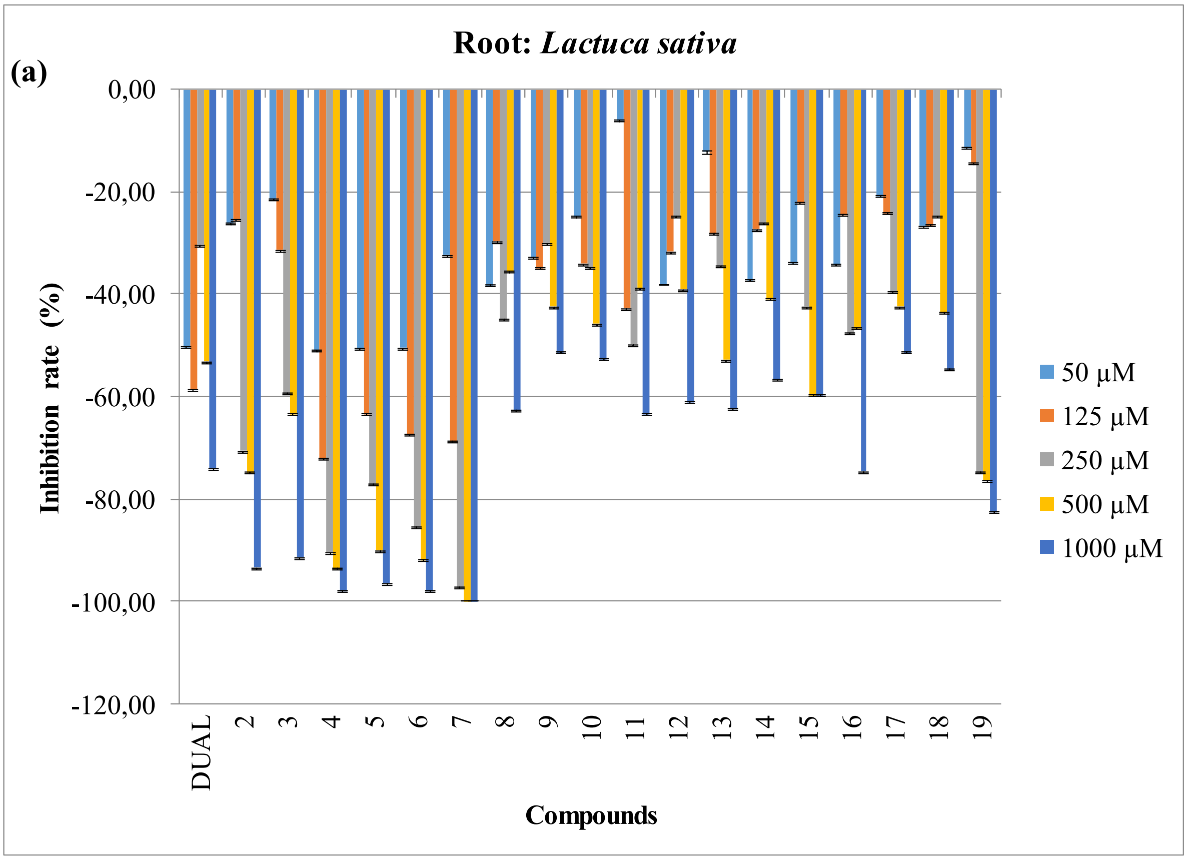

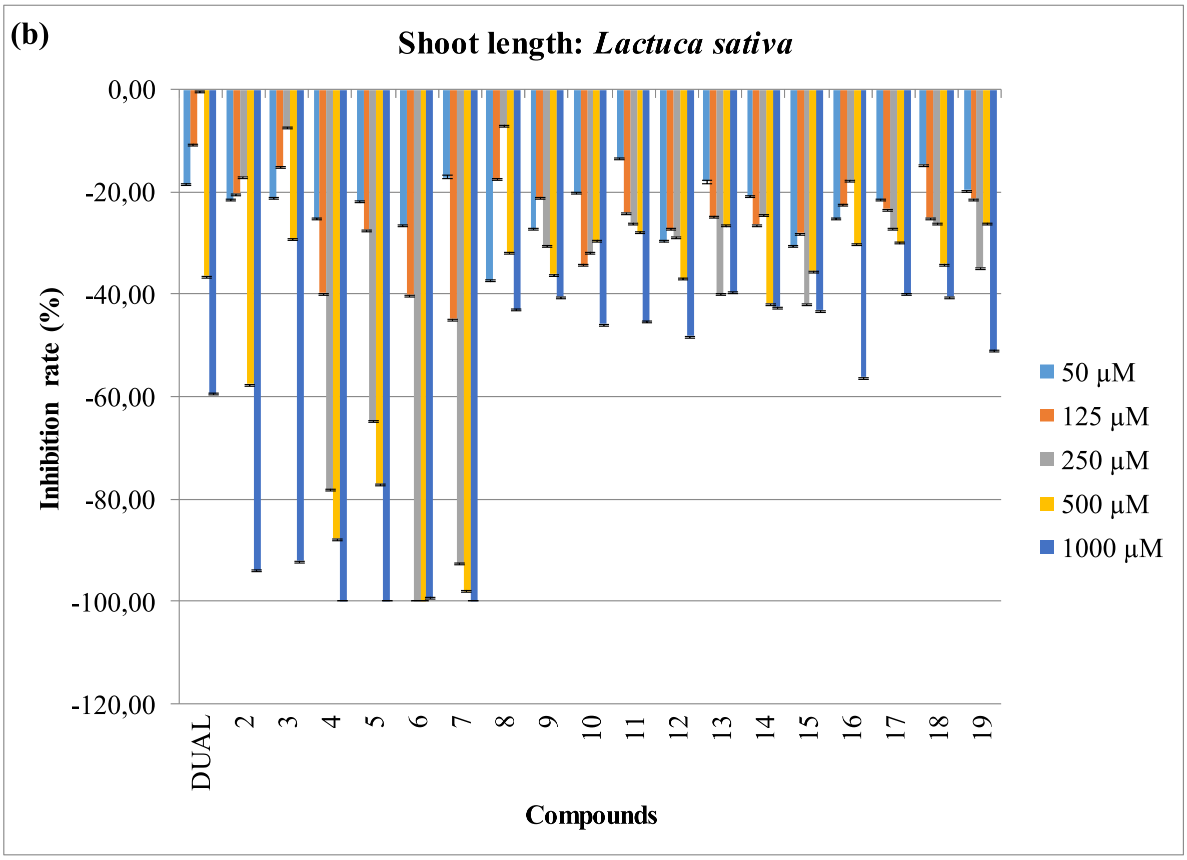

Figure S77. (a) Root and (b) shoot length of *Lactuca sativa spp*. in relation to the control

solution. Values are expressed as the percentage difference from the control. The error bars represent the standard deviation.

Table S3. Root inhibition rate of compounds **2-19** against *Lactuca sativa spp.* seedlings.

	**Root length**				
**Compounds**	**50 μM**	**125 μM**	**250 μM**	**500 μM**	**1000 μM**
**DUAL**	-50,33	-58,50	-30,48	-53,21	-74,07
**2**	-26,36	-25,50	-70,60	-74,93	-93,40
**3**	-21,48	-31,40	-59,45	-63,48	-91,43
**4**	-51,12	-72,17	-90,40	-93,55	-97,98
**5**	-50,74	-63,50	-77,19	-90,10	-96,52
**6**	-50,57	-67,45	-85,60	-91,95	-97,93
**7**	-32,38	-68,74	-97,33	-100,00	-100,00
**8**	-38,31	-29,76	-44,95	-35,69	-62,76
**9**	-32,90	-34,86	-30,12	-42,50	-51,45
**10**	-25,00	-34,05	-34,86	-45,81	-52,81
**11**	-6,21	-42,86	-49,90	-38,90	-63,38
**12**	-38,29	-31,81	-24,88	-39,14	-61,02
**13**	-12,33	-28,24	-34,55	-52,93	-62,48
**14**	-37,36	-27,38	-26,29	-40,98	-56,81
**15**	-34,14	-22,17	-42,48	-59,83	-59,69
**16**	-34,33	-24,52	-47,64	-46,81	-74,71
**17**	-20,79	-24,12	-39,57	-42,48	-51,36
**18**	-26,71	-26,60	-24,86	-43,64	-54,67
**19**	-11,45	-14,60	-74,83	-76,29	-82,55

Table S4. Shoot inhibition rate of compounds **2-19** against *Lactuca sativa spp.* seedlings.

	**Shoot length**				
**Compounds**	**50 μM**	**125 μM**	**250 μM**	**500 μM**	**1000 μM**
**DUAL**	-18,55	-10,70	-0,47	-36,50	-59,43
**2**	-21,50	-20,40	-17,03	-57,63	-94,03
**3**	-21,33	-15,20	-7,40	-29,17	-92,13
**4**	-25,27	-39,80	-78,23	-87,80	-100,00
**5**	-22,07	-27,37	-64,70	-77,03	-100,00
**6**	-26,60	-40,13	-100,00	-100,00	-99,37
**7**	-17,20	-44,90	-92,53	-97,90	-100,00
**8**	-37,37	-17,47	-7,03	-31,90	-43,00
**9**	-27,20	-21,17	-30,60	-36,17	-40,47
**10**	-20,27	-34,33	-31,93	-29,50	-45,80
**11**	-13,40	-24,10	-26,00	-27,93	-45,40
**12**	-29,70	-27,30	-28,77	-37,00	-48,23
**13**	-18,03	-24,93	-39,90	-26,70	-39,70
**14**	-20,83	-26,43	-24,60	-41,80	-42,47
**15**	-30,63	-28,03	-41,87	-35,50	-43,13

**16**	-25,20	-22,50	-17,73	-30,17	-56,27
**17**	-21,53	-23,33	-27,23	-30,00	-40,00
**18**	-14,63	-24,97	-26,00	-34,17	-40,47
**19**	-19,70	-21,30	-34,73	-26,23	-51,10

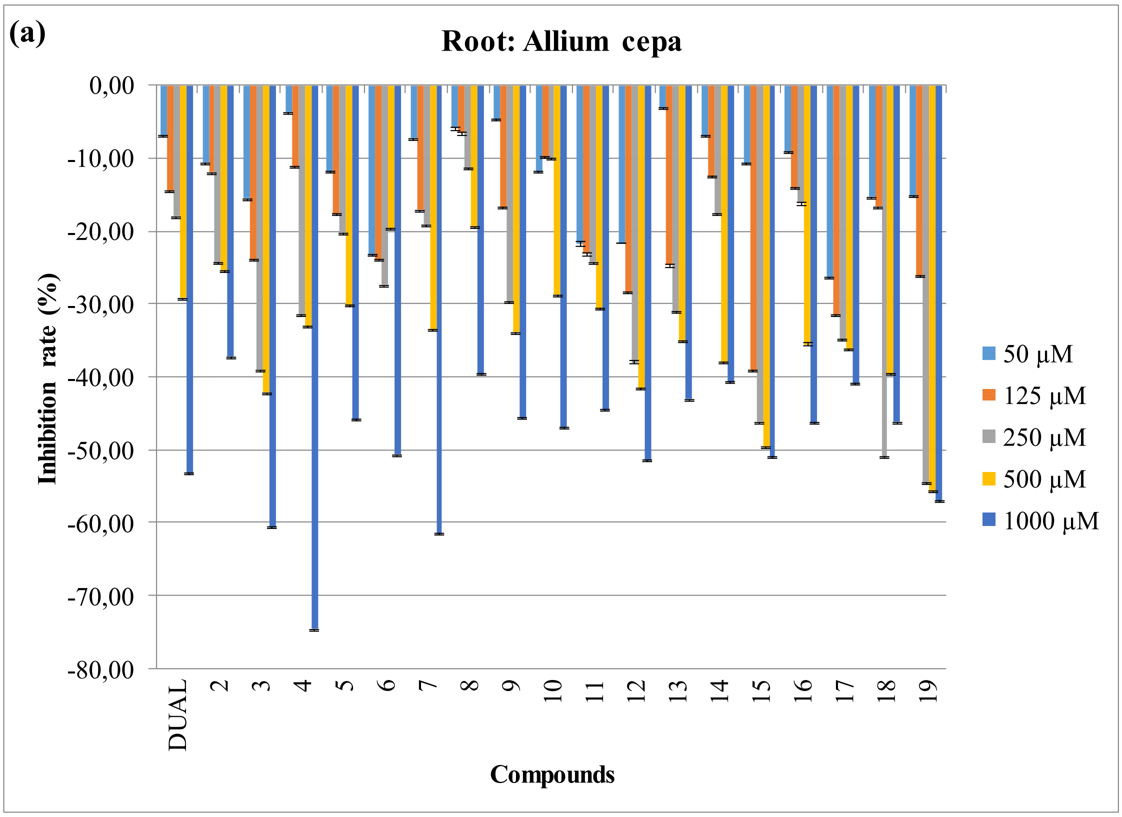
(a)
Root: Allium cepa
Inhibition rate (%)
0,00
-10,00
-20,00
-30,00
-40,00
-50,00
-60,00
-70,00
-80,00
DUAL
2
3
4
5
6
7
8
9
10
11
12
13
14
15
16
17
18
19
Compounds
50 µM
125 µM
250 µM
500 µM
1000 µM

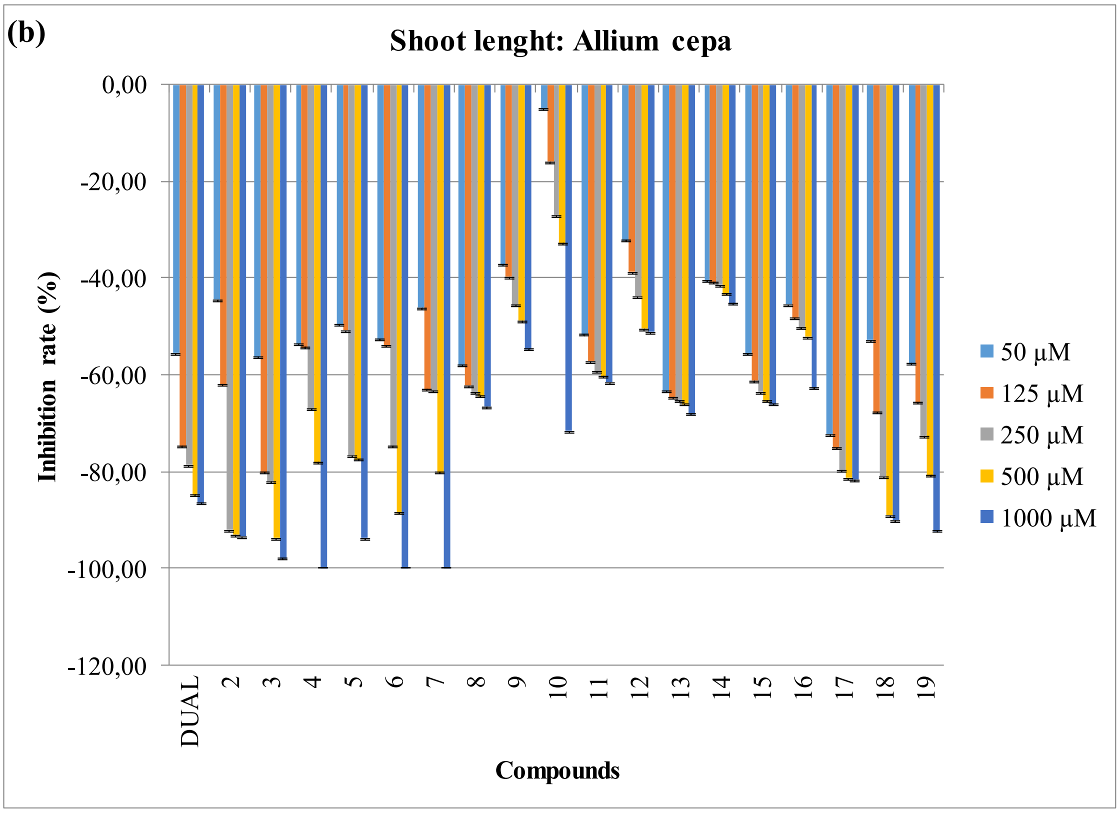
(b)
Shoot lenght: Allium cepa
Inhibition rate (%)
0,00
-20,00
-40,00
-60,00
-80,00
-100,00
-120,00
DUAL
2
3
4
5
6
7
8
9
10
11
12
13
14
15
16
17
18
19
Compounds
50 µM
125 µM
250 µM
500 µM
1000 µM

Figure S78. (a) Root and (b) shoot length of *Allium cepa spp.* in relation to the control solution. Values are expressed as the percentage difference from the control. The error bars represent the standard deviation.

Table S5. Shoot inhibition rate of compounds **2-19** against *Allium cepa spp.* seedlings.

	**Shoot inhibition**				
**Compounds**	**50 μM**	**125 μM**	**250 μM**	**500 μM**	**1000 μM**
**DUAL**	-7,05	-14,62	-18,02	-29,21	-53,33
**2**	-10,67	-12,05	-24,38	-25,57	-37,26
**3**	-15,60	-24,07	-39,07	-42,21	-60,71
**4**	-3,74	-11,29	-31,60	-33,14	-74,64
**5**	-11,86	-17,76	-20,24	-30,29	-45,93
**6**	-23,31	-24,00	-27,45	-19,74	-50,69
**7**	-7,29	-17,14	-19,14	-33,57	-61,55
**8**	-6,00	-6,62	-11,45	-19,55	-39,74
**9**	-4,76	-16,79	-29,76	-33,93	-45,67
**10**	-12,02	-9,88	-10,05	-28,81	-46,98
**11**	-21,79	-23,19	-24,33	-30,62	-44,57
**12**	-21,71	-28,48	-38,02	-41,62	-51,52
**13**	-3,14	-24,79	-31,10	-35,12	-43,05
**14**	-7,00	-12,57	-17,81	-38,05	-40,74
**15**	-10,81	-39,21	-46,21	-49,67	-51,05
**16**	-9,17	-14,14	-16,26	-35,62	-46,33
**17**	-26,43	-31,60	-34,98	-36,21	-40,95
**18**	-15,38	-16,74	-51,05	-39,67	-46,21
**19**	-15,26	-26,24	-54,57	-55,74	-57,07

Table S6. Root inhibition rate of compounds **2-19** against *Allium cepa spp.* seedlings.

	**Root inhibition**				
**Compounds**	**50 μM**	**125 μM**	**250 μM**	**500 μM**	**1000 μM**
**DUAL**	-55,50	-74,83	-78,77	-84,90	-86,50
**2**	-44,43	-62,03	-92,17	-93,13	-93,47
**3**	-56,47	-80,17	-82,07	-93,73	-97,93
**4**	-53,73	-54,40	-67,17	-78,27	-100,00
**5**	-49,70	-50,80	-76,70	-77,53	-93,80
**6**	-52,63	-54,10	-74,90	-88,67	-100,00
**7**	-46,13	-63,10	-63,40	-80,20	-100,00
**8**	-58,00	-62,27	-63,80	-64,40	-66,60
**9**	-37,27	-39,83	-45,73	-48,83	-54,70
**10**	-5,17	-15,97	-27,07	-32,97	-71,70
**11**	-51,80	-57,23	-59,47	-60,33	-61,60
**12**	-32,30	-38,73	-43,80	-50,60	-51,27
**13**	-63,47	-64,83	-65,37	-66,17	-68,20

**14**	-40,57	-40,97	-41,53	-43,27	-45,33
**15**	-55,67	-61,30	-63,57	-65,40	-66,03
**16**	-45,50	-48,37	-50,33	-52,20	-62,70
**17**	-72,37	-75,10	-79,83	-81,37	-81,77
**18**	-52,90	-67,77	-81,10	-89,03	-90,13
**19**	-57,77	-65,90	-72,73	-80,93	-92,37

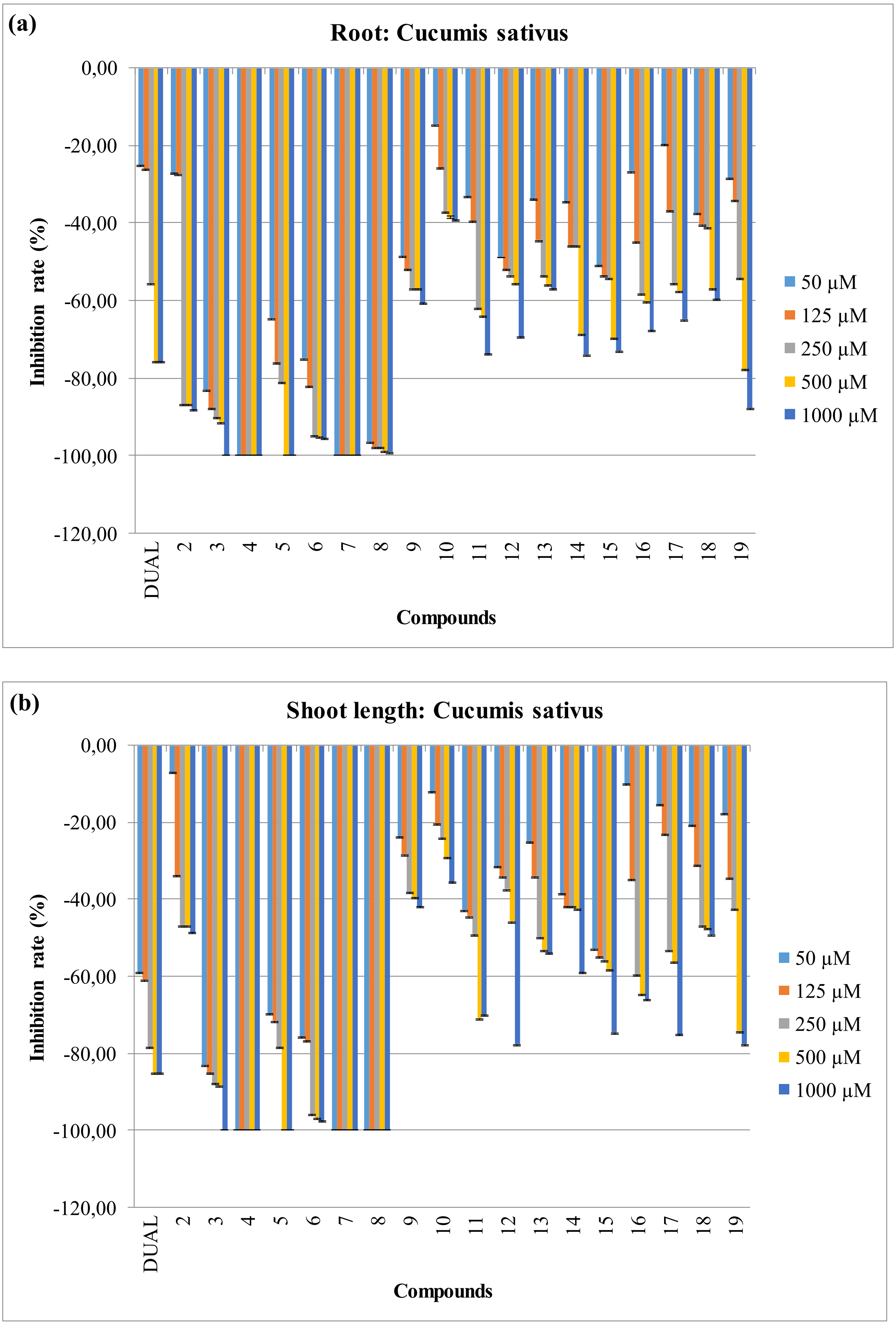

Figure S79. (a) Root and (b) shoot length of *Cucumis sativus spp.* in relation to the control solution. Values are expressed as the percentage difference from the control. The error bars represent the standard deviation.

Table S7. Shoot inhibition rate of compounds **2-19** against *Cucumis sativus spp.* seedlings.

	Shoot inhibition				
**Compounds**	**50 μM**	**125 μM**	**250 μM**	**500 μM**	**1000 μM**
**DUAL**	-25,21	-26,26	-55,55	-75,88	-75,88
**2**	-27,19	-27,45	-86,93	-86,93	-88,29
**3**	-83,07	-87,98	-90,36	-91,43	-100,00
**4**	-100,00	-100,00	-100,00	-100,00	-100,00
**5**	-64,67	-76,24	-81,17	-100,00	-100,00
**6**	-75,07	-82,19	-95,00	-95,17	-95,57
**7**	-100,00	-100,00	-100,00	-100,00	-100,00
**8**	-96,43	-97,79	-98,07	-98,86	-99,12
**9**	-48,50	-52,12	-56,88	-57,10	-60,79
**10**	-14,67	-25,98	-37,26	-38,60	-39,31
**11**	-33,33	-39,60	-62,07	-64,21	-73,95
**12**	-49,07	-52,02	-53,55	-55,76	-69,55
**13**	-33,86	-44,55	-53,90	-56,02	-57,02
**14**	-34,57	-46,07	-46,07	-68,62	-74,07
**15**	-51,05	-53,83	-54,24	-69,83	-72,98
**16**	-26,67	-45,12	-58,45	-60,57	-67,81
**17**	-19,90	-37,00	-55,76	-57,62	-64,98
**18**	-37,52	-40,50	-41,21	-57,17	-59,79
**19**	-28,64	-34,31	-54,31	-77,93	-87,76

Table S8. Root inhibition rate of compounds **2-19** against *Cucumis sativus spp.* seedlings.

	Root inhibition				
**Compounds**	**50 μM**	**125 μM**	**250 μM**	**500 μM**	**1000 μM**
**DUAL**	-59,17	-61,00	-78,57	-85,17	-85,17
**2**	-7,07	-33,92	-46,83	-46,83	-48,60
**3**	-83,13	-85,23	-87,87	-88,37	-100,00
**4**	-100,00	-100,00	-100,00	-100,00	-100,00
**5**	-69,93	-71,73	-78,60	-100,00	-100,00
**6**	-75,87	-76,83	-95,97	-96,80	-97,63
**7**	-100,00	-100,00	-100,00	-100,00	-100,00
**8**	-100,00	-100,00	-100,00	-100,00	-100,00
**9**	-23,87	-28,37	-38,33	-39,70	-42,00
**10**	-12,23	-20,63	-24,27	-29,20	-35,53
**11**	-42,83	-44,43	-49,33	-71,03	-70,13
**12**	-31,67	-34,13	-37,50	-45,87	-77,73
**13**	-25,30	-34,17	-49,87	-53,27	-54,20
**14**	-38,57	-42,03	-42,03	-42,50	-59,07
**15**	-52,87	-55,13	-55,87	-58,47	-74,63

**16**	-10,13	-34,93	-59,67	-64,88	-66,07
**17**	-15,53	-23,20	-53,17	-56,43	-75,17
**18**	-20,93	-31,17	-46,80	-47,60	-49,23
**19**	-17,80	-34,60	-42,70	-74,37	-77,77

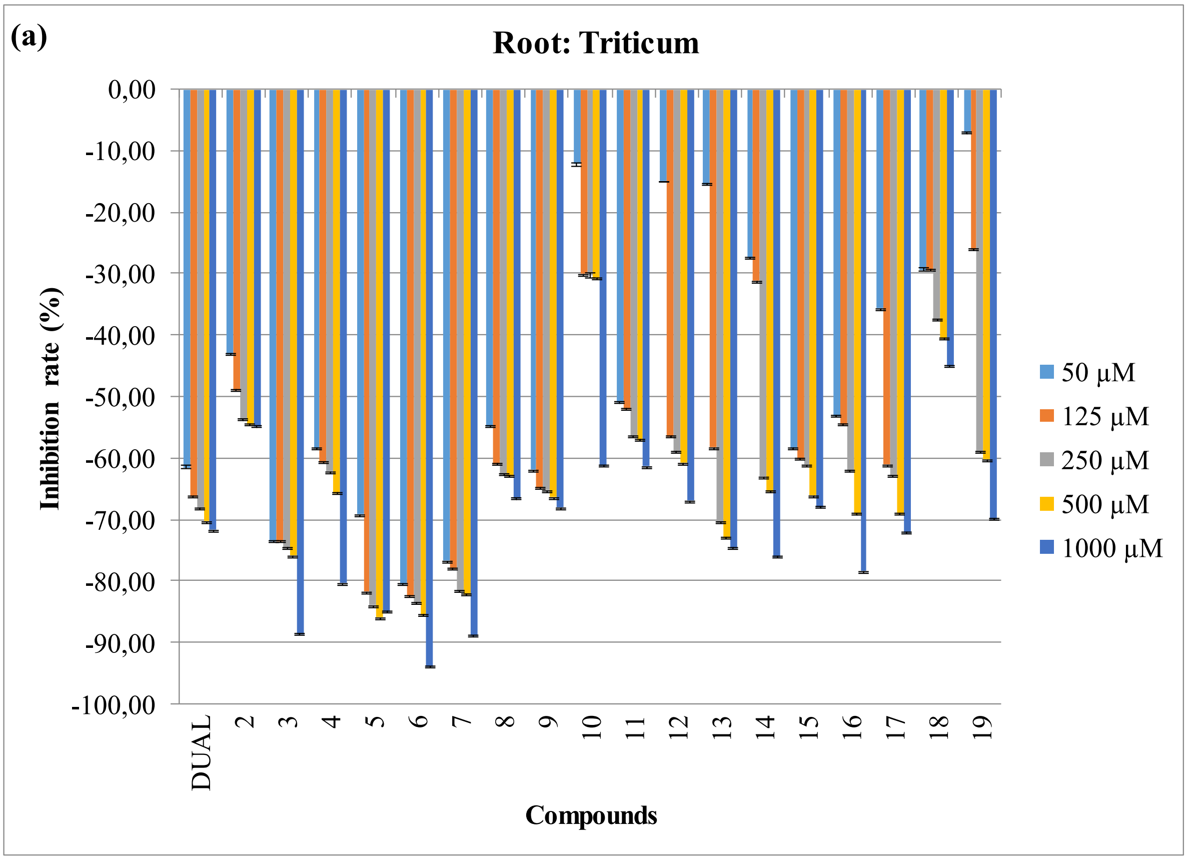
(a)
Root: Triticum
0,00
-10,00
-20,00
-30,00
-40,00
-50,00
-60,00
-70,00
-80,00
-90,00
-100,00
Inhibition rate (%)
DUAL
2
3
4
5
6
7
8
9
10
11
12
13
14
15
16
17
18
19
Compounds
50 µM
125 µM
250 µM
500 µM
1000 µM

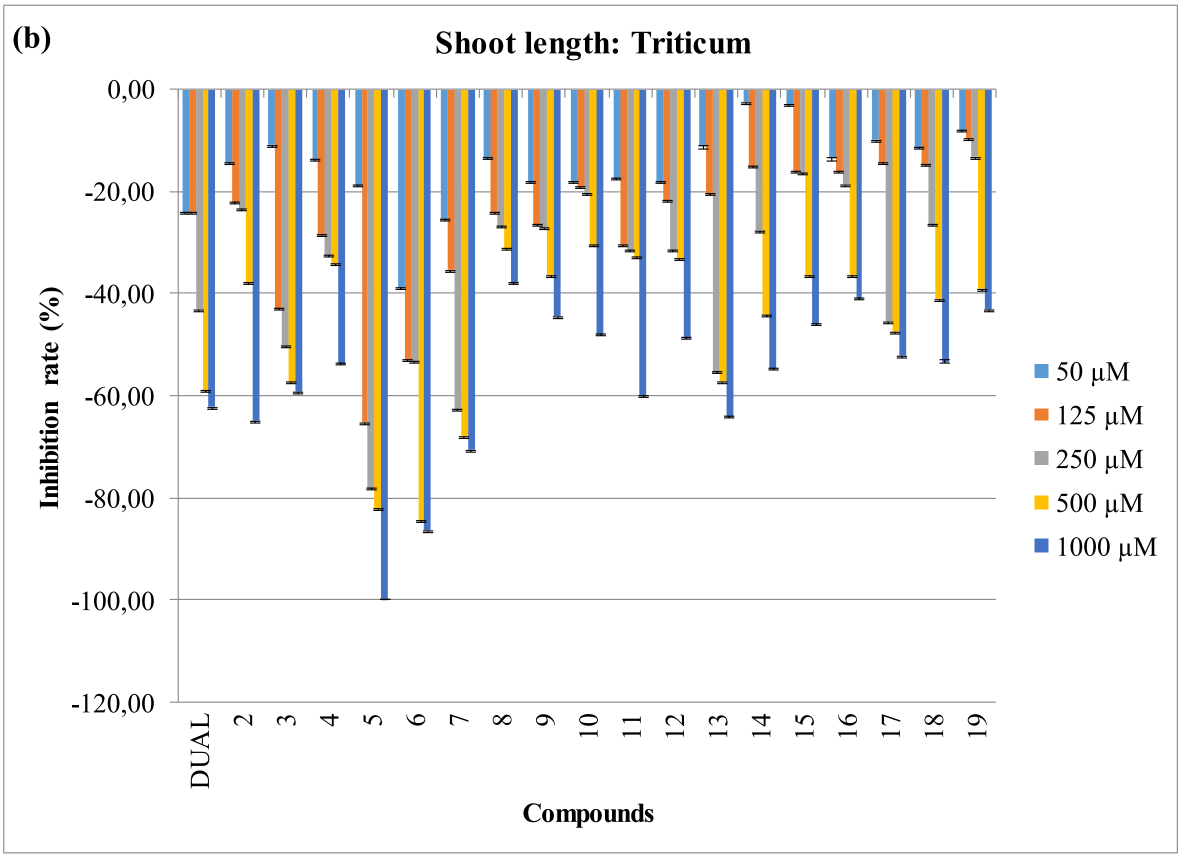
(b)
Shoot length: Triticum
0,00
-20,00
-40,00
-60,00
-80,00
-100,00
-120,00
Inhibition rate (%)
DUAL
2
3
4
5
6
7
8
9
10
11
12
13
14
15
16
17
18
19
Compounds
50 µM
125 µM
250 µM
500 µM
1000 µM

Figure S80. (a) Root and (b) shoot length of *Triticum aestivu*m in relation to the control solution. Values are expressed as the percentage difference from the control. The error bars represent the standard deviation.

Table S9. Root inhibition rate of compounds **2-19** against *Triticum aestivu*m seedlings.

	**Root inhibition**				
**Compounds**	**50 μM**	**125 μM**	**250 μM**	**500 μM**	**1000 μM**
**DUAL**	-61,52	-66,33	-68,29	-70,36	-71,76
**2**	-43,14	-48,93	-53,86	-54,55	-54,90
**3**	-73,50	-73,60	-74,60	-75,98	-88,69
**4**	-58,48	-60,67	-62,26	-65,67	-80,40
**5**	-69,26	-81,83	-84,05	-86,02	-85,02
**6**	-80,50	-82,43	-83,60	-85,60	-94,02
**7**	-76,79	-78,00	-81,74	-82,24	-89,00
**8**	-54,88	-60,98	-62,74	-62,86	-66,64
**9**	-61,98	-64,93	-65,36	-66,48	-68,12
**10**	-12,21	-30,10	-30,38	-30,83	-61,29
**11**	-51,00	-52,02	-56,43	-57,12	-61,40
**12**	-14,93	-56,55	-58,95	-60,81	-67,02
**13**	-15,48	-58,29	-70,52	-73,02	-74,60
**14**	-27,55	-31,21	-63,12	-65,52	-76,10
**15**	-58,50	-60,07	-61,14	-66,14	-68,05
**16**	-53,14	-54,55	-62,17	-69,17	-78,50
**17**	-35,71	-61,10	-63,02	-69,02	-72,14
**18**	-29,33	-29,43	-37,55	-40,55	-45,07
**19**	-7,12	-26,17	-59,12	-60,33	-69,98

Table S10. Root inhibition rate of compounds **2-19** against *Triticum aestivu*m seedlings.

	**Root inhibition**				
**Compounds**	**50 μM**	**125 μM**	**250 μM**	**500 μM**	**1000 μM**
**DUAL**	-24,20	-24,20	-43,20	-59,07	-62,23
**2**	-14,47	-22,30	-23,70	-38,10	-64,93
**3**	-11,00	-42,90	-50,37	-57,33	-59,23
**4**	-13,87	-28,67	-32,50	-34,37	-53,67
**5**	-18,90	-65,27	-78,23	-82,23	-100,00
**6**	-38,97	-53,03	-53,30	-84,60	-86,37
**7**	-25,53	-35,50	-62,80	-68,00	-70,63
**8**	-13,53	-24,37	-26,93	-31,33	-37,90
**9**	-18,20	-26,63	-27,37	-36,50	-44,47
**10**	-18,27	-19,27	-20,60	-30,80	-48,07
**11**	-17,50	-30,50	-31,50	-32,90	-60,13
**12**	-18,13	-22,00	-31,53	-33,40	-48,73
**13**	-11,30	-20,63	-55,40	-57,23	-64,23

**14**	-2,63	-15,23	-27,80	-44,23	-54,70
**15**	-3,23	-16,07	-16,50	-36,57	-45,93
**16**	-13,70	-16,30	-18,67	-36,53	-41,03
**17**	-9,97	-14,50	-45,57	-47,50	-52,20
**18**	-11,40	-14,87	-26,53	-41,17	-53,30
**19**	-8,13	-9,70	-13,27	-39,20	-43,10
